# Semisynthesis and Cytotoxic Evaluation of an Ether Analogue Library Based on a Polyhalogenated Diphenyl Ether Scaffold Isolated from a *Lamellodysidea* Sponge

**DOI:** 10.3390/md22010033

**Published:** 2024-01-03

**Authors:** Kelsey S. Ramage, Aaron Lock, Jonathan M. White, Merrick G. Ekins, Milton J. Kiefel, Vicky M. Avery, Rohan A. Davis

**Affiliations:** 1 Griffith Institute for Drug Discovery, School of Environment and Science, Griffith University, Brisbane, QLD 4111, Australia; kelsey.ramage@griffithuni.edu.au (K.S.R.); merrick.ekins@qm.qld.gov.au (M.G.E.); 2 Discovery Biology, School of Environment and Science, Griffith University, Brisbane, QLD 4111, Australia; a.lock@griffith.edu.au (A.L.); v.avery@griffith.edu.au (V.M.A.); 3 School of Chemistry and Bio21 Institute, The University of Melbourne, Parkville, VIC 3010, Australia; whitejm@unimelb.edu.au; 4 Queensland Museum, South Brisbane, QLD 4101, Australia; 5 Institute for Glycomics, School of Environment and Science, Griffith University, Gold Coast, QLD 4222, Australia; m.kiefel@griffith.edu.au; 6 NatureBank, Griffith Institute for Drug Discovery, Griffith University, Nathan, QLD 4111, Australia

**Keywords:** *Lamellodysidea*, Dysideidae, natural product, oxygenated polyhalogenated diphenyl ether, semisynthesis, prostate cancer, breast cancer

## Abstract

The known oxygenated polyhalogenated diphenyl ether, 2-(2′,4′-dibromophenoxy)-3,5-dibromophenol (**1**), with previously reported activity in multiple cytotoxicity assays was isolated from the sponge *Lamellodysidea* sp. and proved to be an amenable scaffold for semisynthetic library generation. The phenol group of **1** was targeted to generate 12 ether analogues in low-to-excellent yields, and the new library was fully characterized by NMR, UV, and MS analyses. The chemical structures for **2**, **8**, and **9** were additionally determined via single-crystal X-ray diffraction analysis. All natural and semisynthetic compounds were evaluated for their ability to inhibit the growth of DU145, LNCaP, MCF-7, and MDA-MB-231 cancer cell lines. Compound **3** was shown to have near-equivalent activity compared to scaffold **1** in two in vitro assays, and the activity of the compounds with an additional benzyl ring appeared to be reliant on the presence and position of additional halogens.

## 1. Introduction

Dysideidae sponges, such as those in the genus *Lamellodysidea*, are known to contain a wide variety of compounds, which generally are categorized into two distinct chemotypes: those containing highly functionalized peptides and terpenoids, and those solely containing oxygenated polyhalogenated diphenyl ethers (O-PHDEs) [1,2]. It has long been understood that while the latter chemotypes are structurally similar to anthropogenic fire retardants, there is extensive evidence for their presence in sponges to be due to their antimicrobial and antifeedant activity [3,4,5,6,7]. It is now accepted knowledge that O-PHDEs are produced by symbiotic cyanobacteria within marine sponges, with the first evidence of this claim being published in 1994, where it was found that O-PHDEs from *Lamellodysidea herbacea* were produced by the cyanobacterial symbiont *Hormoscilla spongeliae* and excreted into the aqueous sponge mesophyll [2].

**Figure 1:**
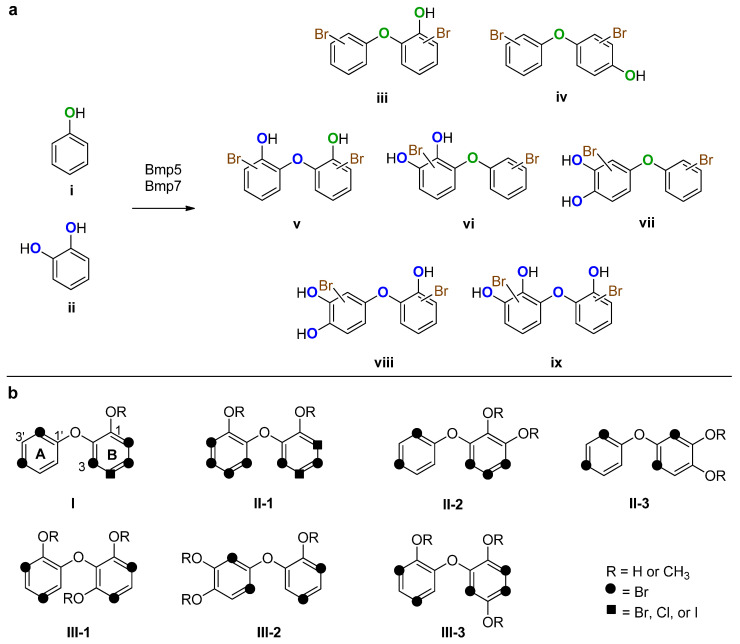
(**a**) Generalized process for the biosynthesis of O-PHDEs. Phenol (**i**) and catechol (**ii**) are brominated with Bmp5 (halogens omitted from **iii**–**ix** for clarity) prior to Bmp7 bromophenol homocoupling (**iii**–**iv**), bromophenol–bromocatechol heterocoupling (**v**–**vii**), or bromocatechol homocoupling (**viii**–**ix**) [8,9]. Note that no O-PHDE with the oxygenation pattern described as **ix** has been reported to date. (**b**) Substitution patterns of all PHDEs isolated from Dysideidae sponges to date. Note that compounds with the oxygenation pattern **III-3** cannot be generated solely through the biosynthetic process described in Figure 1a, suggesting that post-translational oxidases or hydroxylases may play a role in this case [9,10].

Since this initial research, much of the biosynthetic pathway for these compounds has been described following research into various proteobacteria associated with marine eukaryotes [1,8,9]. The brominated marine pyrroles/phenols (bmp) bacterial gene locus contains two key enzymes: phenol brominase Bmp5, which is able to incorporate bromine or iodine into phenol and catechol radicals; and cytochrome P450 Bmp7, which mediates the homo or heterocoupling of bromophenol and bromocatechol (Figure 1a) [8,9]. Promiscuous “off-pathway” chlorinases and brominases can selectively chlorinate, brominate, or iodinate the structure post-coupling (Figure 1b), with the scarceness of iodinated O-PHDEs being explained by the greater abundance of chloride and bromide in seawater [1,9]. While diversity in halogenating enzymes, differential coupling of bromophenol and bromocatechol, and methylation of phenolic groups allows for an extraordinary diversity of substitution patterns in these compounds, only one iodinated O-PHDE has been reported to date, and it appears that the halogen substitution patterns observed in known O-PHDEs are dictated by biosynthetic rules which have not been defined [1,3,8,9,10].

Due to the Davis group’s interest in the use of new or under-utilized natural products as scaffolds for the semisynthesis of unique biodiscovery screening libraries, a new species of *Lamellodysidea* sponge (that is in the process of being fully taxonomically described) was prioritized for chemical investigations [11,12]. The CH_2_Cl_2_:MeOH extract from this specimen showed only one major polybrominated UV-active peak via UHPLC-MS (Supplementary Figure S1) that was subsequently identified as the known O-PHDE, 2-(2′,4′-dibromophenoxy)-3,5-dibromophenol (**1**), a bioactive scaffold amenable to semisynthesis [13]. In previous studies, **1** had been isolated from *Dysidea* and *Lamellodysidea* sponges and reported by other researchers to not only have antibacterial and antifeedant activity but also broad cytotoxicity, including moderate activity (IC_50_ ≤ 10 µM) in PANC-1 (epithelioid carcinoma), MCF-7 (triple-positive breast cancer), and BS-C-1 (continuous epithelial kidney cell) cancer cell lines and moderate activity against the cancer-relevant proteins Tie2 kinase, PTP1B, and IMPDH [3,4,5,6,14,15,16,17,18]. In the current studies reported here, the reactive phenol group of **1** was exploited to generate a novel O-PHDE library for continued investigations into this structure class’s activity against MCF-7, in addition to activity against MDA-MB-231 (triple-negative breast cancer), DU145 (prostate carcinoma) and LNCaP (androgen-sensitive human prostate adenocarcinoma) cell lines [19,20].

## 2. Results and Discussion

To obtain sufficient quantities of scaffold **1** for semisynthetic studies, an exhaustive extraction of dried and homogenized *Lamelloysidea* sp. (5 g) was undertaken using a previously reported method that involved extraction with *n*-hexane and CH_2_Cl_2_ [21], followed by silica flash chromatography (*n*-hexane/EtOAc). This process afforded **1** in high yield (221.3 mg, 4.43% dry wt). A comparison of NMR and MS data with literature values confirmed that the known compound, 2-(2′,4′-dibromophenoxy)-3,5-dibromophenol (**1**), had been obtained [13,16]. 

**Scheme 1:**
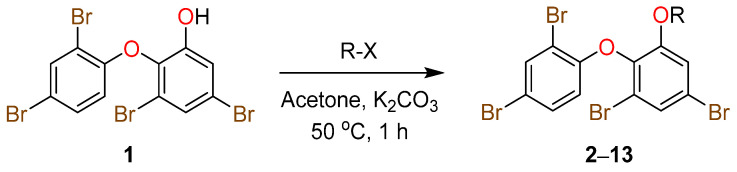
Reaction conditions and reagents used to generate the semisynthetic library (**2**–**13**).

**Figure 2:**
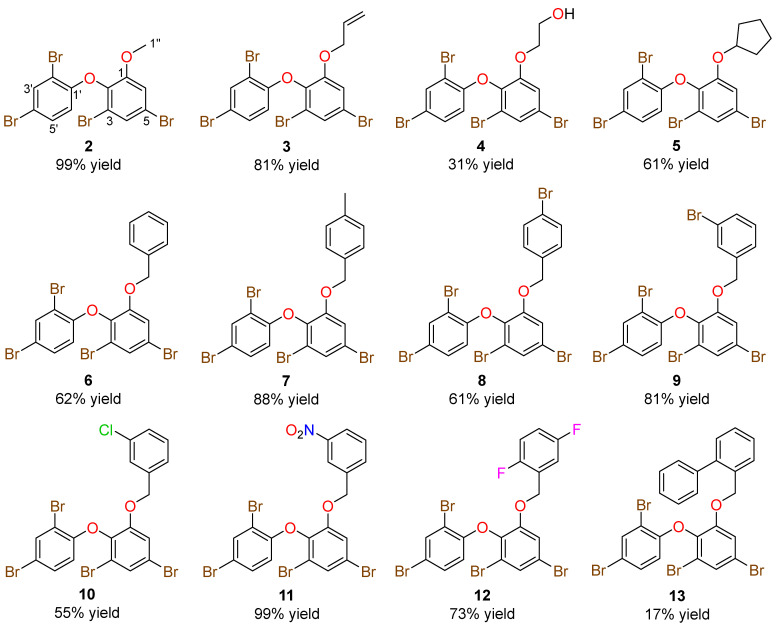
Semisynthetics **2**–**13** that were generated from scaffold **1**.

Scaffold **1** (10 mg) was subsequently reacted with a series of commercially available alkyl halides (R-X) in dry acetone for 1 h at 50 °C, using K_2_CO_3_ as a base (Scheme 1 and Figure 2). The reactions were initially tested in laboratory-grade and deuterated acetone, where it was found that deuterated acetone was sufficiently dry for the reactions to take place with low-to-excellent yields. Following a workup with CH_2_Cl_2_/H_2_O partitioning and HPLC purification (MeOH/H_2_O/0.1% TFA), a total of 12 analogues (**2**–**13**), including one naturally occurring O-PHDE (**2**) [22], were obtained in yields ranging from 17 to 99%, and purities of >95%, as determined by UHPLC-MS analysis. Furthermore, all O-PHDE derivatives were fully characterized via 1D/2D NMR, UV, and MS data analyses.

**Figure 3:**
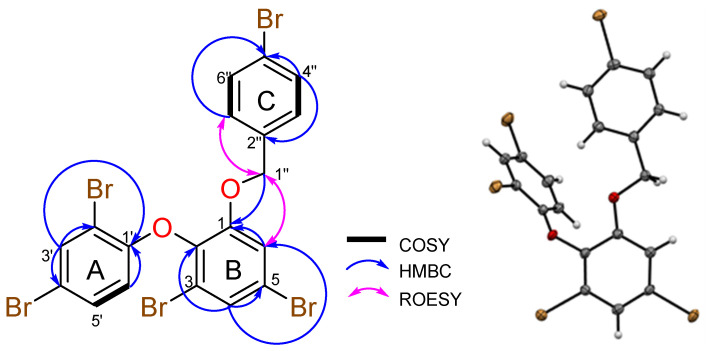
Key 2D NMR correlations (**left**) and ORTEP (**right**) drawing of O-PHDE semisynthetic **8**.

**Figure 4:**
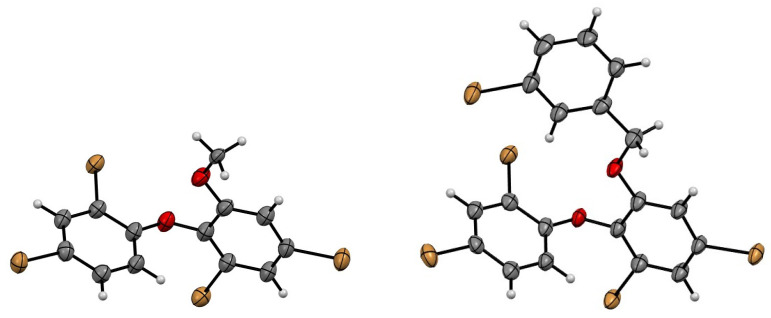
ORTEP drawings of O-PHDE semisynthetics **2** (**left**) and **9** (**right**).

For example, the HRESIMS of O-PHDE analogue **8** revealed an ion at *m*/*z* 688.6566 [M + Na]^+^ that enabled a molecular formula of C_19_H_11_^79^Br_5_O_2_ to be assigned to the new semisynthetic. The ^1^H NMR spectrum (Table 1) of **8** in DMSO-*d*_6_ indicated the presence of five aromatic protons from the scaffold [δ_H_ 7.90, 7.63, 7.56, 7.41, 6.53], four additional aromatic protons [δ_H_ 7.48 (2H), 7.05 (2H)], and two methylene protons [δ_H_ 5.12 (2H)]. The ^13^C NMR and edited HSQC spectra of **8** (Supplementary Figures S46 and S48) indicated a total of 17 unique carbon signals, including 12 aromatic carbons from the scaffold [δ_C_ 152.7, 151.9, 139.8, 135.2, 131.7, 127.1, 119.1, 117.9, 117.8, 116.3, 114.4, 111.8], four additional aromatic carbon signals [δ_C_ 135.2, 131.2 (2C), 129.3 (2C), 121.2], and a methylene carbon [δ_C_ 69.8]. The methylene resonating at δ_H_ 5.12 (H-1″) showed strong ROESY and HMBC correlations to H-6 and C-1 respectively, which connected ring B of the O-PHDE scaffold to ring C (Figure 3). The remaining NMR signals for **8** were assigned following extensive NMR data analysis and a comparison of the chemical shifts for the previously reported scaffold **1** (Table 1 and Figure 3). Furthermore, crystals obtained for compound **8** were analyzed via X-ray crystallography and confirmed the NMR-based structure assignment; the ORTEP for **8** is shown below in Figure 3. Additionally, crystals obtained for compounds **2** and **9** were also analyzed via X-ray crystallography, which confirmed their NMR-based structure assignments; the ORTEPs for **2** and **9** are shown below in Figure 4. 

Based on previous cytotoxic activity reported for scaffold **1**, the natural products **1** and **2** and synthetic analogues **3**–**13** were screened for activity against four cancer cell lines (Table 2). While scaffold **1** and its derivatives displayed low activity in the DU145 assay, the activity reported in the other cell lines was sufficient to allow for some structure–activity relationships (SARs) to be determined. While most compounds with benzylated additions (**6**–**13**) displayed minimal activity in all assays, some preliminary SARs were identified based on the type and positioning of substituents on ring C. For example, compounds **7** and **8** that are methylated and brominated in the *para*-position of ring C respectively, displayed no activity, while **9**–**11** of the benzylated series, which all contain *meta*-positioned electron-withdrawing groups on ring C demonstrated some activity with the brominated analogue being the most toxic of the three compounds.

Compounds **12** and **13** each have *ortho*-substitutions on ring C and did not display appreciable activity thus indicating that this substitution pattern is detrimental to toxicity. Future libraries with varied positions and species of halogens and different functional groups are required in order to shed more light on SAR. Compound **3** displayed the best activity of all semisynthetic analogues tested, displaying indistinguishable cytotoxicity to scaffold **1** against MCF-7 and MDA-MB-231 breast cancer cell lines, which suggests that the addition of smaller alkyl groups was better for retaining bioactivity than the addition of benzyl rings.

In conclusion, 12 O-PHDE ether analogues (including one known naturally occurring compound) were synthesized in low-to-excellent yields, and the new library was characterized via NMR, US, and MS analyses. Whilst cytotoxicity evaluations identified no significant toxicity against four human cancer cell lines, this new library will be added to the Davis Open Access Natural Product-Based Library and screened in other bioassays in the future [23,24].

## 3. Materials and Methods

### 3.1. General Experimental

Melting points were measured using a Cole-Parmer melting point apparatus and are uncorrected. UV spectra were recorded using an Ocean Optics USB-ISS-UV/Vis spectrometer. NMR spectra were recorded at 25 °C on a Bruker AVANCE III HD 800 MHz NMR spectrometer (Billerica, MA, USA) equipped with a cryoprobe. The ^1^H and ^13^C chemical shifts were referenced to solvent peaks for DMSO-*d*_6_ at δ_H_ 2.50 and δ_C_ 39.52. HRESIMS data were acquired on a Bruker maXis II ETD ESI-qTOF. Silica gel 60 (Merck, 40–63 μm, 60 Å) was packed into an open glass column (38 × 90 mm) for flash column chromatography. Davisil C_8_ bonded silica (30–40 μM, 60 Å) was used for pre-adsorption work before HPLC separations, and the pre-absorbed sample was packed into a Grace stainless steel guard cartridge (10 × 30 mm). A Phenomenex Luna C_18_ column (5 µm, 90–110 Å, 10 mm × 250 mm) attached to a Thermo Fisher Scientific Dionex Ultimate 3000 UHPLC (Waltham, MA, USA) was used for semipreparative HPLC separations. A All chemical reagents used throughout the experiments were purchased from Sigma-Aldrich, and all solvents used for chromatography, UV, and MS were Honeywell Burdick & Jackson or Lab-Scan HPLC grade. NMR spectra were processed using MestReNova version 11.0.3 (Mestrelab Research, Santiago de Compostela, Spain). Chemical structures were drawn using ChemDraw Ultra 12.0.2. HPLC, and LC-MS results were analyzed via Thermo Scientific^TM^ Dionex^TM^ Chromeleon^TM^ 7.2. UV data were analyzed using Logger Pro 3 (Vernier Software & Technology, Beaverton, OR, USA). 

### 3.2. Animal Material

The undescribed *Lamellodysidea* sp. OTU 2054 specimen [12] was an olive green to light brown color underwater and turned brown to orange upon exposure to air, while producing copious black mucous. It formed thickly encrusted mats, with thick erect microconulose lamellae forming rounded labyrinthian meshes. The texture was rubbery, compressible, and soft, but it was firmly attached to the hard pavement directly behind the reef crest on Ribbon Reef (Great Barrier Reef, Queensland, Australia). The skeleton consisted of similarly sized cored primary and secondary fibers. A voucher specimen of this sponge, *Lamellodysidea* sp. OTU 2054 (QM G325118), has been deposited at the Queensland Museum.

### 3.3. Extraction and Isolation of O-PHDE **1**

Freeze-dried and ground *Lamellodysidea* sp. (5 g) was extracted sequentially with *n*-hexane (200 mL, 2 h) and CH_2_Cl_2_ (200 mL, 2 h), with shaking, at room temperature. The CH_2_Cl_2_ fraction contained scaffold **1** (127.5 mg, 95% purity, 2.55% dry wt). The *n*-hexane fraction (0.1455 g) was pre-adsorbed to silica gel 60 (Merck) and then chromatographed on a silica gel 60 column (38 × 91 mm), using a 10% stepwise gradient solvent system from 100% *n*-hexane to 100% EtOAc (50 mL flushes, 43 fractions collected in total), with fractions containing visible material analyzed via ^1^H NMR spectroscopy and UHPLC-MS. Fractions 14–17 that were eluted with 70% *n*-hexane/30% EtOAc contained the targeted scaffold **1** (93.8 mg, 95% purity, 1.88% dry wt).

### 3.4. General Preparation of Ether Derivatives from O-PHDE Scaffold **1**

A mixture of **1** (10 mg, 0.02 mmol) and anhydrous K_2_CO_3_ (553 mg, 4.0 mmol) was dissolved in dry deuterated acetone (0.5 mL). Excess alkyl halide (10 equiv., Supplementary Figure S2) was slowly added, and the mixture was stirred at 50 °C for 1 h. The completed reaction was left to dry via evaporation overnight, after which the mixture was dissolved in H_2_O (2 mL) and then extracted with CH_2_Cl_2_ (3 × 2 mL). Pure ether analogues **2** and **6** were obtained after solvent partitioning, with no further purification required. The CH_2_Cl_2_-soluble material, which was not purified by this partitioning process was absorbed to silica gel 60 (~1 g) and transferred to an Isolute™ Si SPE cartridge. Ethers **3**, **5**, **9**, and **10** were obtained using a two-step elution from 100% CH_2_Cl_2_ to 5% MeOH/95% CH_2_Cl_2_ (50 mL each, 8 fractions collected in total). The remaining ethers (**4**, **7**, **8**, and **11**–**13**) required further purification via semipreparative C_18_ HPLC, using a a linear gradient from 10% MeOH (0.1% TFA)/90% H_2_O (0.1% TFA) to 100% MeOH (0.1% TFA) over 60 min, at a flowrate of 4 mL/min.

### 3.5. X-ray Crystallography Analysis of Compounds **2**, **8**, and **9**

Intensity data for compound **8** were collected with an Oxford Diffraction Synergy diffractometer with Mo K*α* radiation, while data for **2** and **9** were collected at the MX2 beamline at the Australian Synchrotron [25]. The temperature during data collection was maintained at 100.0(1) K, using an Oxford Cryosystems cooling device. The structure of each compound was solved by using direct methods and difference Fourier synthesis [26]. Hydrogen atoms were placed in their idealized positions and included in subsequent refinement cycles. Thermal ellipsoid plots were generated in Mercury within the WINGX suite of programs [27,28]. Crystallographic data for **2**, **8,** and **9** were deposited with the Cambridge Crystallographic Data Centre and assigned the CCDC deposit codes 2307709, 2294089, and 2307710, respectively. These data can be obtained free of charge from the Cambridge Crystallographic Data Centre via http://www.ccdc.cam.ac.uk/data_request/cif, accessed on 15 June 2023.

Crystal data for compound **2**: M = 515.83, T = 100.0(10) K, λ = 0.710918 Å, Monoclinic, space group P21/n a = 12.955(3), b = 5.0960(10), c = 23.072(5) Å, β = 105.86(3)° V = 1465.2(5) Å^3^, Z = 4, D_c_ = 2.338 Mg M^−3^ μ = 10.980 mm^−1^, F(000) = 968, crystal size 0.20 × 0.02 × 0.02 mm^3^. θ_max_ = 32.160°, 25670 reflections measured, 4316 independent reflections (R_int_ = 0.042) the final *R* = 0.0512 [*I* > 2 σ(*I*), 3663 data] and *w*R(*F*^2^) = 0.1568 (all data) GOOF = 1.050. 

Crystal data for compound **8**: M = 670.83, T = 100.0(10) K, λ = 0.71073 Å, Triclinic, space group P-1 a = 8.3967(3), b = 11.2949(2), c = 11.5686(3) Å, α = 69.978(2) β = 74.635(2)° γ = 83.665(2)° V = 993.78(5) Å^3^, Z = 2, D_c_ = 2.242 Mg M^−3^ μ (Mo-Kα) = 10.121 mm^−1^, F(000) = 632, crystal size 0.51 × 0.45 × 0.32 mm^3^. θ_max_ = 29.998°, 21499 reflections measured, 5790 independent reflections (R_int_ = 0.086) the final *R* = 0.0451 [*I* > 2 σ(*I*), 4899 data] and *w*R(*F*^2^) = 0.1185 (all data) GOOF = 1.054. 

Crystal data for compound **9**: M = 670.83, T = 100.0(10) K, λ = 0.710918 Å, Orthorhombic, space group P2_1_2_1_2_1_ a = 12.741(3), b = 15.132(3), c = 20.495(43) Å, V = 3951.4(14) Å^3^, Z = 8 Z’ = 2, Dc = 2.255 Mg M^−3^ μ = 10.182 mm^−1^, F(000) = 2528, crystal size 0.10 × 0.06 × 0.05 mm^3^. θ_max_ = 32.17°, 73167 reflections measured, 12402 independent reflections (R_int_ = 0.0609) the final *R* = 0.0437 [*I* > 2 σ(*I*), 10763 data] and *w*R(*F*^2^) = 0.1213 (all data) GOOF = 1.083, absolute structure parameter 0.028(4).

### 3.6. Experimental Data for Natural Product **1** and Semisynthetic Compounds **2**–**13**

**Compound 1**: Fine white needles (crystallized from acetone, mp 168 °C, lit. mp 168–170 °C [13]); ^1^H NMR (DMSO-*d*_6_, 800 MHz) δ_H_ 10.81 (1H, br s, 1-OH), 7.90 (1H, d, *J* = 2.4 Hz, H-3′), 7.42 (1H, dd, *J* = 8.8, 2.4 Hz, H-5′), 7.41 (1H, d, *J* = 2.4 Hz, H-4), 7.17 (1H, d, *J* = 2.4 Hz, H-6), 6.47 (1H, d, *J* = 8.8 Hz, H-6′); ^13^C NMR (DMSO-*d*_6_, 200 MHz) δ_C_ 152.6 (C-1′), 152.1 (C-1), 138.4 (C-2), 135.1 (C-3′), 131.6 (C-5′), 125.1 (C-4), 119.5 (C-6), 118.7 (C-5), 118.0 (C-3), 115.9 (C-6′), 114.0 (C-4′), 111.6 (C-2′); HRESIMS *m*/*z* 520.7002 [M + Na]^+^ (calcd C_12_H_6_^79^Br_4_O_2_Na, 520.6994).

**Compound 2**: Fine white needles (10.2 mg, 99%, crystallized from acetone, mp 298 °C); UV (MeOH) λ_max_ (log ε) 280 (3.44) nm; ^1^H NMR (DMSO-*d*_6_, 800 MHz) δ_H_ 7.90 (1H, d, *J* = 2.4 Hz, H-3′), 7.58 (1H, d, *J* = 2.2 Hz, H-4), 7.47 (1H, d, *J* = 2.2 Hz, H-6), 7.40 (1H, dd, *J* = 8.8, 2.4 Hz, H-5′), 6.45 (1H, d, *J* = 8.8 Hz, H-6′), 3.77 (3H, s, H-1″); ^13^C NMR (DMSO-*d*_6_, 200 MHz) δ_C_ 153.5 (C-1), 152.5 (C-1′), 139.0 (C-2), 135.2 (C-3′), 131.7 (C-5′), 126.6 (C-4), 119.5 (C-5), 117.8 (C-3), 116.5 (C-6), 115.8 (C-6′), 114.2 (C-4′), 111.5 (C-2′), 56.9 (C-1″); HRESIMS *m*/*z* 550.6893 [M + K]^+^ (calcd C_13_H_8_^79^Br_4_O_2_K, 550.6889).

**Compound 3**: Off-white amorphous solid (9.2 mg, 81%); UV (MeOH) λ_max_ (log ε) 283 (3.22) nm; ^1^H NMR (DMSO-*d*_6_, 800 MHz) δ_H_ 7.92 (1H, d, *J* = 2.4 Hz, H-3′), 7.60 (1H, d, *J* = 2.2 Hz, H-4), 7.46 (1H, d, *J* = 2.2 Hz, H-6), 7.41 (1H, dd, *J* = 8.8, 2.4 Hz, H-5′), 6.49 (1H, d, *J* = 8.8 Hz, H-6′), 5.81 (1H, m, H-2″), 5.14 (1H, m, H-3″a), 5.13 (1H, m, H-3″b), 4.60 (2H, d, *J* = 4.8 Hz, H-1″); ^13^C NMR (DMSO-*d*_6_, 200 MHz); δ_C_ 152.6 (C-1′), 152.1 (C-1), 139.5 (C-2), 135.1 (C-3′), 132.4 (C-2″), 131.7 (C-5′), 126.8 (C-4), 119.2 (C-5), 117.9 (C-3), 117.6 (C-6), 117.2 (C-3″), 116.0 (C-6′), 114.2 (C-4′), 111.5 (C-2′), 69.4 (C-1″); HRESIMS *m*/*z* 560.7306 [M + Na]^+^ (calcd C_15_H_10_^79^Br_4_O_2_Na, 560.7307).

**Compound 4:** Off-white amorphous solid (3.6 mg, 31%); UV (MeOH) λ_max_ (log ε) 283 (3.46) nm; ^1^H NMR (DMSO-*d*_6_, 800 MHz) δ_H_ 7.90 (1H, d, *J* = 2.4 Hz, H-3′), 7.58 (1H, d, *J* = 2.2 Hz, H-4), 7.51 (1H, d, *J* = 2.2 Hz, H-6), 7.40 (1H, dd, *J* = 8.8, 2.4 Hz, H-5′), 6.49 (1H, d, *J* = 8.8 Hz, H-6′), 4.74 (1H, t, *J* = 5.0 Hz, 2″-OH), 4.05 (2H, t, *J* = 5.0 Hz, H-1″), 3.50 (2H, dt, *J* = 5.0, 5.0 Hz, H-2″); ^13^C NMR (DMSO-*d*_6_, 200 MHz) δ_C_ 153.1 (C-1), 152.7 (C-1′), 139.7 (C-2), 135.1 (C-3′), 131.6 (C-5′), 126.7 (C-4), 119.2 (C-5), 117.71 (C-3), 117.68 (C-6), 116.3 (C-6′), 114.2 (C-4′), 111.8 (C-2′), 71.2 (C-1″), 59.3 (C-2″); HRESIMS *m*/*z* 580.7000 [M + Na]^+^ (calcd C_14_H_10_^79^Br_4_O_3_K, 580.6995).

**Compound 5**: Yellow oil (6.5 mg, 61%); UV (MeOH) λ_max_ (log ε) 290 (2.38) nm; ^1^H NMR (DMSO-*d*_6_, 800 MHz) δ_H_ 7.91 (1H, d, *J* = 2.4 Hz, H-3′), 7.55 (1H, d, *J* = 2.2 Hz, H-4), 7.41 (1H, dd, *J* = 8.8, 2.4 Hz, C-5′), 7.40 (1H, d, *J* = 2.2 Hz, H-6), 6.51 (1H, d, *J* = 8.8 Hz, H-6′), 4.90 (1H, br t, *J* = 3.2 Hz, H-1″), 1.71 (4H, br s, H-2″, H-5″), 1.42 (4H, br s, H-3″, H-4″); ^13^C NMR (DMSO-*d*_6_, 200 MHz) δ_C_ 153.0 (C-1′), 151.1 (C-1), 140.3 (C-2), 135.0 (C-3′), 131.6 (C-5′), 126.1 (C-4), 118.9 (C-5), 117.78 (C-3), 117.77 (C-6), 116.5 (C-6′), 114.3 (C-4′), 111.9 (C-2′), 80.9 (C-1″), 31.9 (C-2″, C-5″), 23.0 (C-3″, C-4″); HRESIMS *m*/*z* 588.7618 [M + Na]^+^ (calcd C_17_H_14_^79^Br_4_O_2_Na, 588.7620).

**Compound 6**: Yellow amorphous solid (7.6 mg, 62%); UV (MeOH) λ_max_ (log ε) 283 (3.42) nm; ^1^H NMR (DMSO-*d*_6_, 800 MHz) δ_H_ 7.88 (1H, d, *J* = 2.4 Hz, H-3′), 7.61 (1H, d, *J* = 2.2 Hz, H-4), 7.56 (1H, d, *J* = 2.2 Hz, H-6), 7.41 (1H, dd, *J* = 8.8, 2.4 Hz, H-5′), 7.27 (3H, m, H-4″, H-5″, H-6″), 7.10 (2H, m, H-3″, H-7″), 6.53 (1H, d, *J* = 8.8 Hz, H-6′), 5.13 (2H, s, H-1″); ^13^C NMR (DMSO-*d*_6_, 200 MHz) δ_C_ 152.8 (C-1′), 152.1 (C-1), 139.8 (C-2), 135.8 (C-2″), 135.1 (C-3′), 131.7 (C-5′), 128.3 (C-4″, C-6″), 128.0 (C-5″), 127.1 (C-3″, C-7″), 126.9 (C-4), 119.1 (C-5), 117.9 (C-3), 117.7 (C-6), 116.3 (C-6′), 114.4 (C-4′), 111.8 (C-2′), 70.5 (C-1″); HRESIMS *m*/*z* 626.7214 [M + K]^+^ (calcd C_19_H_12_^79^Br_4_O_2_K, 626.7202).

**Compound 7**: Clear ultra-fine needles (10.9 mg, 88%, crystallized from CH_3_CN, mp 78 °C); UV (MeOH) λ_max_ (log ε) 282 (2.78) nm; ^1^H NMR (DMSO-*d*_6_, 800 MHz) δ_H_ 7.89 (1H, d, *J* = 2.4 Hz, H-3′), 7.60 (1H, d, *J* = 2.2 Hz, H-4), 7.56 (1H, d, *J* = 2.2 Hz, H-6), 7.41 (1H, dd, *J* = 8.8, 2.4 Hz, H-5′), 7.08 (2H, d, *J* = 7.8 Hz, H-4″, H-7″), 6.98 (2H, d, *J* = 7.8 Hz, H-3″, H-8″), 6.51 (1H, d, *J* = 8.8 Hz, H-6′), 5.08 (2H, s, H-1″), 2.27 (3H, s, H-6″); ^13^C NMR (DMSO-*d*_6_, 200 MHz) δ_C_ 152.7 (C-1′), 152.1 (C-1), 139.8 (C-2), 137.3 (C-5″), 135.1 (C-3′), 132.7 (C-2″), 131.7 (C-5′), 128.8 (C-4″, C-7″), 127.2 (C-3″, C-8″), 126.8 (C-4), 119.1 (C-5), 117.8 (C-3), 117.8 (C-6), 116.2 (C-6′), 114.3 (C-4′), 111.7 (C-2′), 70.5 (C-1″), 20.8 (C-6″); HRESIMS *m*/*z* 624.7621 [M + Na]^+^ (calcd C_20_H_14_^79^Br_4_O_2_Na, 624.7620).

**Compound 8**: White needles (8.5 mg, 61%, crystallized from acetone, mp 294 °C); UV (MeOH) λ_max_ (log ε) 282 (2.13) nm; ^1^H NMR (DMSO-*d*_6_, 800 MHz) δ_H_ 7.90 (1H, d, *J* = 2.4 Hz, H-3′), 7.63 (1H, d, *J* = 2.1 Hz, H-4), 7.56 (1H, d, *J* = 2.1 Hz, H-6), 7.48 (2H, m, H-4″, H-6″), 7.41 (1H, dd, *J* = 8.8, 2.4 Hz, H-5′), 7.05 (2H, m, H-3″, H-7″), 6.53 (1H, d, *J* = 8.8 Hz, H-6′), 5.12 (2H, s, H-1″); ^13^C NMR (DMSO-*d*_6_, 200 MHz) δ_C_ 152.7 (C-1′), 151.9 (C-1), 139.8 (C-2), 135.2 (C-2″), 135.2 (C-3′), 131.7 (C-5′), 131.2 (C-4″, C-6″), 129.3 (C-3″, C-7″), 127.1 (C-4), 121.2 (C-5″), 119.1 (C-5), 117.9 (C-3), 117.8 (C-6), 116.3 (C-6′), 114.4 (C-4′), 111.8 (C-2′), 69.8 (C-1″); HRESIMS *m*/*z* 688.6566 [M + Na]^+^ (calcd C_19_H_11_^79^Br_5_O_2_Na, 688.6568).

**Compound 9**: Fine white needles (10.1 mg, 81%, crystallized from acetone, mp 98 °C); UV (MeOH) λ_max_ (log ε) 282 (3.05) nm; ^1^H NMR (DMSO-*d*_6_, 800 MHz) δ_H_ 7.90 (1H, d, *J* = 2.4 Hz, H-3′), 7.64 (1H, d, *J* = 2.2 Hz, H-4), 7.57 (1H, d, *J* = 2.2 Hz, H-6), 7.47 (1H, ddd, *J* = 7.8, 1.7, 0.9 Hz, H-5″), 7.41 (1H, dd, *J* = 8.8, 2.4 Hz, H-5′), 7.25 (1H, dd, *J* = 7.8, 7.8 Hz, H-6″), 7.15 (1H, dd, *J* = 1.7, 1.7 Hz, H-3″), 7.13 (1H, ddd, *J* = 7.8, 1.7, 0.9 Hz, H-7″), 6.55 (1H, d, *J* = 8.8 Hz, H-6′), 5.14 (2H, s, H-1″); ^13^C NMR (DMSO-*d*_6_, 200 MHz) δ_C_ 152.7 (C-1′), 151.7 (C-1), 139.7 (C-2), 138.5 (C-2″), 135.3 (C-3′), 131.7 (C-5′), 130.8 (C-5″), 130.4 (C-6″), 129.4 (C-7″), 127.1 (C-4), 125.9 (C-3″), 121.7 (C-4″), 119.1 (C-5), 117.9 (C-3), 117.6 (C-6), 116.1 (C-6′), 114.5 (C-4′), 111.6 (C-2′), 69.4 (C-1″); HRESIMS *m*/*z* 688.6569 [M + Na]^+^ (calcd C_19_H_11_^79^Br_5_O_2_Na, 688.6568).

**Compound 10**: Off-white oil (7.0 mg, 55%); UV (MeOH) λ_max_ (log ε) 283 (2.37) nm; ^1^H NMR (DMSO-*d*_6_, 800 MHz) δ_H_ 7.90 (1H, d, *J* = 2.3 Hz, H-3′), 7.65 (1H, d, *J* = 2.2 Hz, H-4), 7.57 (1H, d, *J* = 2.2 Hz, H-6), 7.41 (1H, dd, *J* = 8.8, 2.3 Hz, H-5′), 7.34 (1H, br d, *J* = 7.6 Hz, H-5″), 7.32 (1H, dd, *J* = 7.6, 7.6 Hz, H-6″), 7.09 (1H, br d, *J* = 7.6 Hz, H-7″), 6.99 (1H, br s, H-3″), 6.56 (1H, d, *J* = 8.8 Hz, H-6′), 5.14 (2H, s, H-1″); ^13^C NMR (DMSO-*d*_6_, 200 MHz) δ_C_ 152.7 (C-1′), 151.7 (C-1), 139.7 (C-2), 138.3 (C-2″), 135.3 (C-3′), 133.2 (C-4″), 131.7 (C-5′), 130.1 (C-6″), 127.9 (C-5″), 127.1 (C-4), 126.6 (C-3″), 125.5 (C-7″), 119.1 (C-5), 117.9 (C-3), 117.6 (C-6), 116.2 (C-6′), 114.5 (C-4′), 111.6 (C-2′), 69.5 (C-1″); HRESIMS *m*/*z* 644.7073 [M + Na]^+^ (calcd C_19_H_11_^79^Br_4_ClO_2_Na, 644.7073).

**Compound 11**: Off-white oil (13.3 mg, 99%); UV (MeOH) λ_max_ (log ε) 280 (3.52) nm; ^1^H NMR (DMSO-*d*_6_, 800 MHz) δ_H_ 8.15 (1H, br d, *J* = 7.9 Hz, H-5″), 7.95 (1H, br s, H-3″), 7.85 (1H, d, *J* = 2.4 Hz, H-3′), 7.66 (1H, d, *J* = 2.1 Hz, H-4), 7.61 (1H, d, *J* = 2.1 Hz, H-6), 7.60 (1H, dd, *J* = 7.9, 7.9 Hz, H-6″), 7.56 (1H, br d, *J* = 7.9 Hz, H-7″), 7.38 (1H, dd, *J* = 8.8, 2.4 Hz, H-5′), 6.56 (1H, d, *J* = 8.8 Hz, H-6′), 5.29 (2H, s, H-1″); ^13^C NMR (DMSO-*d*_6_, 200 MHz) δ_C_ 152.6 (C-1′), 151.7 (C-1), 147.7 (C-4″), 138.8 (C-2), 138.1 (C-2″), 135.2 (C-3′), 133.5 (C-7″), 131.7 (C-5′), 128.9 (C-6″), 127.3 (C-4), 122.9 (C-5″), 121.6 (C-3″), 119.1 (C-5), 118.0 (C-3), 117.8 (C-6), 116.2 (C-6′), 114.4 (C-4′), 111.6 (C-2′), 69.3 (C-1″); HRESIMS *m*/*z* 655.7318 [M + Na]^+^ (calcd C_19_H_11_^79^Br_4_NO_4_Na, 655.7314).

**Compound 12**: White ultra-fine needles (9.2 mg, 73%, crystallized from acetone, mp 164 °C); UV (MeOH) λ_max_ (log ε) 272 (3.29) nm; ^1^H NMR (DMSO-*d*_6_, 800 MHz) δ_H_ 7.84 (1H, d, *J* = 2.4 Hz, H-3′), 7.67 (1H, m, H-6), 7.66 (1H, m, H-4), 7.38 (1H, dd, *J* = 8.8, 2.4 Hz, H-5′), 7.22 (1H, m, H-4″), 7.20 (1H, m, H-5″), 6.84 (1H, ddd, *J* = 8.7, 5.5, 3.1 Hz, H-7″), 6.53 (1H, d, *J* = 8.8 Hz, H-6′), 5.17 (2H, s, H-1″); ^13^C NMR (DMSO-*d*_6_, 200 MHz) δ_C_ 157.4 (d, *J* = 240.1 Hz, C-6″), 155.8 (d, *J* = 240.1 Hz, C-3″), 152.7 (C-1′), 151.6 (C-1), 139.8 (C-2), 135.1 (C-3′), 131.6 (C-5′), 127.3 (C-4), 124.7 (dd, *J* = 17.4, 8.0 Hz, C-2″), 119.1 (C-5), 117.9 (C-6), 117.8 (C-3), 116.9 (dd, *J* = 23.9, 8.7 Hz, C-4″), 116.6 (dd, *J* = 23.9, 8.7 Hz, C-5″), 116.3 (C-6′), 115.6 (dd, *J* = 25.1, 4.3 Hz, C-7″), 114.5 (C-4′), 111.6 (C-2′), 64.4 (C-1″); HRESIMS *m*/*z* 646.7273 [M + Na]^+^ (calcd C_19_H_10_^79^Br_4_F_2_O_2_Na, 646.7275). 

**Compound 13**: Off-white amorphous solid (2.1 mg, 17%); UV (MeOH) λ_max_ (log ε) 279 (3.83) nm; ^1^H NMR (DMSO-*d*_6_, 800 MHz) δ_H_ 7.83 (1H, d, *J* = 2.4 Hz, H-3′), 7.59 (1H, d, *J* = 2.2 Hz, H-4), 7.44 (1H, dd, *J* = 8.8, 2.4 Hz, H-5′), 7.41 (1H, ddd, *J* = 7.6, 7.6, 1.4 Hz, H-11″), 7.39 (2H, m, H-6″, H-8″), 7.37 (2H, m, H-5″, H-9″), 7.33 (1H, d *J* = 2.2 Hz, H-6), 7.31 (1H, td, *J* = 7.6, 1.4 Hz, H-7″), 7.27 (1H, dd, *J* = 7.6, 1.4 Hz, H-10″), 7.23 (2H, m, H-12″, H-13″), 6.51 (1H, d, *J* = 8.8 Hz, H-6′), 4.94 (2H, s, H-1″); ^13^C NMR (DMSO-*d*_6_, 200 MHz) δ_C_ 152.8 (C-1′), 152.1 (C-1), 141.7 (C-3″), 139.7 (C-2), 139.6 (C-4″), 135.1 (C-3′), 132.4 (C-5″, C-9″), 131.7 (C-5′), 129.8 (C-10″), 129.7 (C-2″), 128.9 (C-13″), 128.6 (C-11″, C-12″), 128.3 (C-6″, C-8″), 127.4 (C-7″), 126.9 (C-4), 119.1 (C-5), 117.9 (C-3), 117.3 (C-6), 116.3 (C-6′), 114.4 (C-4′), 111.8 (C-2′), 69.2 (C-1″); HRESIMS *m*/*z* 686.7777 [M + Na]^+^ (calcd C_25_H_16_^79^Br_4_O_2_Na, 686.7776).

### 3.7. Cancer Cell Cytotoxicity Assays

Evaluation of compound cytotoxicity was performed as previously described, with minor modifications [29,30]. MCF-7 and MDA-MB-231 cells were cultured in DMEM media supplemented with 10% heat-inactivated FBS (2500 cells and 2000 cells/50 µL/well seeded, respectively). DU145 cells were cultured in DMEM media supplemented with 10% heat-inactivated FBS (1000 cell/50 µL/well seeded). LNCaP cells were cultured in RPMI media supplemented with 10% heat-inactivated FBS (2000 cells/well seeded). Compounds (prepared at 20 mM stock concentration in DMSO) were evaluated using a 11-point assay concentration range from 0 to 50 µM. Assay controls included 0.4% DMSO negative control) and either 10% DMSO or 50 µM puromycin (final assay concentrations) (positive controls). Compounds were added 24 h after cell seeding into Greiner black-wall, clear-bottom 384-well cell culture plates and incubated for 72 h. After 66 h, resazurin was added to a final concentration of 60 µM, and samples were incubated for another 6 h. Fluorescence was monitored (excitation and emission wavelengths, 530 and 590 nm, respectively) using a PerkinElmer EnSpire plate reader at 72 h. Data for MCF-7 and LNCaP cells were normalized to 0.4% DMSO and 10% DMSO, whereas MDA-MB-231 and DU145 data were normalized to 0.4% DMSO and 50 µM puromycin controls.

## Figures and Tables

**Table 1:** NMR data of compound **8** in DMSO-*d*_6_^a^.

Position	^1^H mult. (*J* in Hz, int.)	^13^C, mult.	COSY	HMBC	ROESY
1	-	151.9, C	-	-	-
2	-	139.8, C	-	-	-
3	-	117.9, C	-	-	-
4	7.63 d (2.1, 1H)	127.1, CH	6	1 ^w^, 2, 5, 6	-
5	-	119.1, C	-	-	-
6	7.56 d (2.1, 1H)	117.8, CH	4	1, 2, 4, 5	1″
1′	-	152.7, C	-	-	-
2′	-	111.8, C	-	-	-
3′	7.90 d (2.4, 1H)	135.2, CH	5′	1′, 2′, 4′, 5′	-
4′	-	114.4, C	-	-	-
5′	7.41 dd (8.8, 2.4, 1H)	131.7, CH	3′, 6′	1′, 2′ ^w^, 3′, 4′	6′
6′	6.53 d (8.8, 1H)	116.3, CH	5′	1′, 2′, 3′ ^w^, 4′	5′
1″	5.12 s (2H)	69.8, CH_2_	-	1, 2″ ^w^, 3″, 7″	6, 3″, 7″
2″	-	135.2, C	-	-	-
3″	7.05 m (1H)	129.3, CH	4″	1″, 5″, 7″	1″, 4″
4″	7.48 m (1H)	131.2, CH	3″	2″, 3″, 5″, 6″, 7″ ^w^	3″
5″	-	121.2, C	-	-	-
6″	7.48 m (1H)	131.2, CH	7″	2″, 3″ ^w^, 4″, 5″, 7″	7″
7″	7.05 m (1H)	129.3, CH	6″	1″, 3″, 5″	1″, 6″

^a^ Spectra recorded at 25 °C (800 MHz for ^1^H NMR and 200 MHz for ^13^C NMR); ^w^ weak correlation.

**Table 2:** Cytotoxic data for compounds **1**–**13**.

Compound	Average % Inhibition at 50 µM ± SD			
	DU145	LNCaP	MCF-7	MDA-MB-231
**1**	39 ± 5	63 ± 1	63 ± 0	65 ± 10
**2**	IA ^a^	IA	IA	IA
**3**	21 ± 1	22 ± 9	64 ± 1	55 ± 4
**4**	IA	IA	IA	IA
**5**	11 ± 1	4 ± 0	55 ± 1	17 ± 2
**6**	IA	IA	IA	IA
**7**	IA	IA	IA	IA
**8**	IA	IA	1 ± 1	IA
**9**	13 ± 4	9 ± 1	62 ± 4	8 ± 11
**10**	14 ± 3	3 ± 1	47 ± 0	7 ± 10
**11**	IA	13 ± 1	26 ± 2	IA
**12**	IA	2 ± 1	IA	IA
**13**	IA	IA	IA	IA
Puromycin IC_50_ (µM)	0.24 ± 0.01	0.20 ± 0.00	0.21 ± 0.08	0.15 ± 0.02

^a^ IA = inactive at 50 µM.

## Data Availability

The data presented in this study are available in the article.

## Supplementary Materials

The following supporting information can be downloaded at https://www.mdpi.com/article/10.3390/md22010033/s1, Figure S1: UHPLCLC-MS trace of *Lamellodysidea* sp. OTU 2054 extract; Figure S2: Alkyl halides used to generate compounds **2**–**13**; Figures S3–S80: NMR spectra of compounds **1**–**13**; Figures S81–S93: HRESIMS data for compounds **1**–**13**.
