# Supplementary material for: Semisynthesis and Cytotoxic Evaluation of an Ether Analogue Library Based on a Polyhalogenated Diphenyl Ether Scaffold Isolated from a Lamellodysidea Sponge
Source: Mar Drugs. 2024 Jan 3;22(1):33. doi: 10.3390/md22010033 (PMC10817568; doi:10.3390/md22010033)
Supplement: Supplementary file 1 [file marinedrugs-22-00033-s001.zip › marinedrugs-2781001-supplementary.pdf]

Semisynthesis and Cytotoxic Evaluation of an Ether Analogue Library Based on a Polyhalogenated Diphenyl Ether Scaffold Isolated from a *Lamellodysidea* Sponge

Kelsey S. Ramage [1], Aaron Lock [2], Jonathan M. White [3], Merrick G. Ekins [1,4], Milton J. Kiefel [5], Vicky M. Avery [2] and Rohan A. Davis [1,6,*]

1 Griffith Institute for Drug Discovery, School of Environment and Science, Griffith University, Brisbane, QLD 4111, Australia
2 Discovery Biology, School of Environment and Science, Griffith University, Brisbane, QLD 4111, Australia
3 School of Chemistry and Bio21 Institute, The University of Melbourne, Parkville, VIC 3010, Australia
4 Queensland Museum, South Brisbane, QLD 4101, Australia
5 Institute for Glycomics, School of Environment and Science, Griffith University, Gold Coast, QLD 4222, Australia
6 NatureBank, Griffith Institute for Drug Discovery, Griffith University, Nathan, QLD 4111, Australia

Contents

S1: UHPLC-MS Trace of *Lamellodysidea* sp. OTU 2054 CH_2Cl_2 Extract 5

S2: Alkyl Halides (R-X) Used to Generate Compounds **2–13**. 5

S3: ^{1}H NMR (800 MHz) Spectrum of Compound **1** in DMSO-d_6 6

S4: ^{13}C NMR (200 MHz) Spectrum of Compound **1** in DMSO-d_6 6

S5: COSY Spectrum of Compound **1** in DMSO-d_6 7

S6: HSQC Spectrum of Compound **1** in DMSO-d_6 7

S7: HMBC Spectrum of Compound **1** in DMSO-d_6 8

S8: ROESY Spectrum of Compound **1** in DMSO-d_6 8

S9: ^{1}H NMR (800 MHz) Spectrum of Compound **2** in DMSO-d_6 9

S10: ^{13}C NMR (200 MHz) Spectrum of Compound **2** in DMSO-d_6 9

S11: COSY Spectrum of Compound **2** in DMSO-d_6 10

S12: HSQC Spectrum of Compound **2** in DMSO-d_6 10

S13: HMBC Spectrum of Compound **2** in DMSO-d_6 11

S14: ROESY Spectrum of Compound **2** in DMSO-d_6 11

S15: ^{1}H NMR (800 MHz) Spectrum of Compound **3** in DMSO-d_6 12

S16: ^{13}C NMR (200 MHz) Spectrum of Compound **3** in DMSO-d_6 12

S17: COSY Spectrum of Compound **3** in DMSO-d_6 13

S18: HSQC Spectrum of Compound **3** in DMSO-d_6 13

S19: HMBC Spectrum of Compound **3** in DMSO-d_6 14

S20: ROESY Spectrum of Compound **3** in DMSO-d_6 14

S21: ^{1}H NMR (800 MHz) Spectrum of Compound **4** in DMSO-d_6 15

S22: ^{13}C NMR (200 MHz) Spectrum of Compound **4** in DMSO-d_6 15

S23: COSY Spectrum of Compound **4** in DMSO-d_6 16

S24: HSQC Spectrum of Compound **4** in DMSO-d_6 16

S25: HMBC Spectrum of Compound **4** in DMSO-d_6 17

S26: ROESY Spectrum of Compound **4** in DMSO-d_6 17

S27: ^{1}H NMR (800 MHz) Spectrum of Compound **5** in DMSO-d_6 18

S28: ^{13}C NMR (200 MHz) Spectrum of Compound **5** in DMSO-d_6 18

S29: COSY Spectrum of Compound **5** in DMSO-d_6 19

S30: HSQC Spectrum of Compound **5** in DMSO-d_6 19

S31: HMBC Spectrum of Compound **5** in DMSO-d_6 20

S32: ROESY Spectrum of Compound **5** in DMSO-d_6 20

S33: ^{1}H NMR (800 MHz) Spectrum of Compound **6** in DMSO-d_6 21

S34: ^{13}C NMR (200 MHz) Spectrum of Compound **6** in DMSO-d_6 21

S35: COSY Spectrum of Compound **6** in DMSO-d_6 22

S36: HSQC Spectrum of Compound **6** in DMSO-d_6 22

S37: HMBC Spectrum of Compound **6** in DMSO-d_6 23
S38: ROESY Spectrum of Compound **6** in DMSO-d_6 23
S39: ^{1}H NMR (800 MHz) Spectrum of Compound **7** in DMSO-d_6 24
S40: ^{13}C NMR (200 MHz) Spectrum of Compound **7** in DMSO-d_6 24
S41: COSY Spectrum of Compound **7** in DMSO-d_6 25
S42: HSQC Spectrum of Compound **7** in DMSO-d_6 25
S43: HMBC Spectrum of Compound **7** in DMSO-d_6 26
S44: ROESY Spectrum of Compound **7** in DMSO-d_6 26
S45: ^{1}H NMR (800 MHz) Spectrum of Compound **8** in DMSO-d_6 27
S46: ^{13}C NMR (200 MHz) Spectrum of Compound **8** in DMSO-d_6 27
S47: COSY Spectrum of Compound **8** in DMSO-d_6 28
S48: HSQC Spectrum of Compound **8** in DMSO-d_6 28
S49: HMBC Spectrum of Compound **8** in DMSO-d_6 29
S50: ROESY Spectrum of Compound **8** in DMSO-d_6 29
S51: ^{1}H NMR (800 MHz) Spectrum of Compound **9** in DMSO-d_6 30
S52: ^{13}C NMR (200 MHz) Spectrum of Compound **9** in DMSO-d_6 30
S53: COSY Spectrum of Compound **9** in DMSO-d_6 31
S54: HSQC Spectrum of Compound **9** in DMSO-d_6 31
S55: HMBC Spectrum of Compound **9** in DMSO-d_6 32
S56: ROESY Spectrum of Compound **9** in DMSO-d_6 32
S57: ^{1}H NMR (800 MHz) Spectrum of Compound **10** in DMSO-d_6 33
S58: ^{13}C NMR (200 MHz) Spectrum of Compound **10** in DMSO-d_6 33
S59: COSY Spectrum of Compound **10** in DMSO-d_6 34
S60: HSQC Spectrum of Compound **10** in DMSO-d_6 34
S61: HMBC Spectrum of Compound **10** in DMSO-d_6 35
S62: ROESY Spectrum of Compound **10** in DMSO-d_6 35
S63: ^{1}H NMR (800 MHz) Spectrum of Compound **11** in DMSO-d_6 36
S64: ^{13}C NMR (200 MHz) Spectrum of Compound **11** in DMSO-d_6 36
S65: COSY Spectrum of Compound **11** in DMSO-d_6 37
S66: HSQC Spectrum of Compound **11** in DMSO-d_6 37
S67: HMBC Spectrum of Compound **11** in DMSO-d_6 38
S68: ROESY Spectrum of Compound **11** in DMSO-d_6 38
S69: ^{1}H NMR (800 MHz) Spectrum of Compound **12** in DMSO-d_6 39
S70: ^{13}C NMR (200 MHz) Spectrum of Compound **12** in DMSO-d_6 39
S71: COSY Spectrum of Compound **12** in DMSO-d_6 40
S72: HSQC Spectrum of Compound **12** in DMSO-d_6 40
S73: HMBC Spectrum of Compound **12** in DMSO-d_6 41

S74: ROESY Spectrum of Compound **12** in DMSO-d_6 41
S75: ^{1}H NMR (800 MHz) Spectrum of Compound **13** in DMSO-d_6 42
S76: ^{13}C NMR (200 MHz) Spectrum of Compound **13** in DMSO-d_6 42
S77: COSY Spectrum of Compound **13** in DMSO-d_6 43
S78: HSQC Spectrum of Compound **13** in DMSO-d_6 43
S79: HMBC Spectrum of Compound **13** in DMSO-d_6 44
S80: ROESY Spectrum of Compound **13** in DMSO-d_6 44
S81: HRESIMS Data of Compound **1** 45
S82: HRESIMS Data of Compound **2** 45
S83: HRESIMS Data of Compound **3** 46
S84: HRESIMS Data of Compound **4** 46
S85: HRESIMS Data of Compound **5** 46
S86: HRESIMS Data of Compound **6** 47
S87: HRESIMS Data of Compound **7** 47
S88: HRESIMS Data of Compound **8** 48
S89: HRESIMS Data of Compound **9** 48
S90: HRESIMS Data of Compound **10** 48
S91: HRESIMS Data of Compound **11** 49
S92: HRESIMS Data of Compound **12** 49
S93: HRESIMS Data of Compound **13** 49

S1: UHPLC-MS Trace of *Lamellodysidea* sp. OTU 2054 CH_2Cl_2 Extract

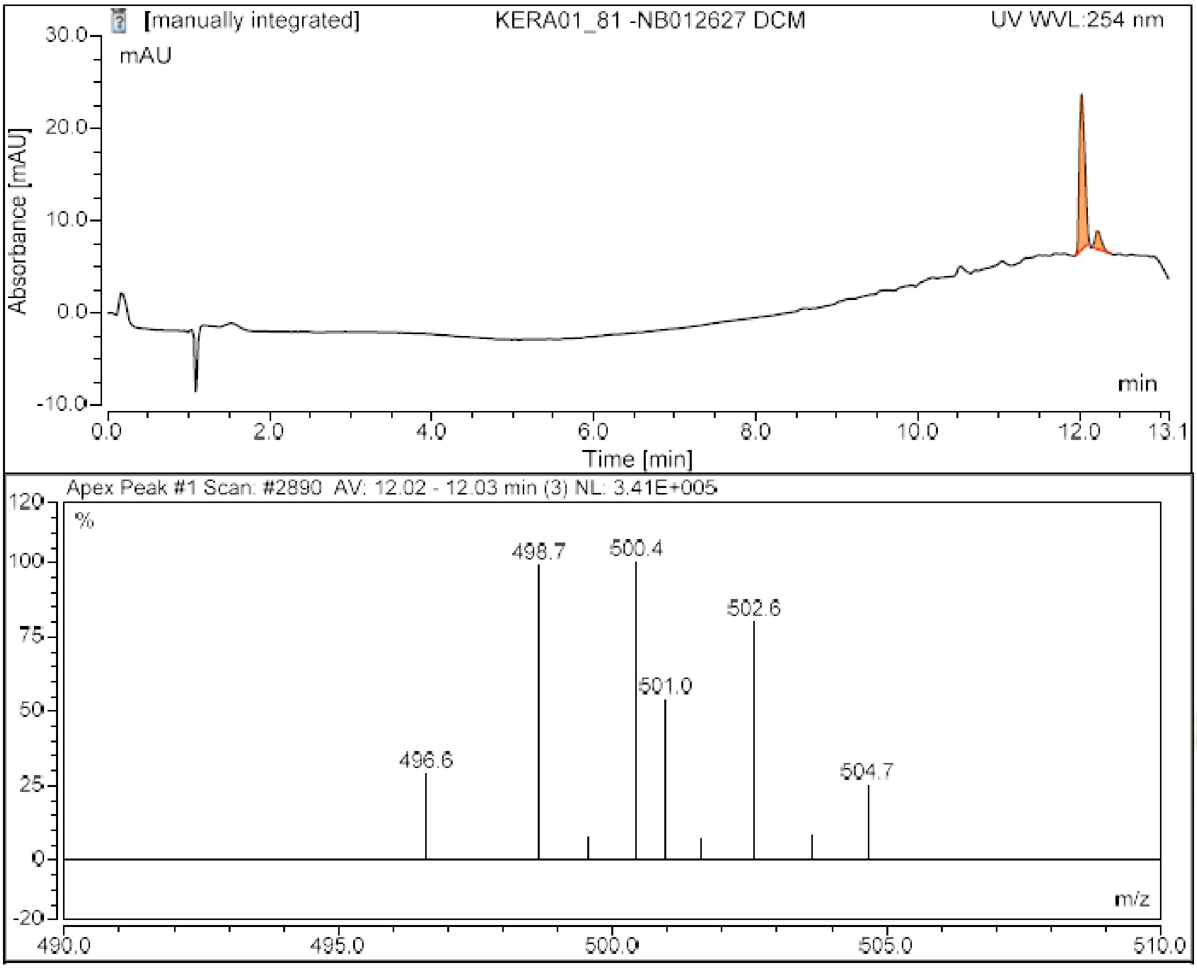

S2: Alkyl Halides (R-X) Used to Generate Compounds **2**–**13**.

Compound	R-X	Yield
**2**	methyl iodide	99%
**3**	allyl bromide	81%
**4**	bromoethanol	31%
**5**	cyclopentyl iodide	61%
**6**	benzyl bromide	62%
**7**	4-methylbenzyl bromide	88%
**8**	4-bromobenzyl bromide	61%
**9**	3-bromobenzyl bromide	81%
**10**	3-chlorobenzyl chloride	55%
**11**	3-nitrobenzyl bromide	99%
**12**	2,5-difluorobenzyl bromide	73%
**13**	2-phenylbenzyl bromide	17%

S3: ^{1}H NMR (800 MHz) Spectrum of Compound **1** in DMSO-d_6

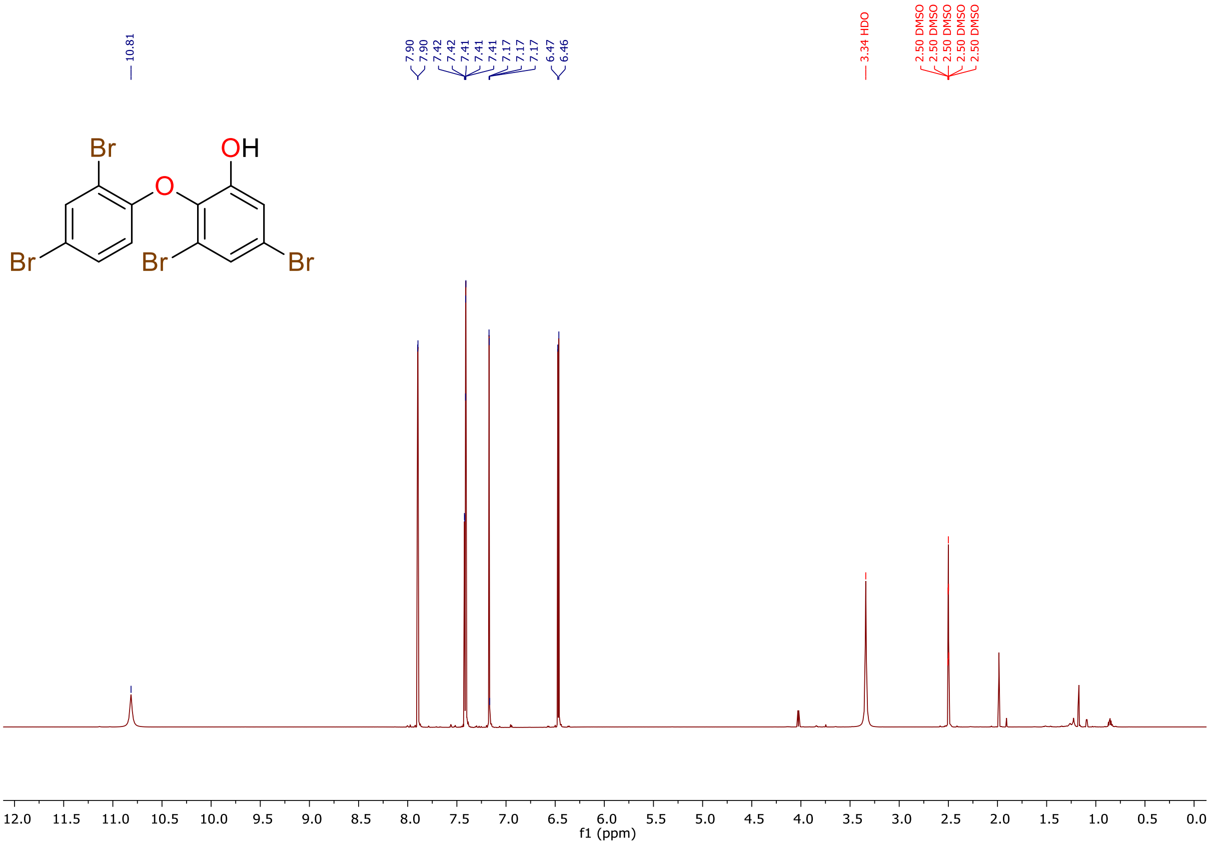

S4: ^{13}C NMR (200 MHz) Spectrum of Compound **1** in DMSO-d_6

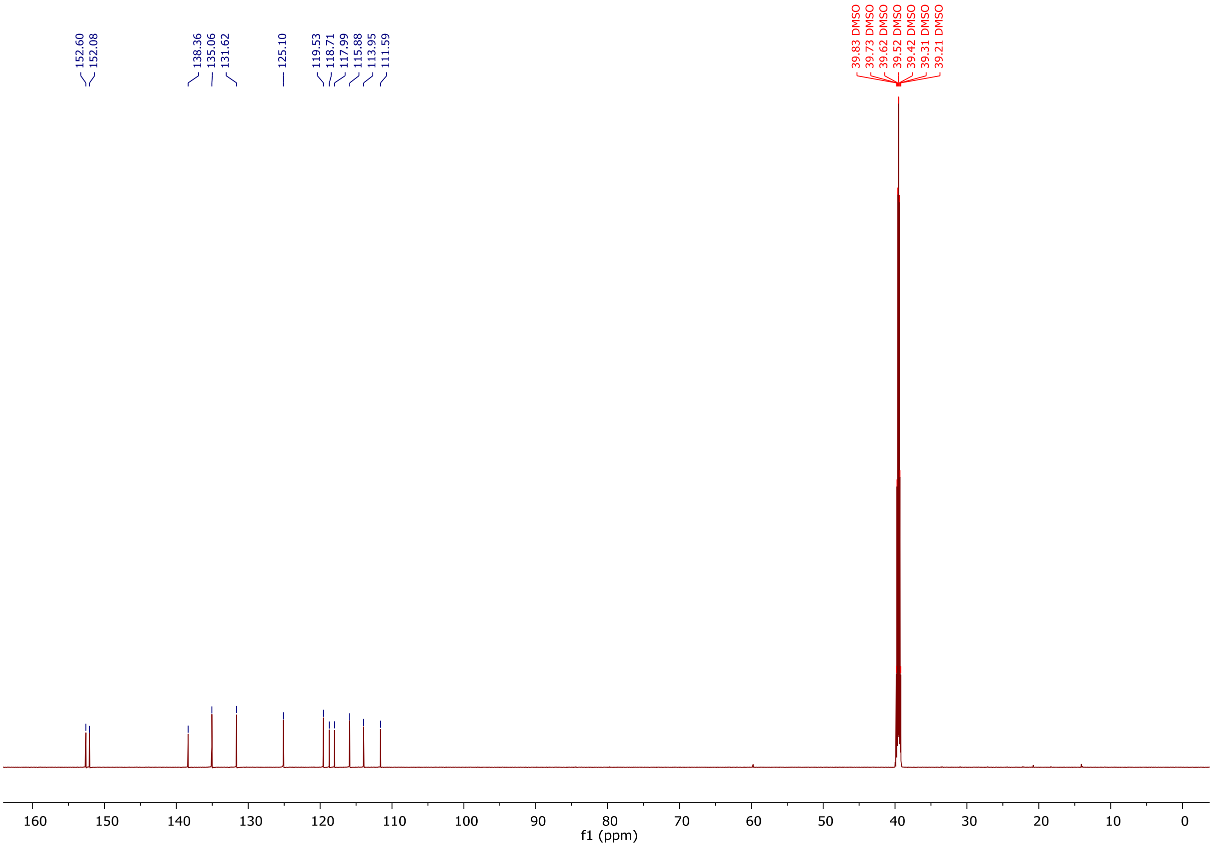

S5: COSY Spectrum of Compound **1** in DMSO-d_6

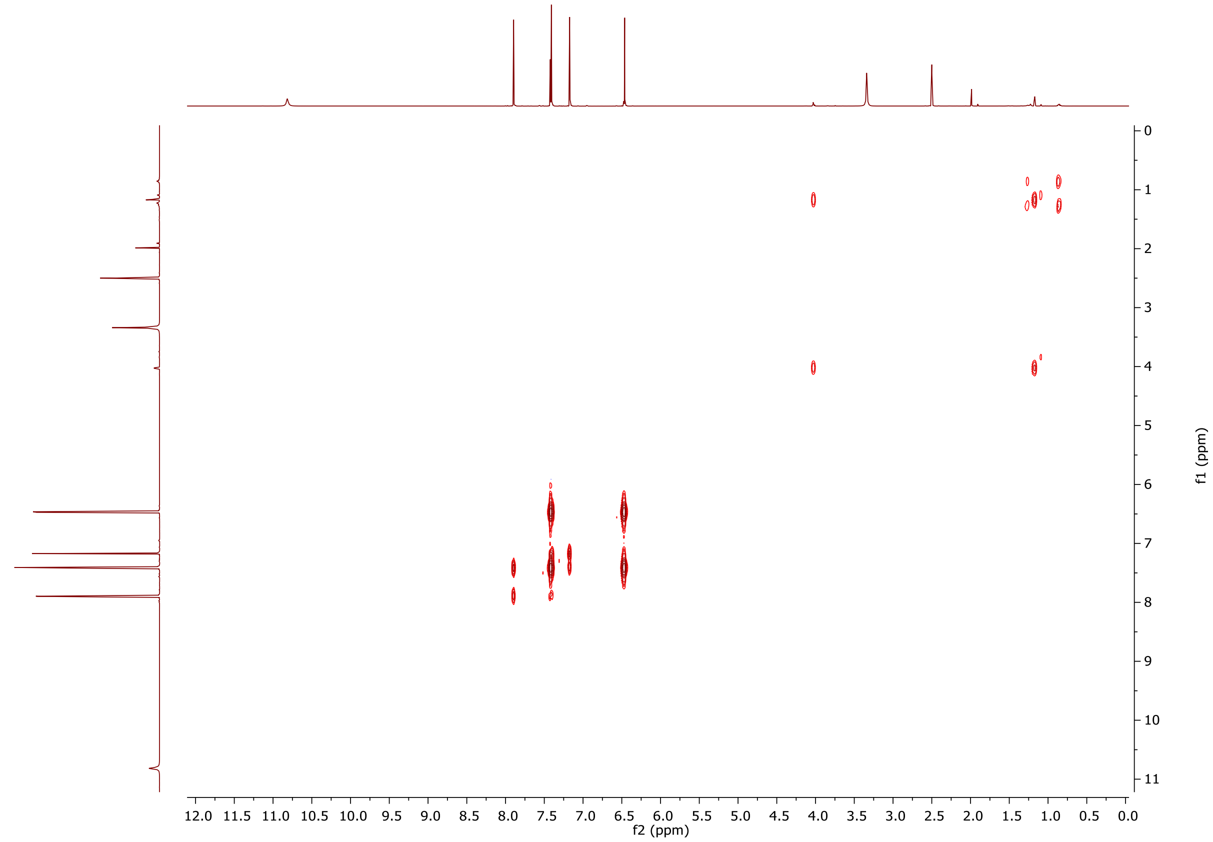

S6: HSQC Spectrum of Compound **1** in DMSO-d_6

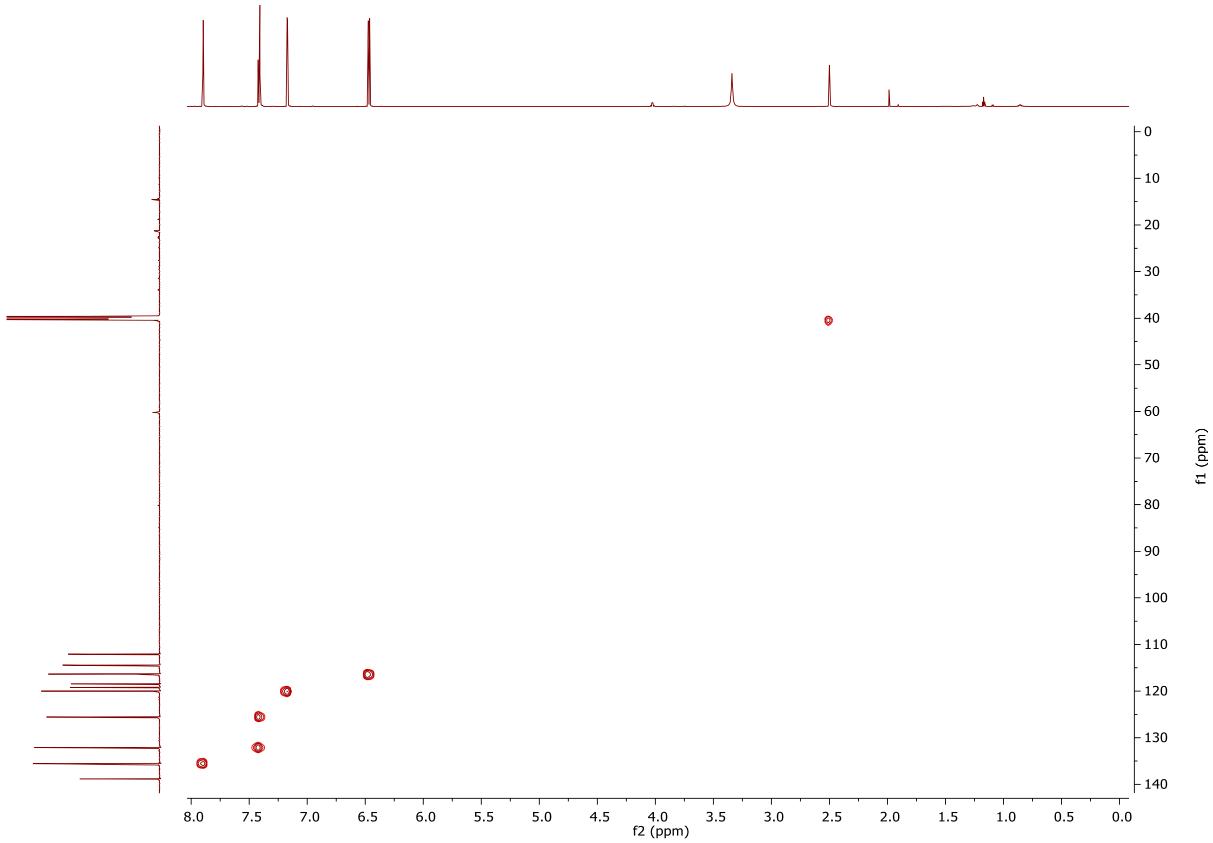

S7: HMBC Spectrum of Compound **1** in DMSO-d_6

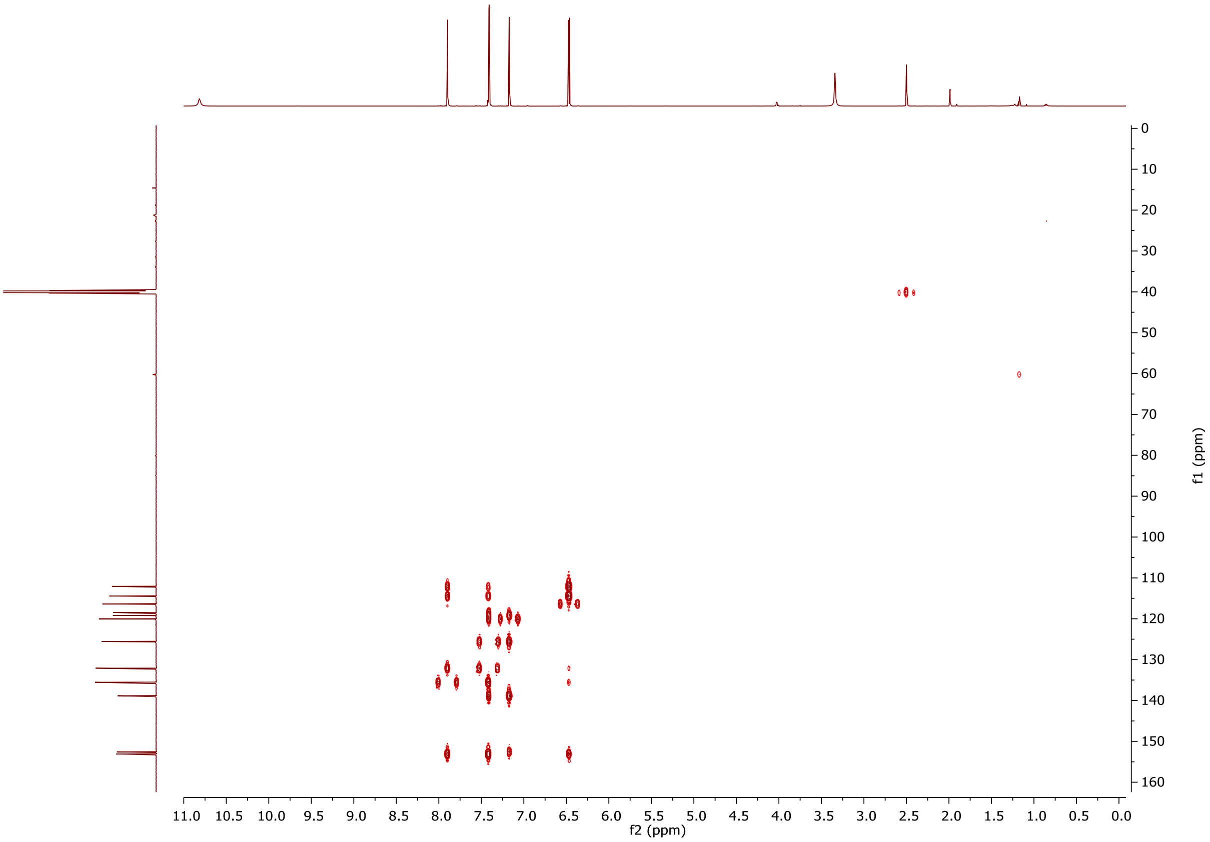

S8: ROESY Spectrum of Compound **1** in DMSO-d_6

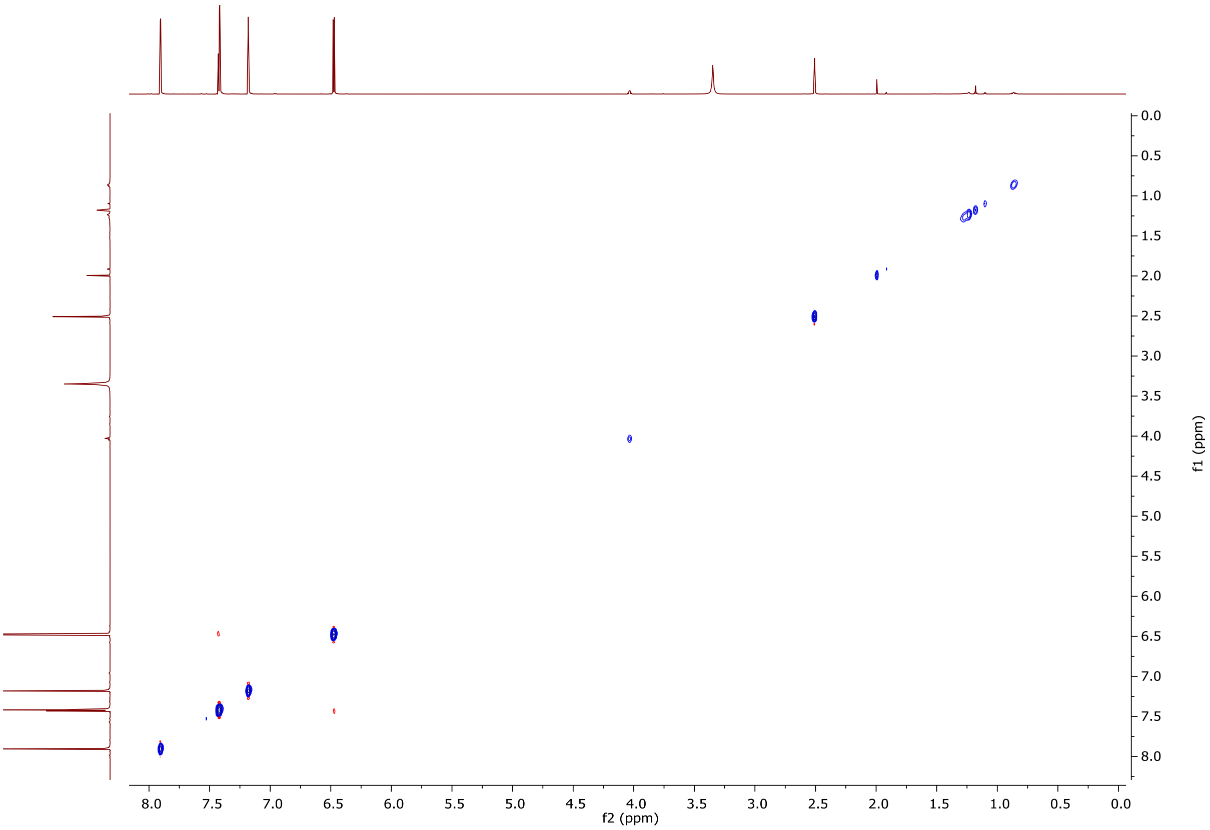

S9: ^{1}H NMR (800 MHz) Spectrum of Compound **2** in DMSO-d_6

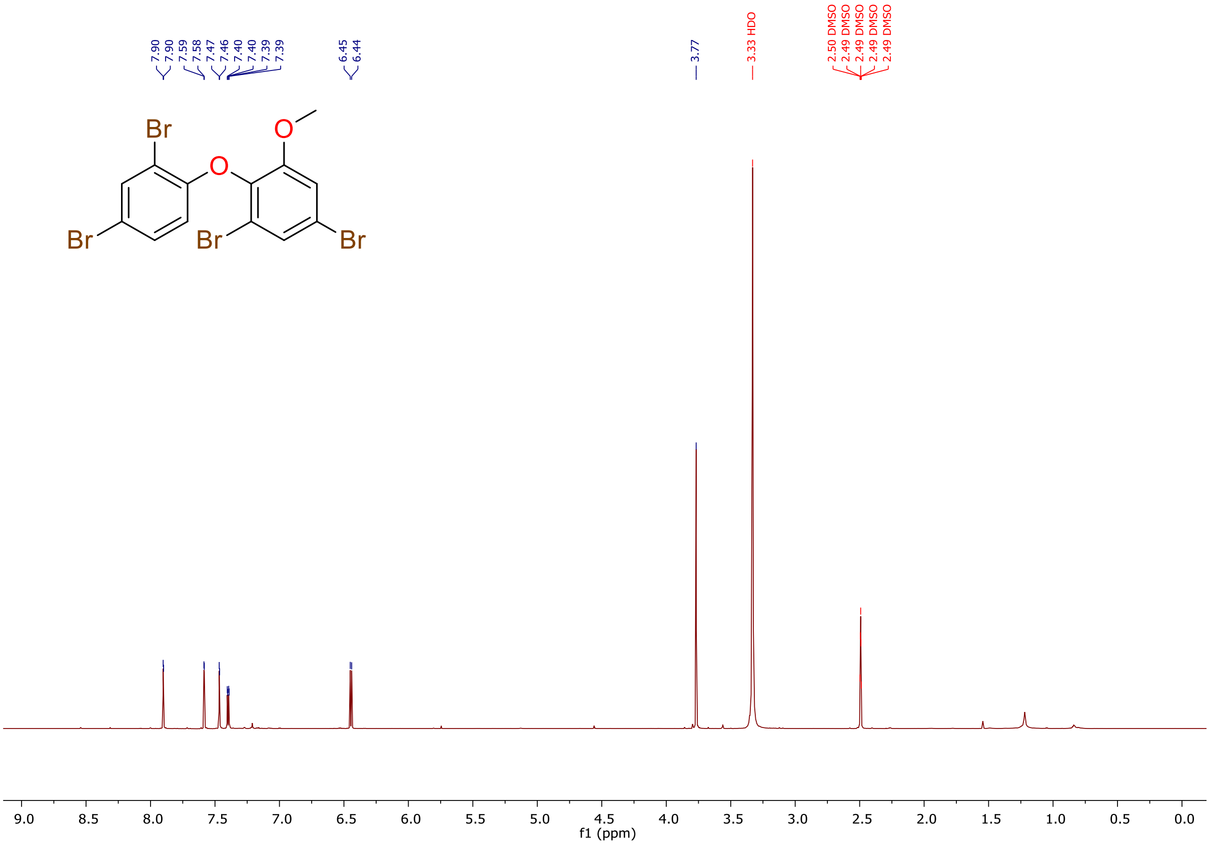

S10: ^{13}C NMR (200 MHz) Spectrum of Compound **2** in DMSO-d_6

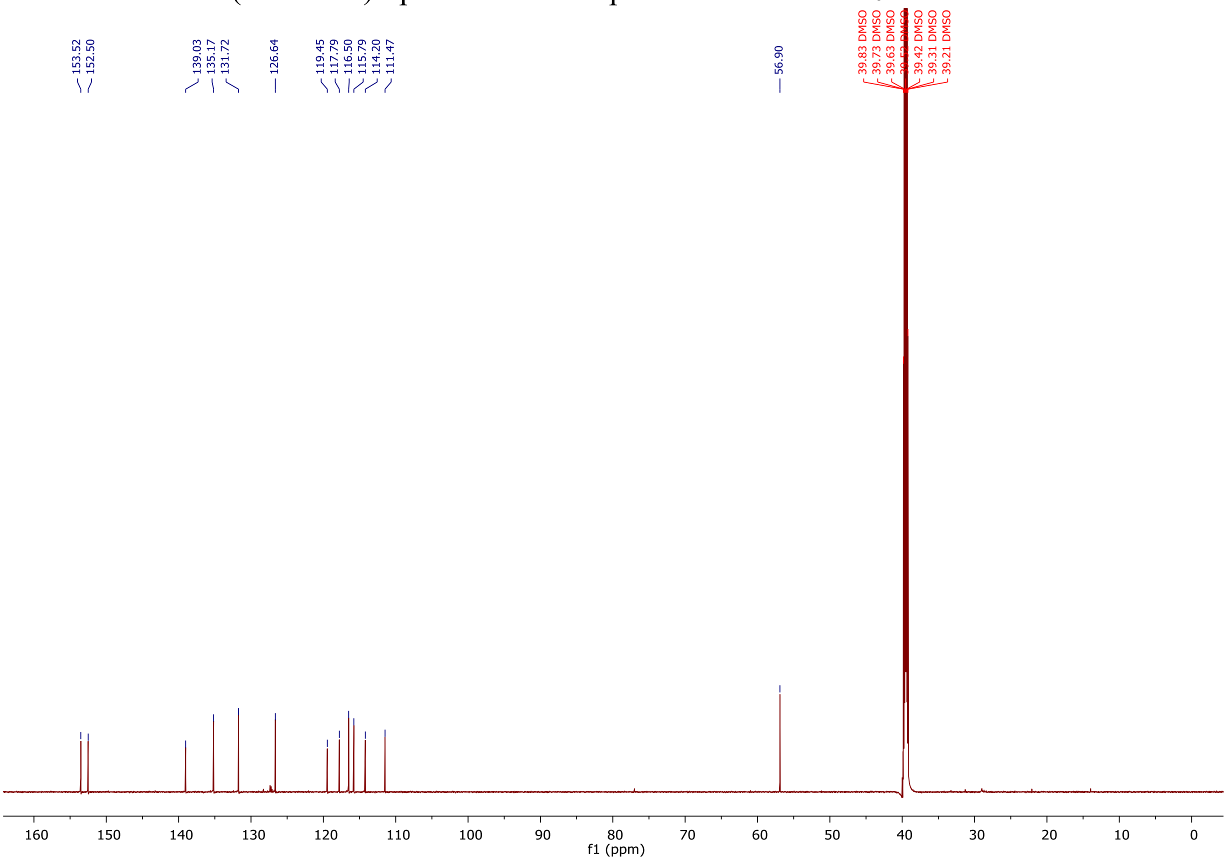

S11: COSY Spectrum of Compound **2** in DMSO-d_6

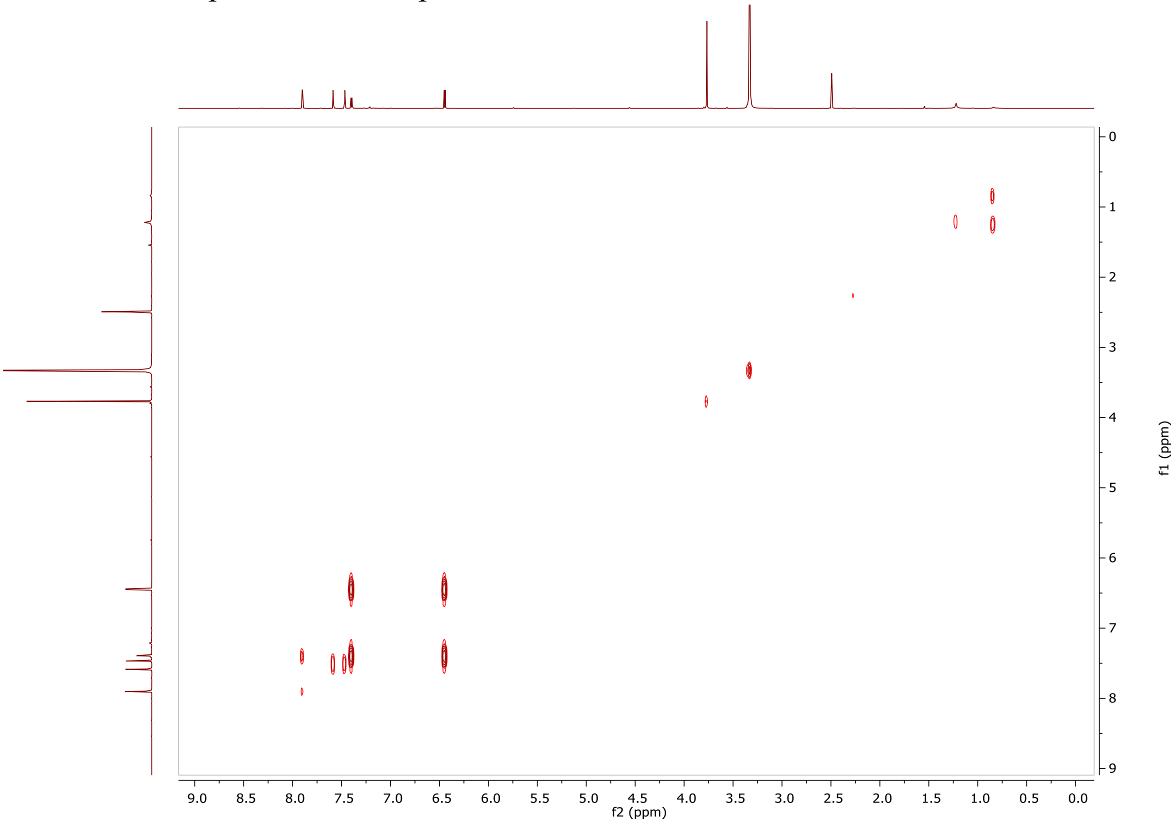

S12: HSQC Spectrum of Compound **2** in DMSO-d_6

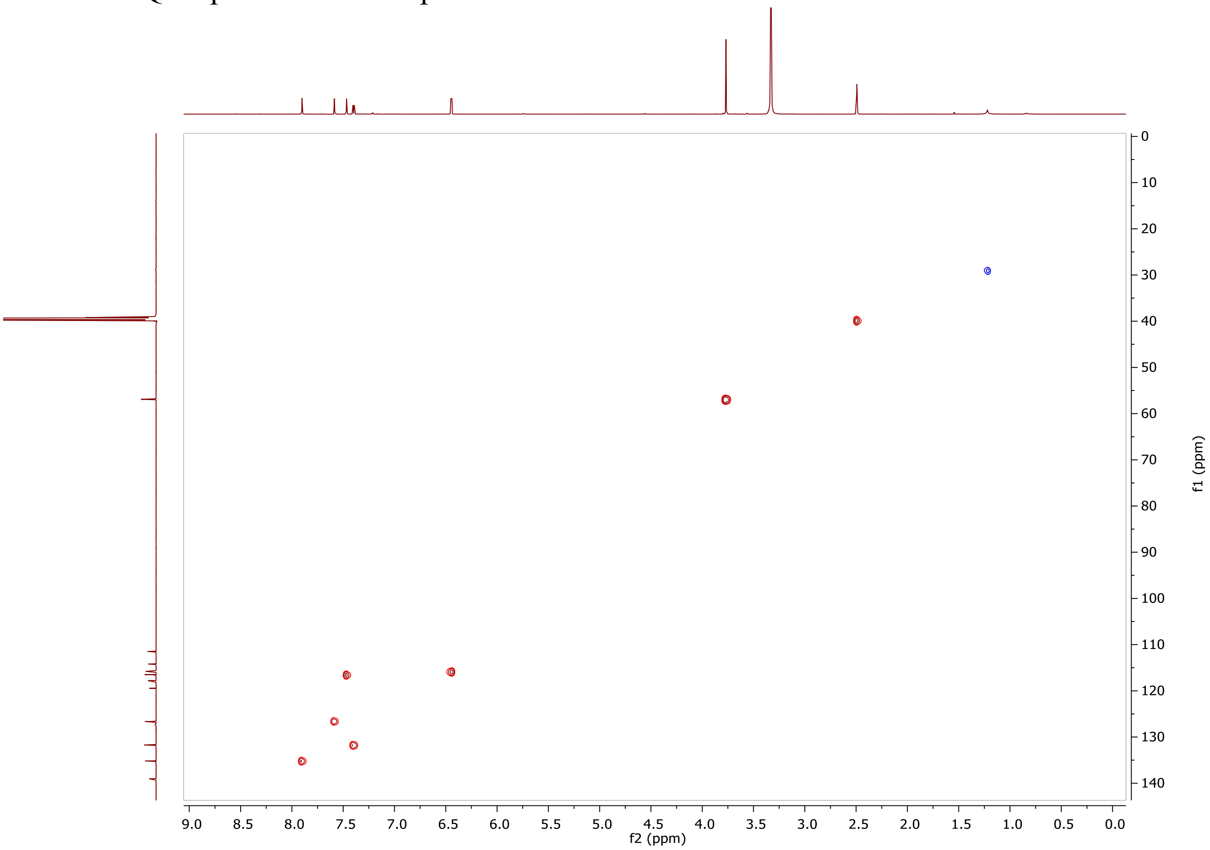

S13: HMBC Spectrum of Compound **2** in DMSO-*d*6

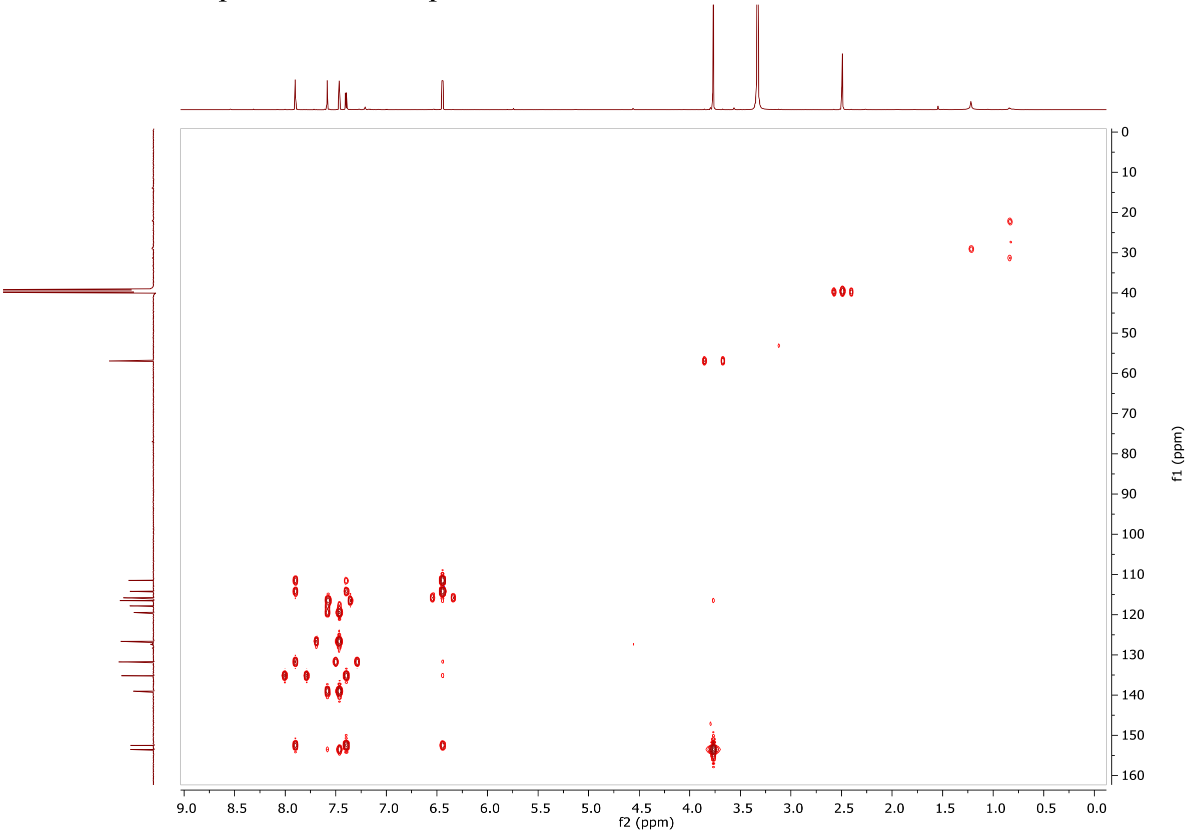

S14: ROESY Spectrum of Compound **2** in DMSO-*d*6

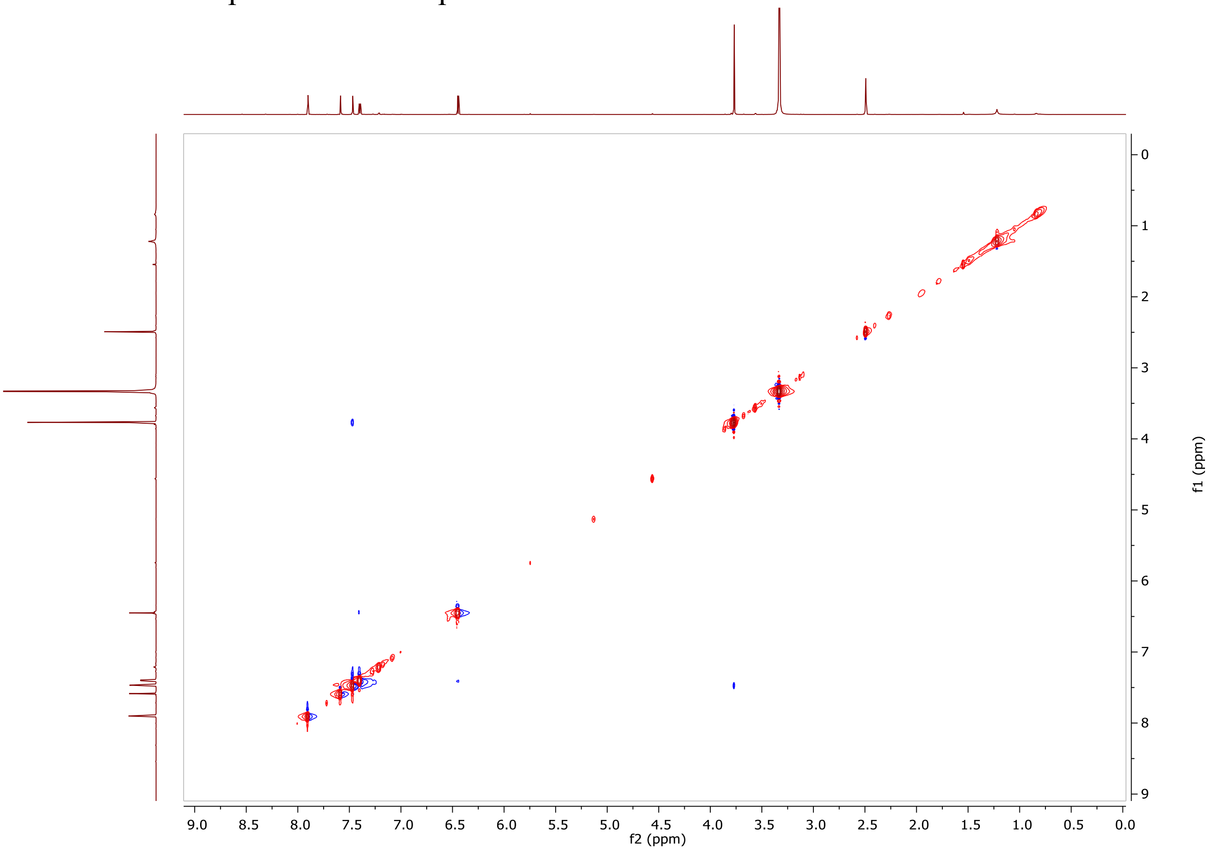

S15: ^{1}H NMR (800 MHz) Spectrum of Compound **3** in DMSO-*d*$_6$

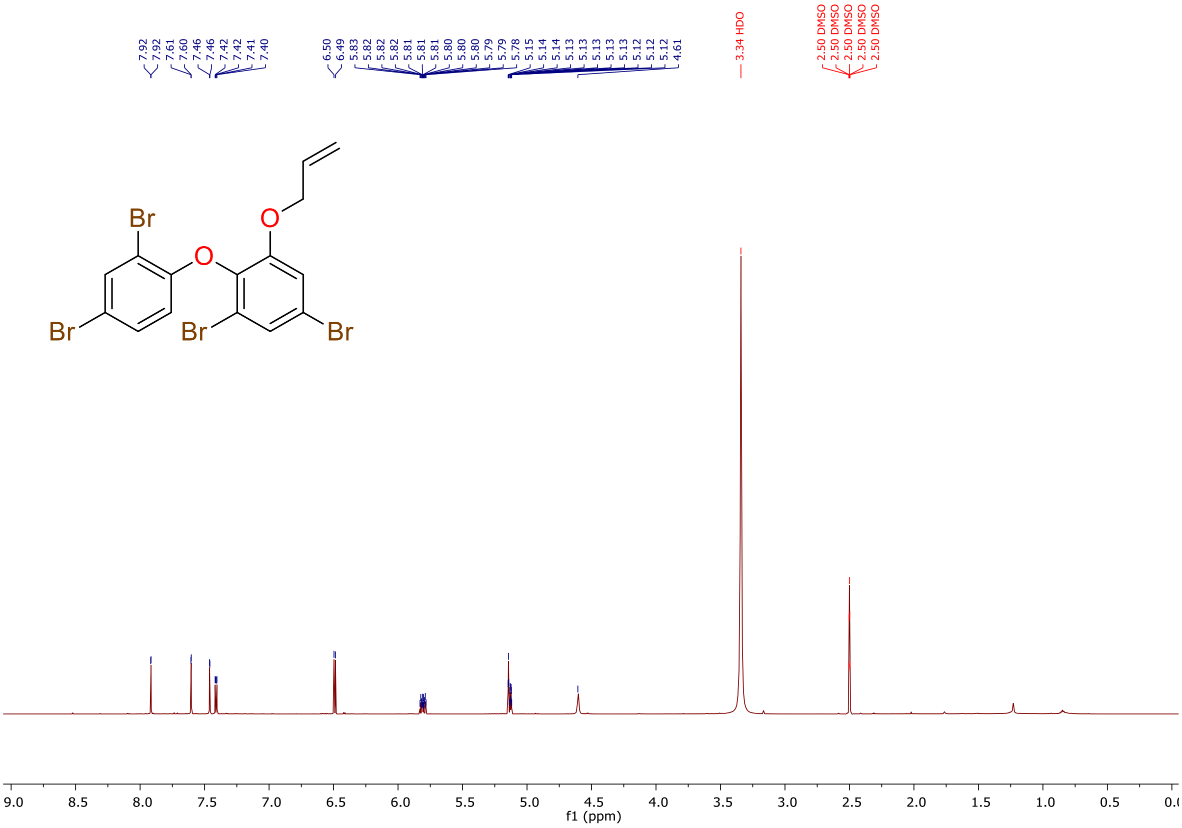

S16: ^{13}C NMR (200 MHz) Spectrum of Compound **3** in DMSO-*d*$_6$

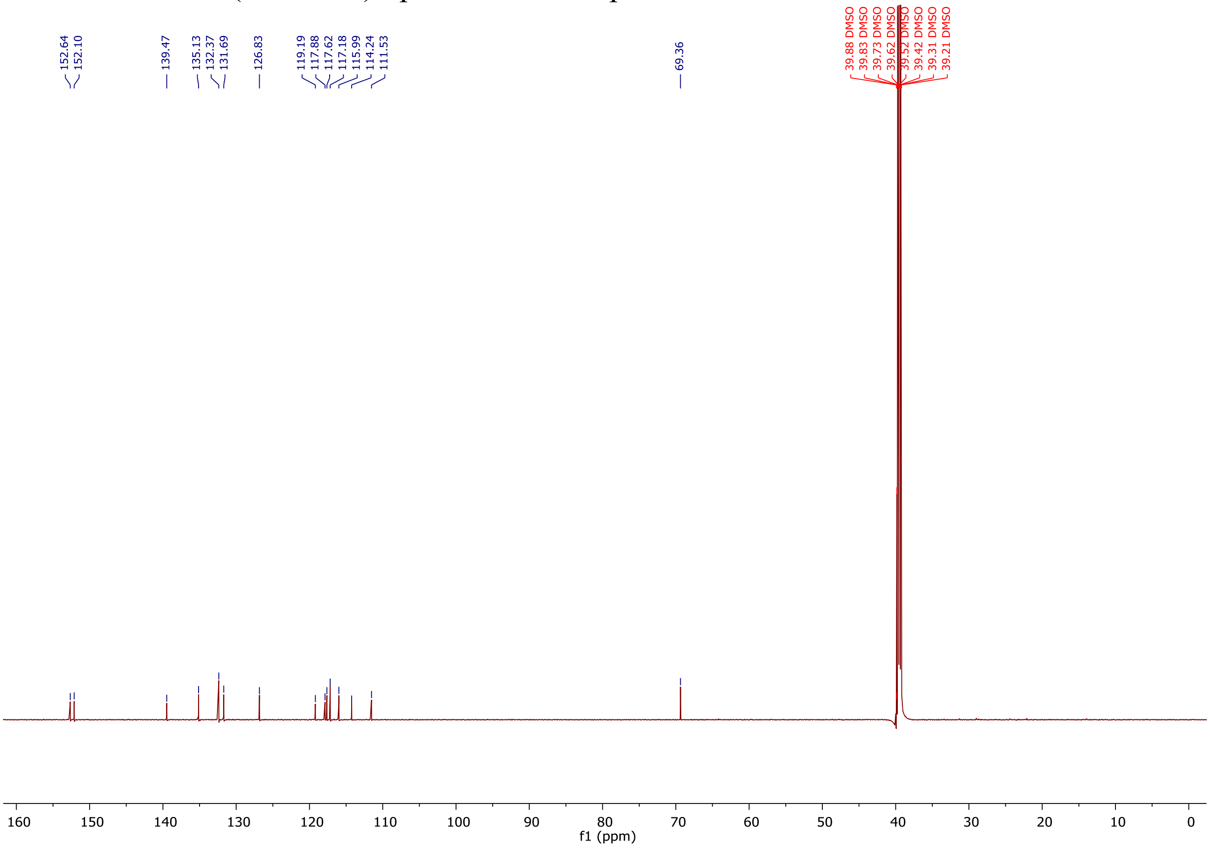

S17: COSY Spectrum of Compound **3** in DMSO-d_6

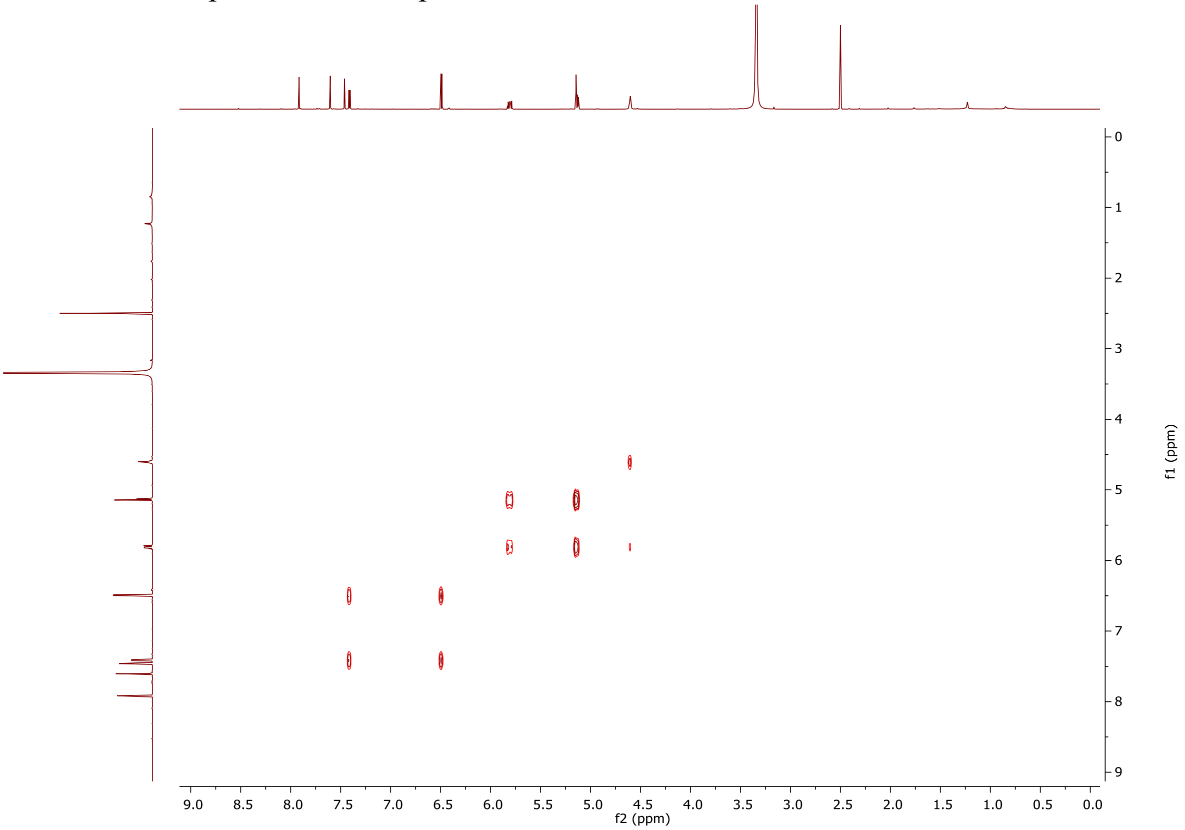

S18: HSQC Spectrum of Compound **3** in DMSO-d_6

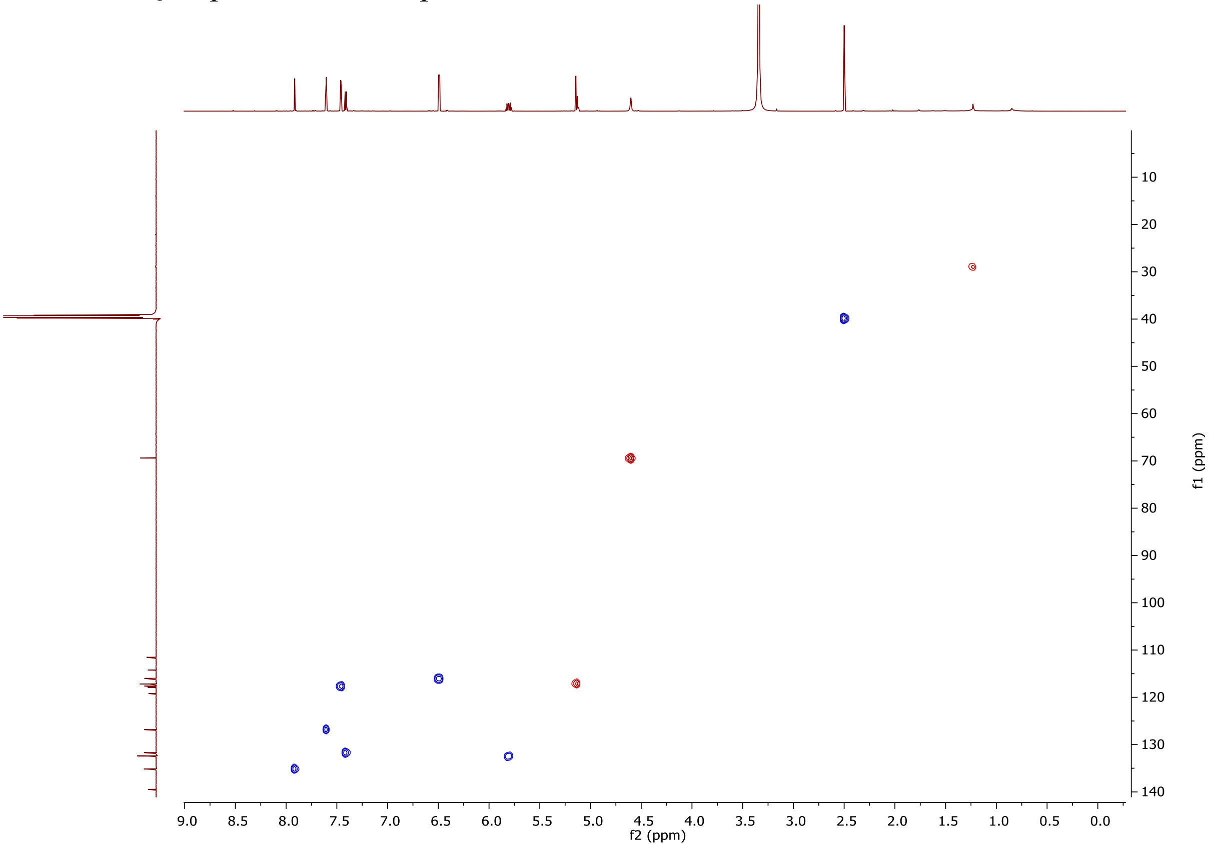

S19: HMBC Spectrum of Compound **3** in DMSO-*d*6

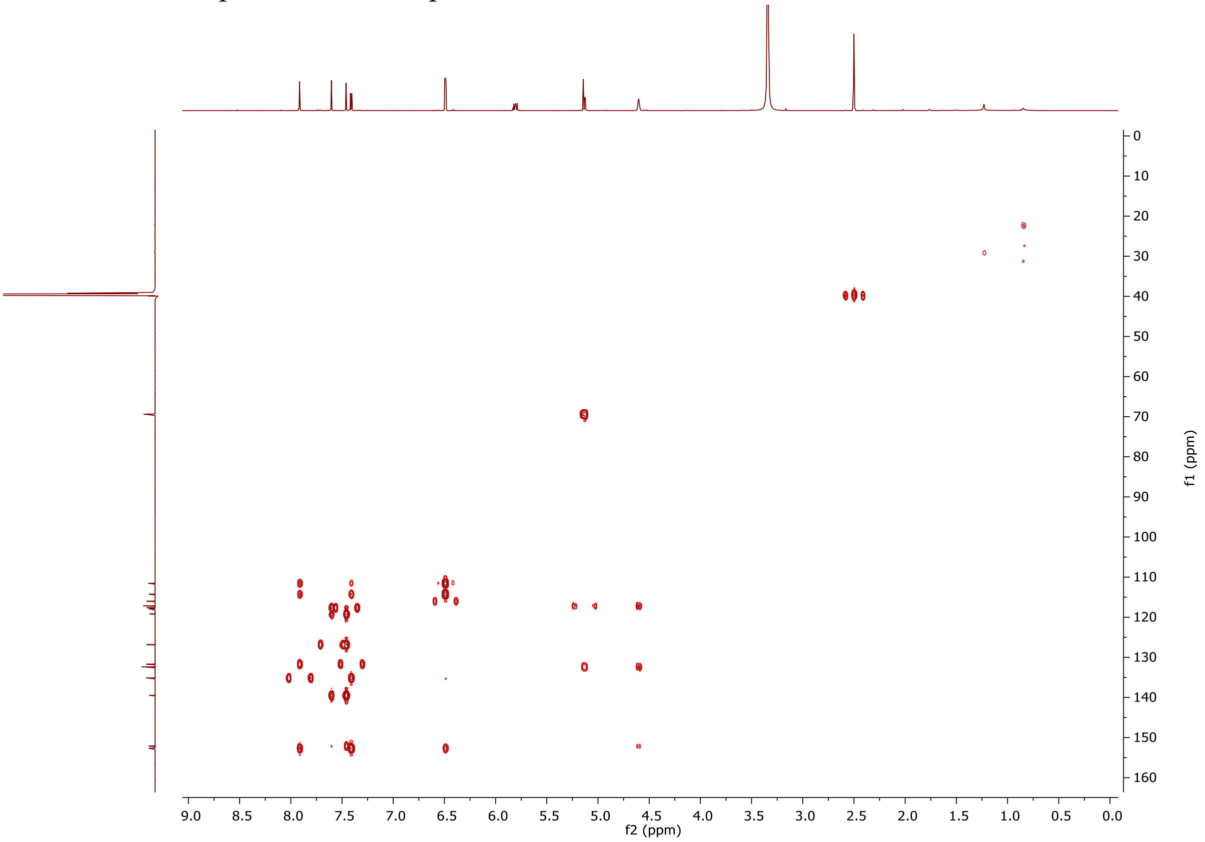

S20: ROESY Spectrum of Compound **3** in DMSO-*d*6

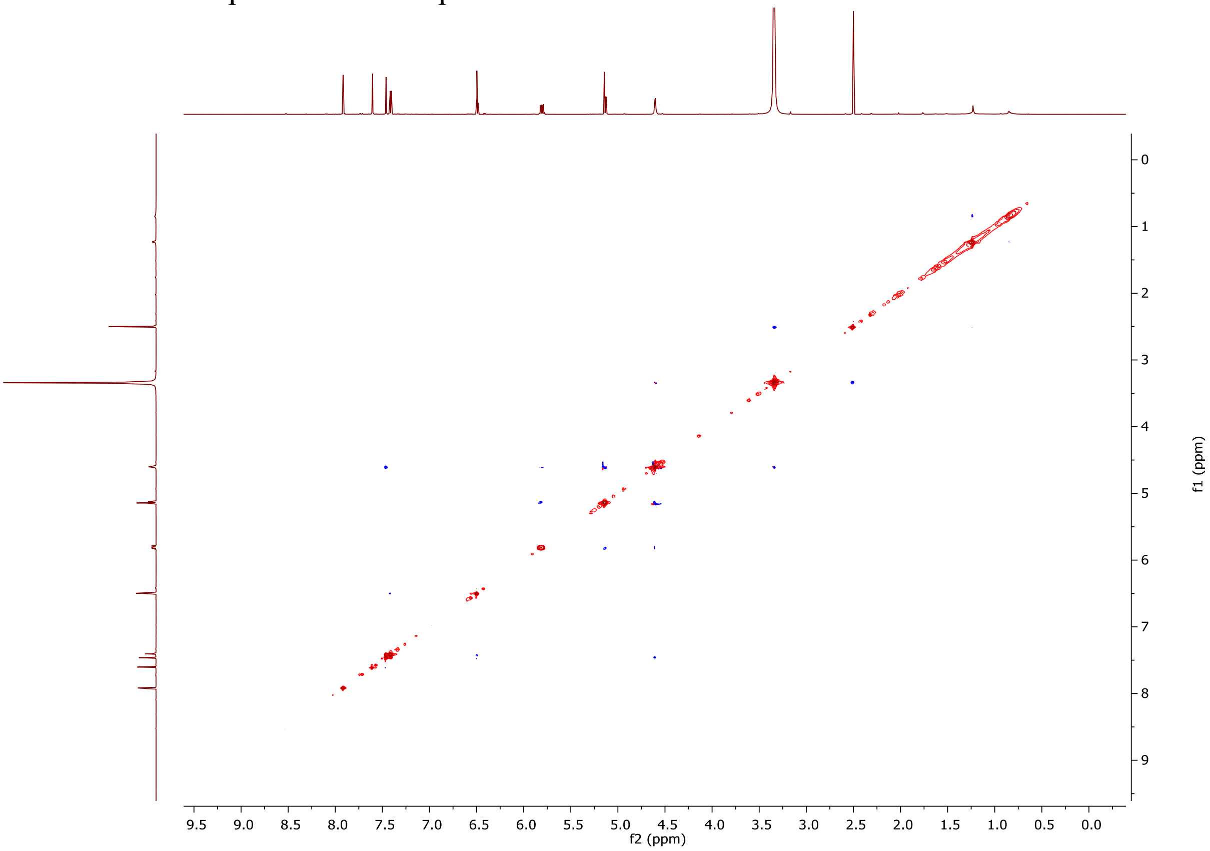

S21: ^{1}H NMR (800 MHz) Spectrum of Compound **4** in DMSO-d_6

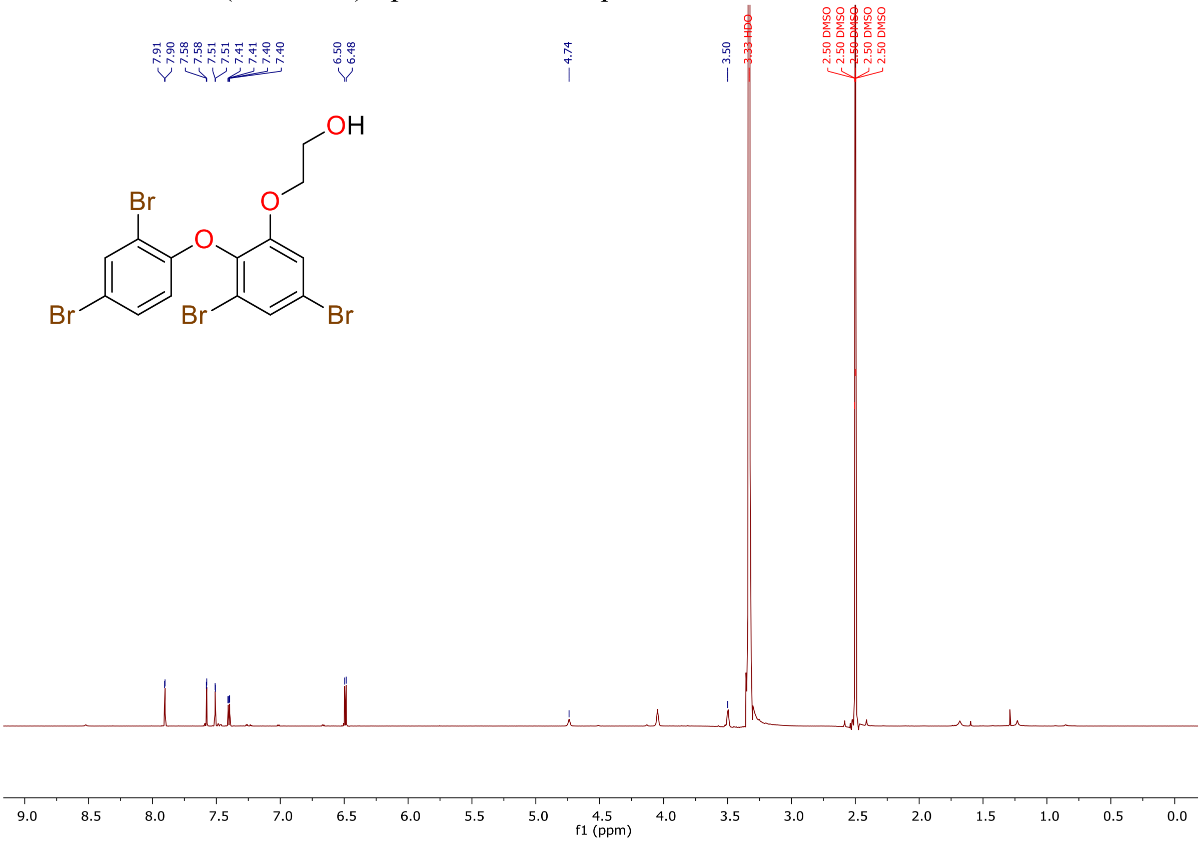

S22: ^{13}C NMR (200 MHz) Spectrum of Compound **4** in DMSO-d_6

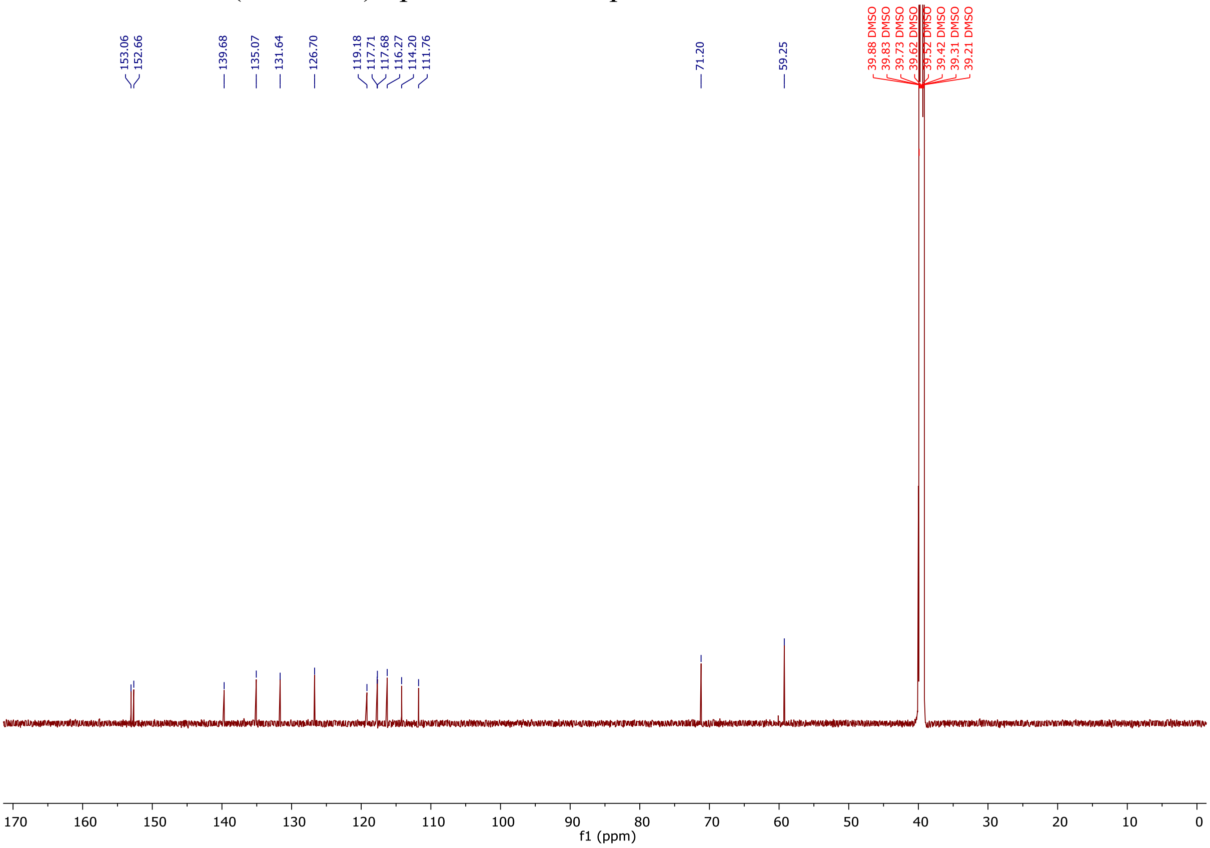

S23: COSY Spectrum of Compound **4** in DMSO-d_6

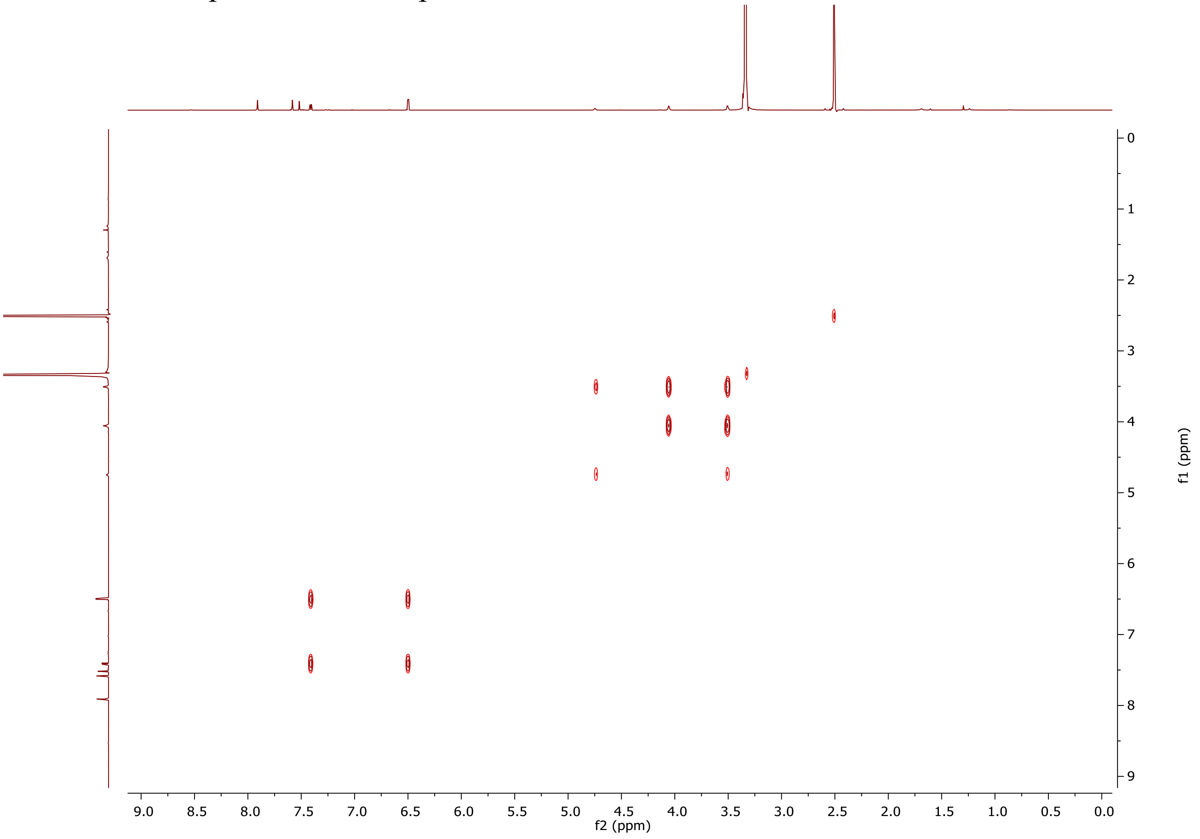

S24: HSQC Spectrum of Compound **4** in DMSO-d_6

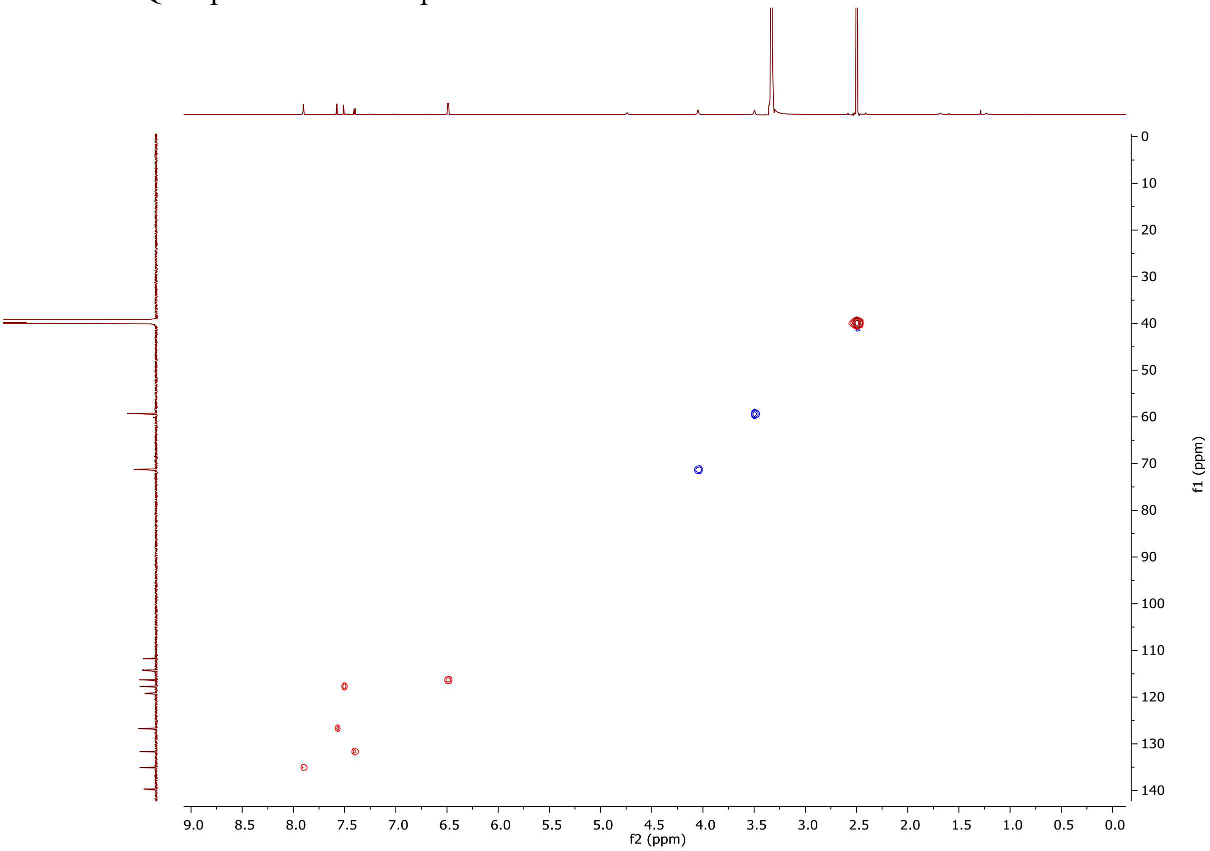

S25: HMBC Spectrum of Compound **4** in DMSO-d_6

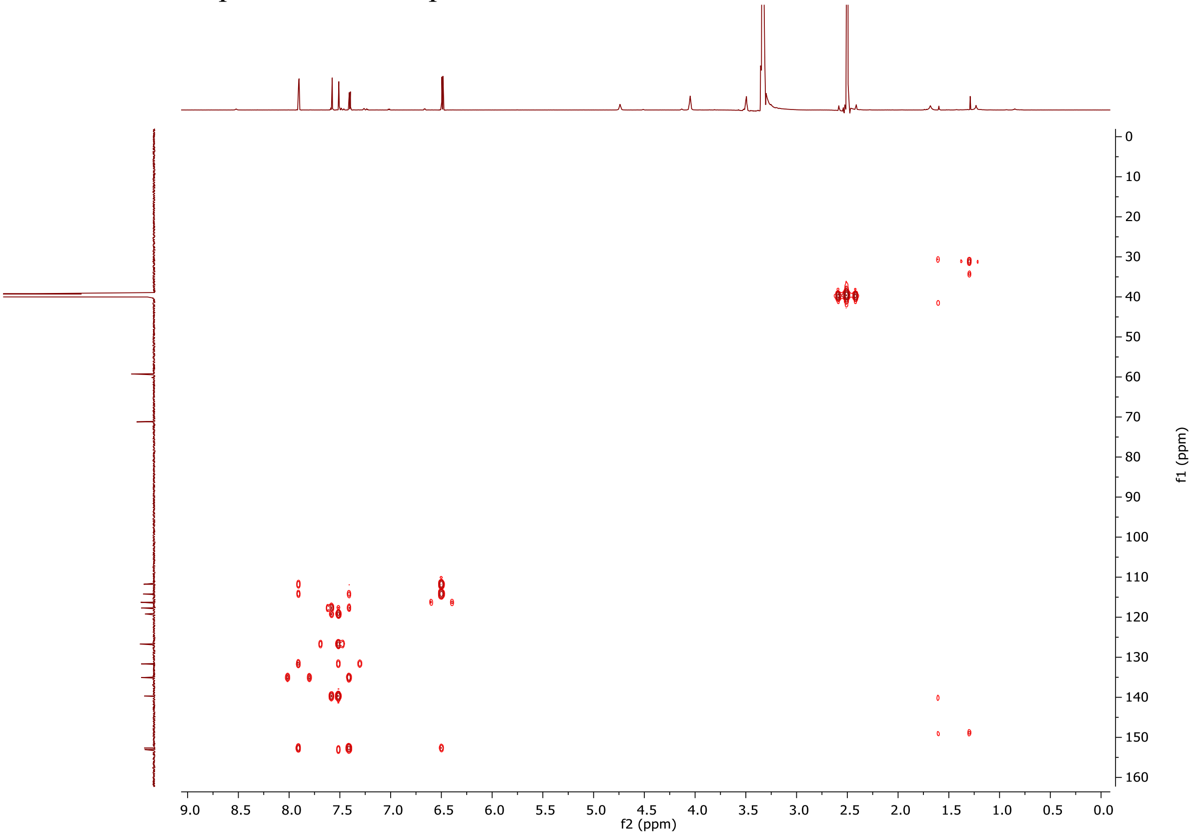

S26: ROESY Spectrum of Compound **4** in DMSO-d_6

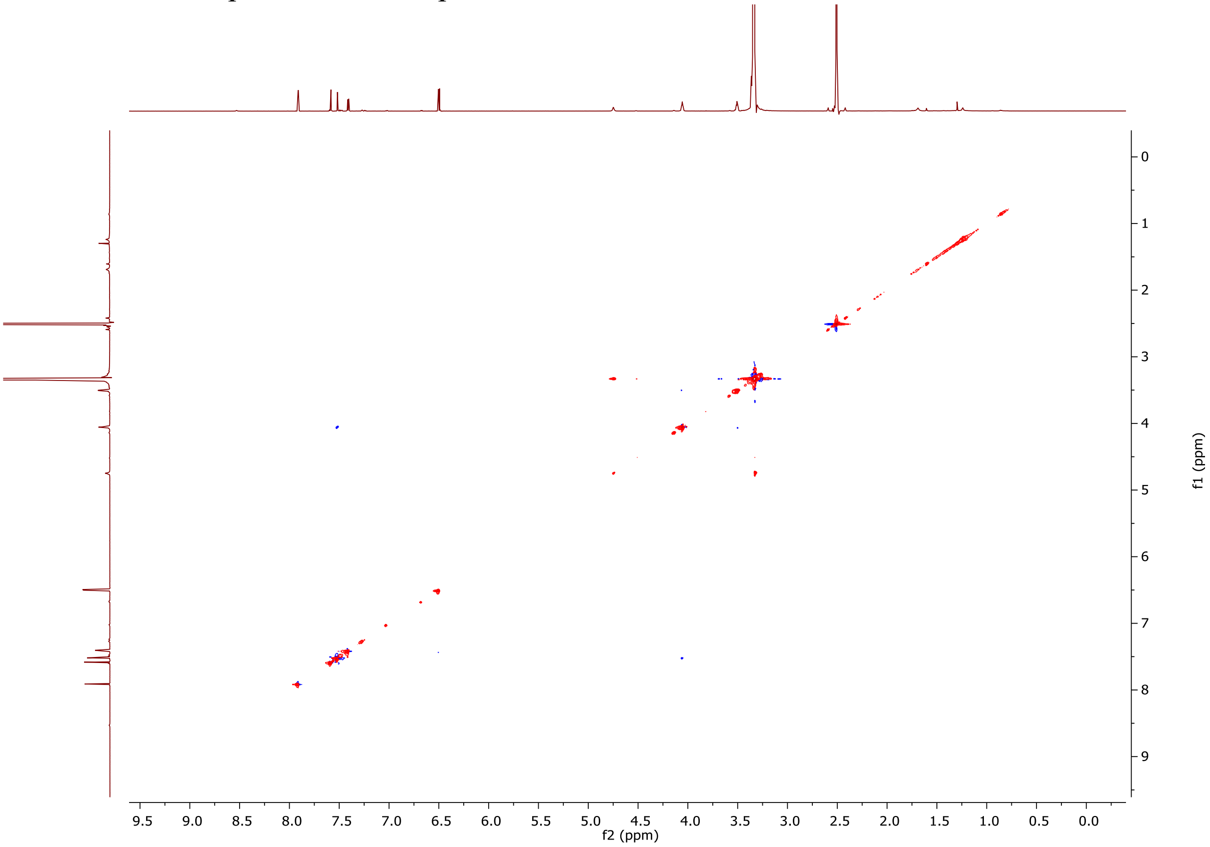

S27: ^{1}H NMR (800 MHz) Spectrum of Compound **5** in DMSO-d_6

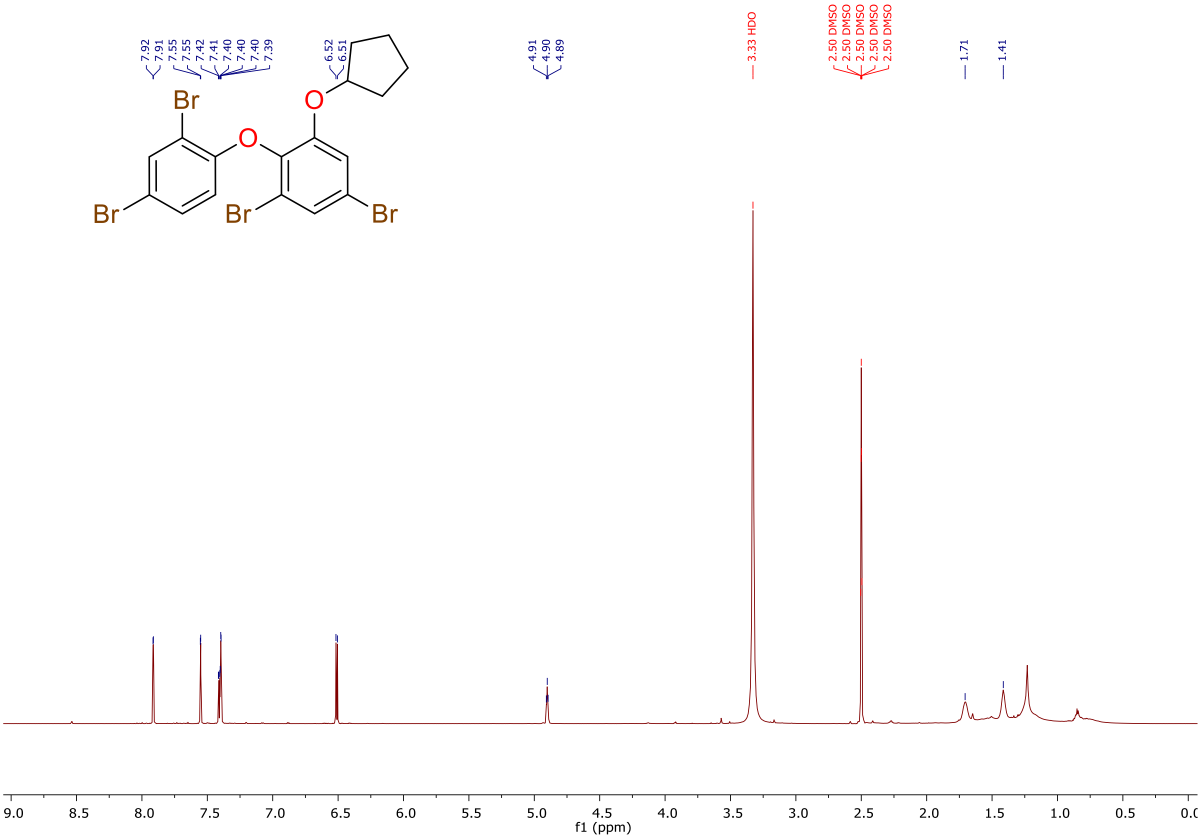

S28: ^{13}C NMR (200 MHz) Spectrum of Compound **5** in DMSO-d_6

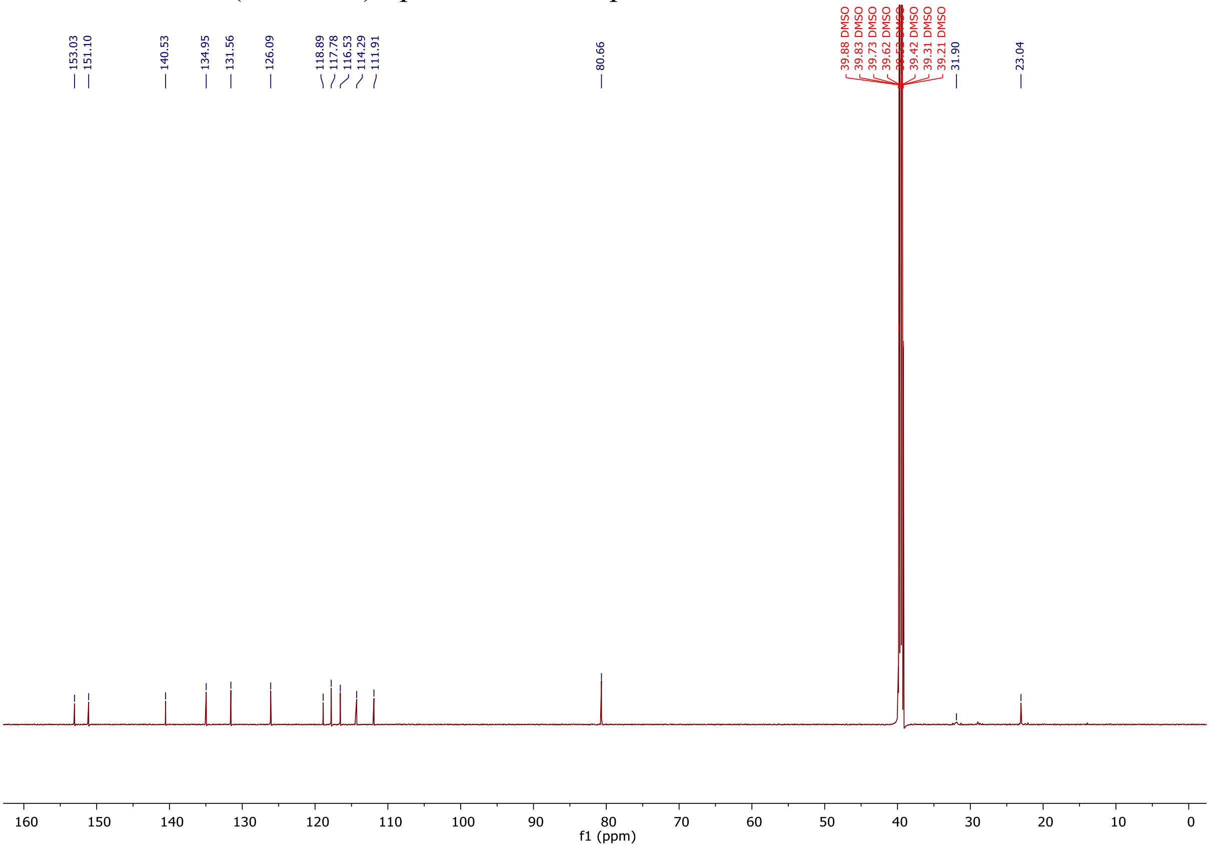

S29: COSY Spectrum of Compound **5** in DMSO-d_6

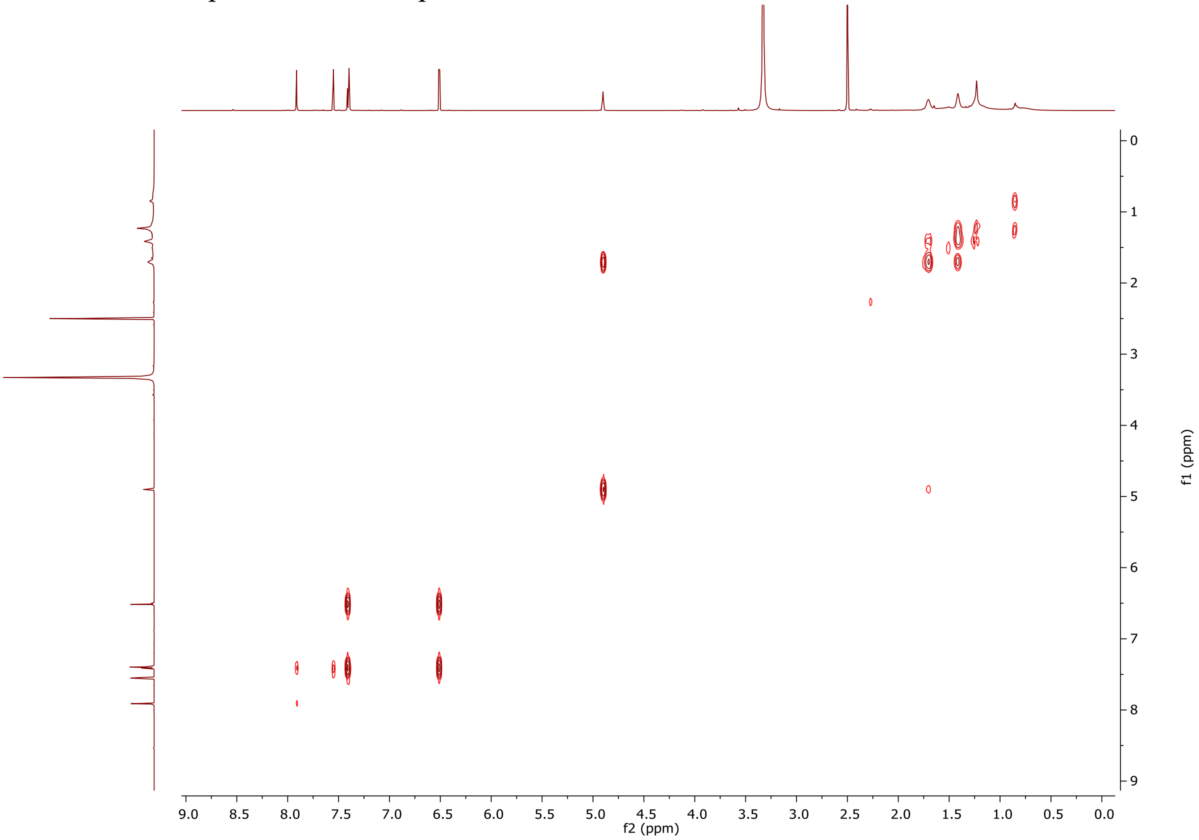

S30: HSQC Spectrum of Compound **5** in DMSO-d_6

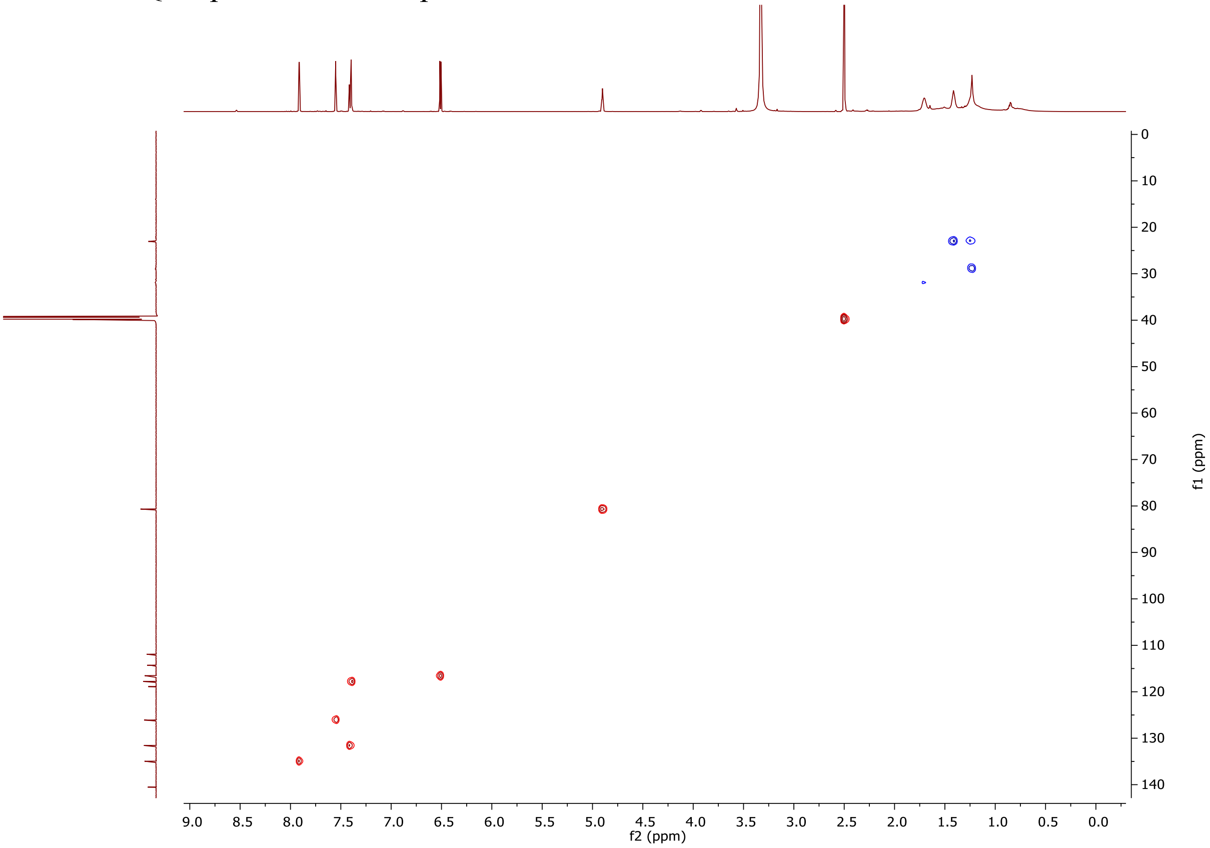

S31: HMBC Spectrum of Compound **5** in DMSO-*d*6

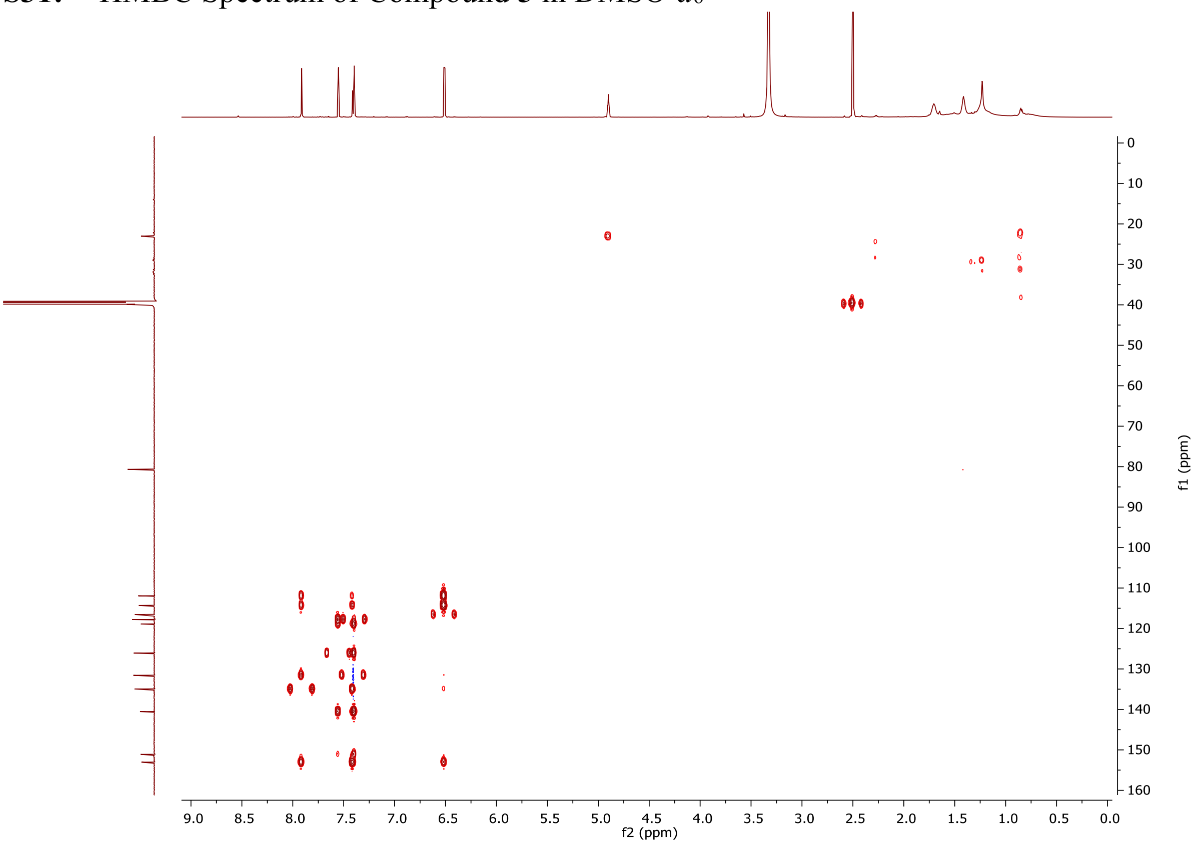

S32: ROESY Spectrum of Compound **5** in DMSO-*d*6

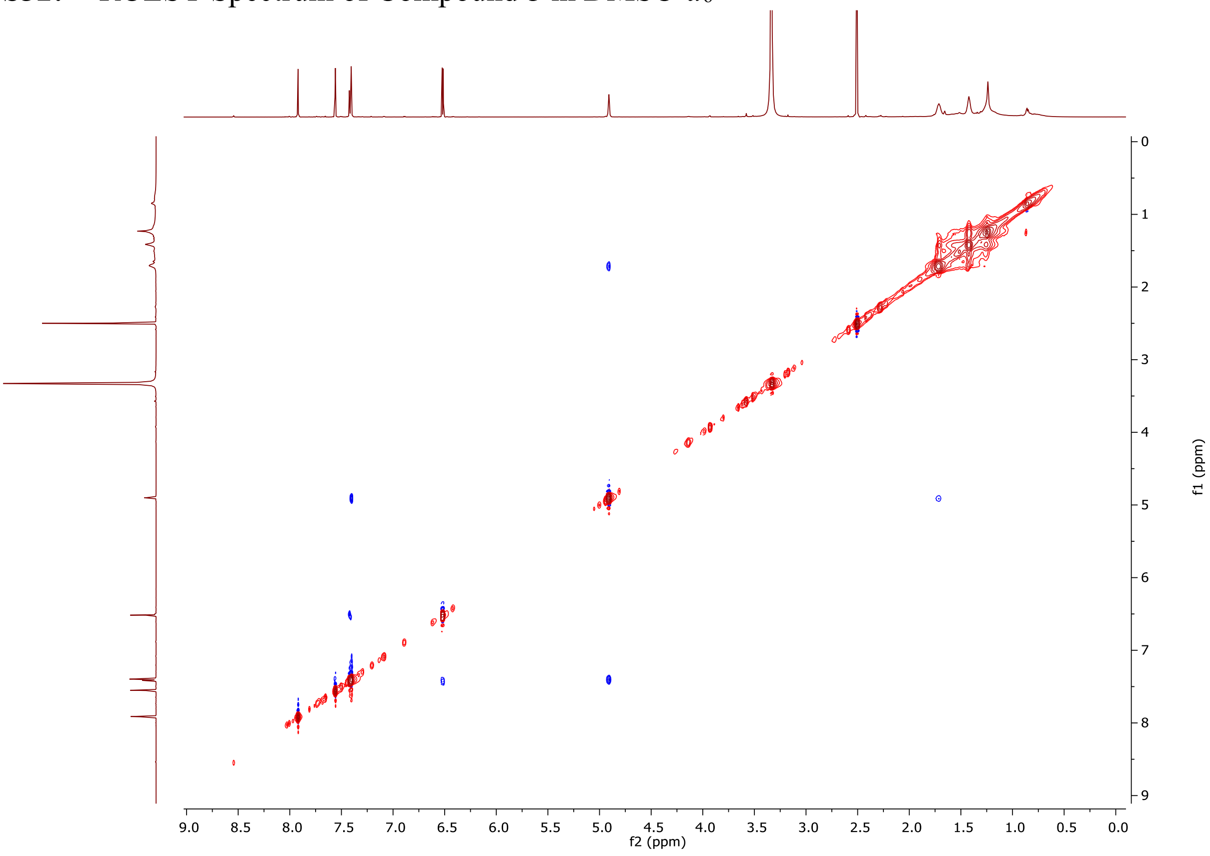

S33: ^{1}H NMR (800 MHz) Spectrum of Compound **6** in DMSO-d_6

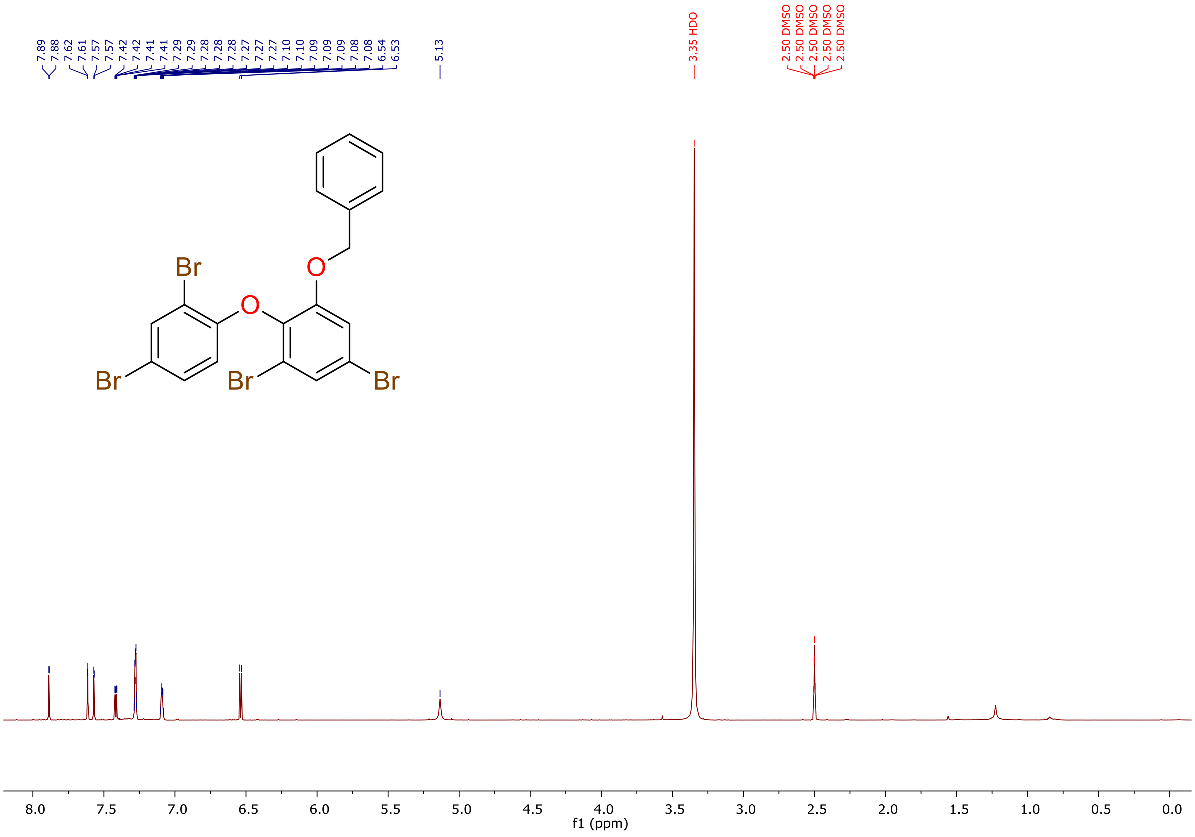

S34: ^{13}C NMR (200 MHz) Spectrum of Compound **6** in DMSO-d_6

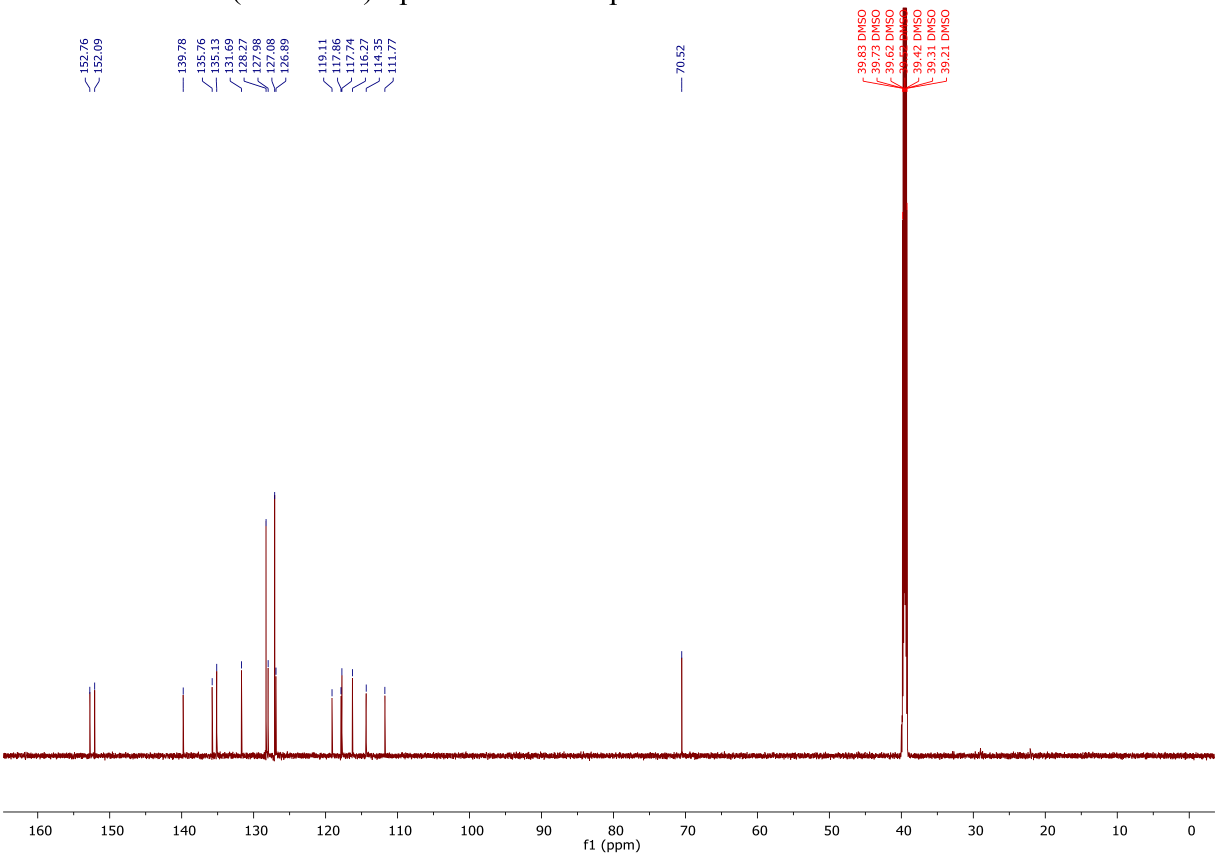

S35: COSY Spectrum of Compound **6** in DMSO-d_6

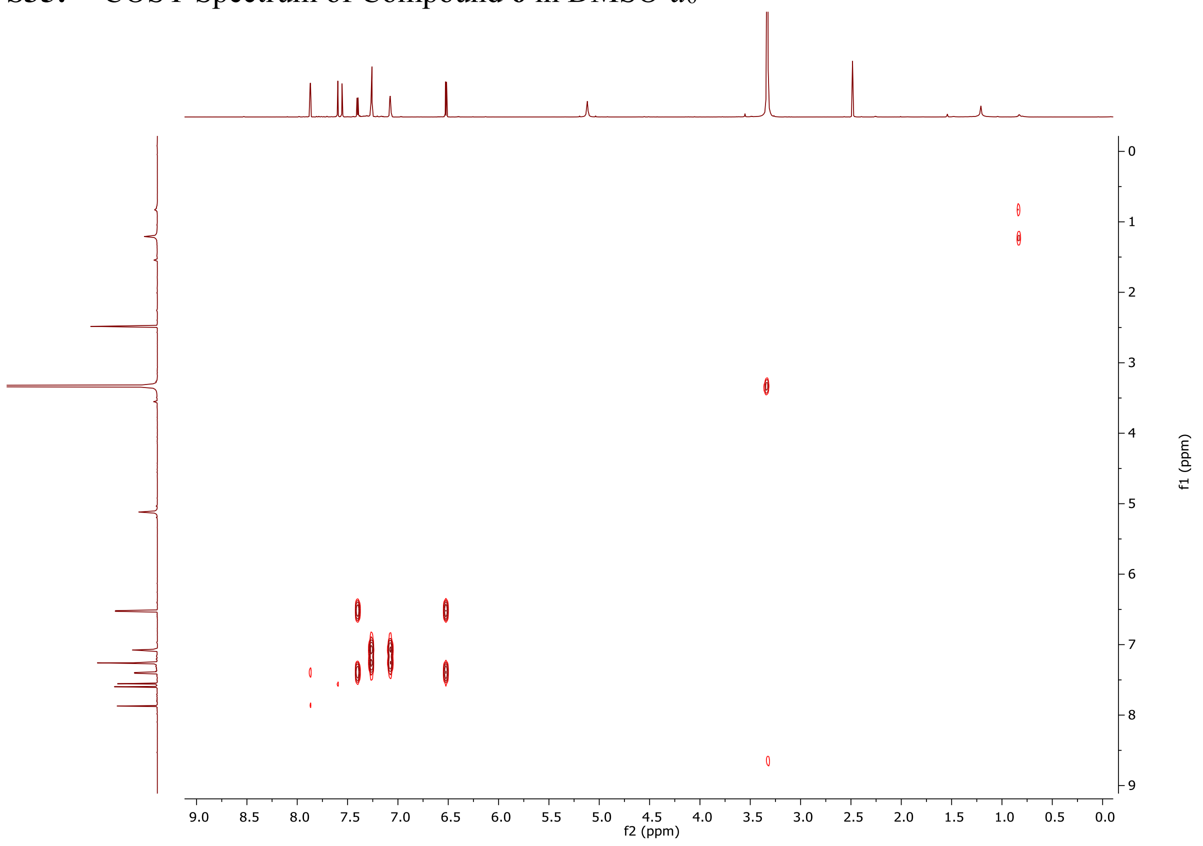

S36: HSQC Spectrum of Compound **6** in DMSO-d_6

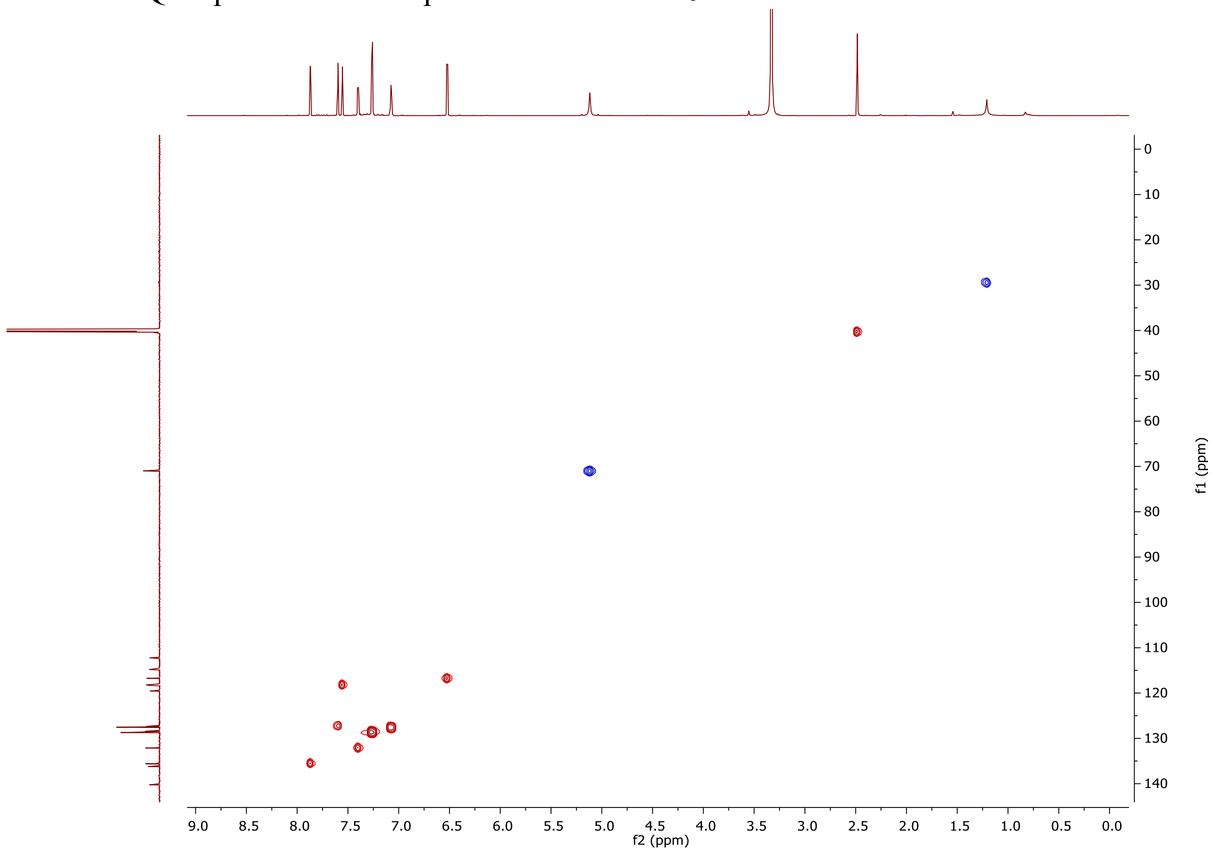

S37: HMBC Spectrum of Compound **6** in DMSO-*d*6

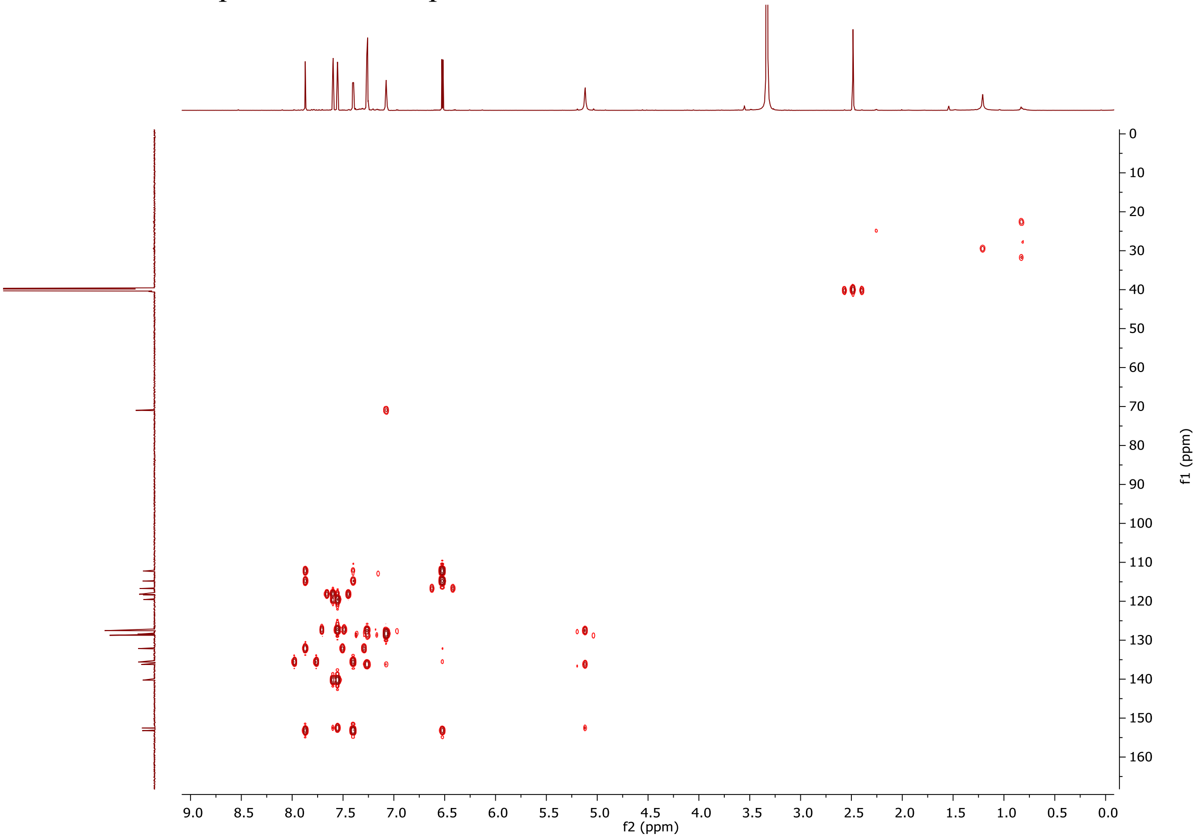

S38: ROESY Spectrum of Compound **6** in DMSO-*d*6

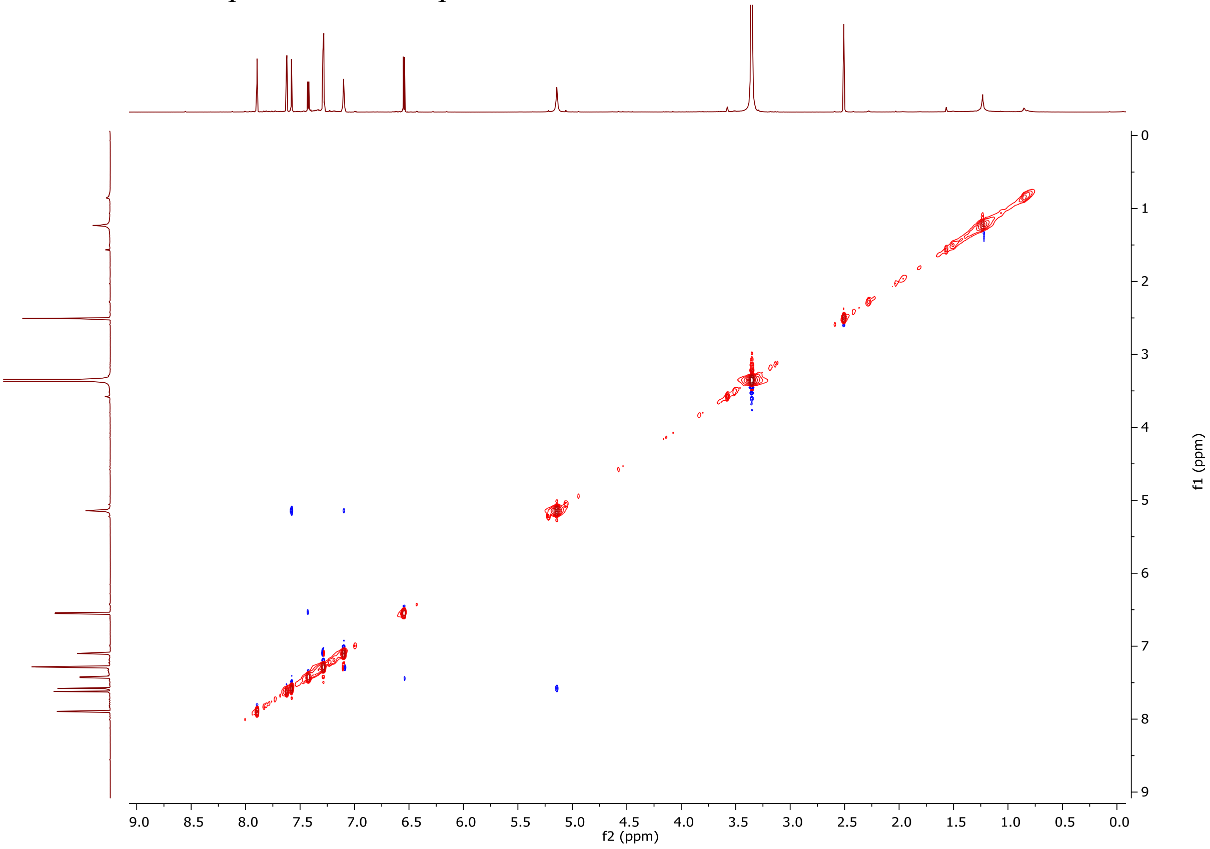

S39: ^{1}H NMR (800 MHz) Spectrum of Compound **7** in DMSO-d_6

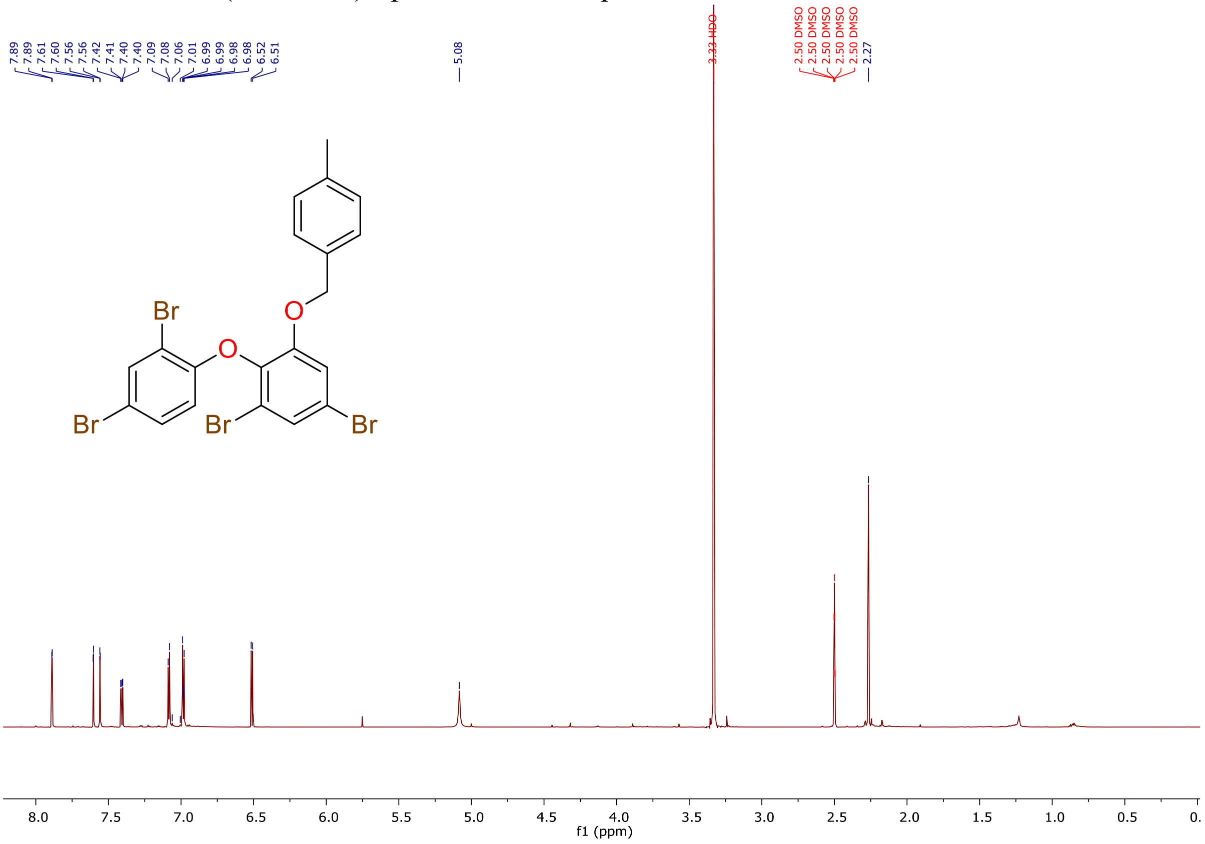

S40: ^{13}C NMR (200 MHz) Spectrum of Compound **7** in DMSO-d_6

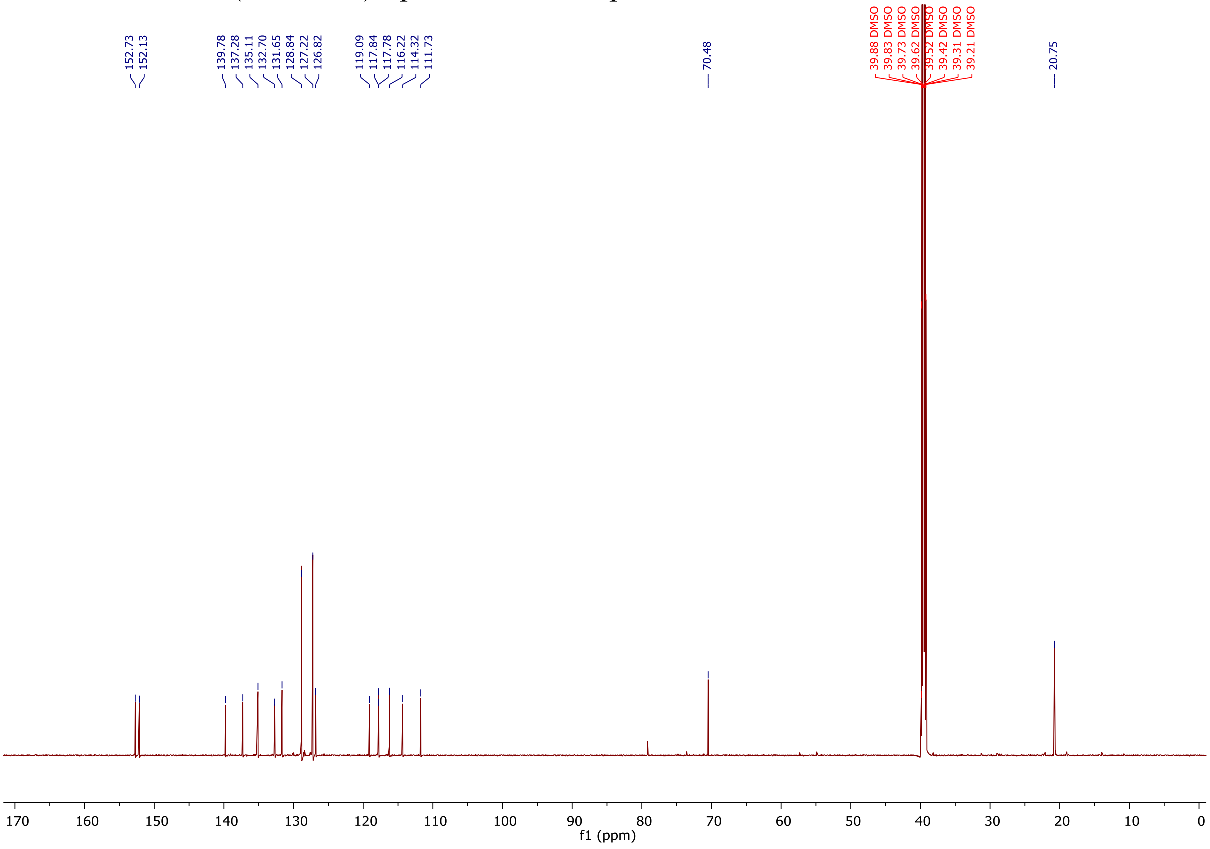

S41: COSY Spectrum of Compound **7** in DMSO-d_6

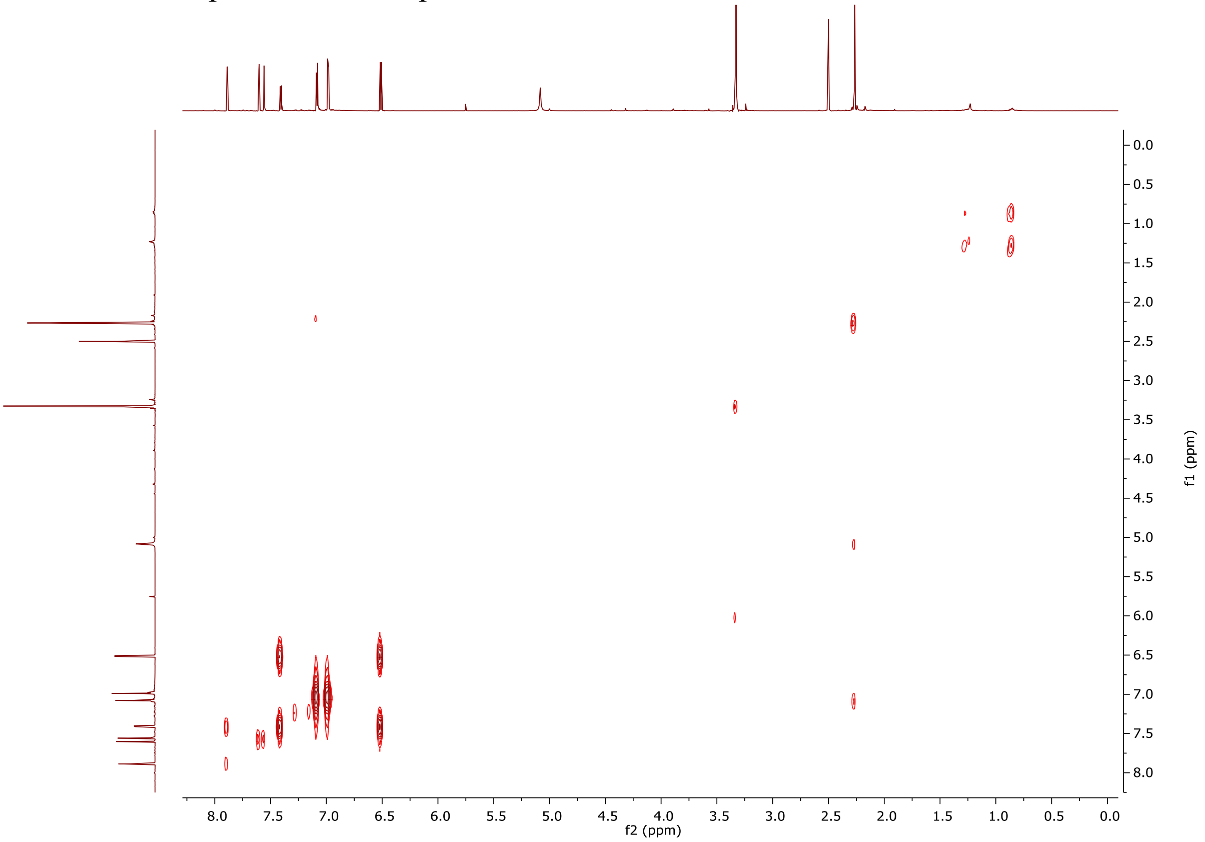

S42: HSQC Spectrum of Compound **7** in DMSO-d_6

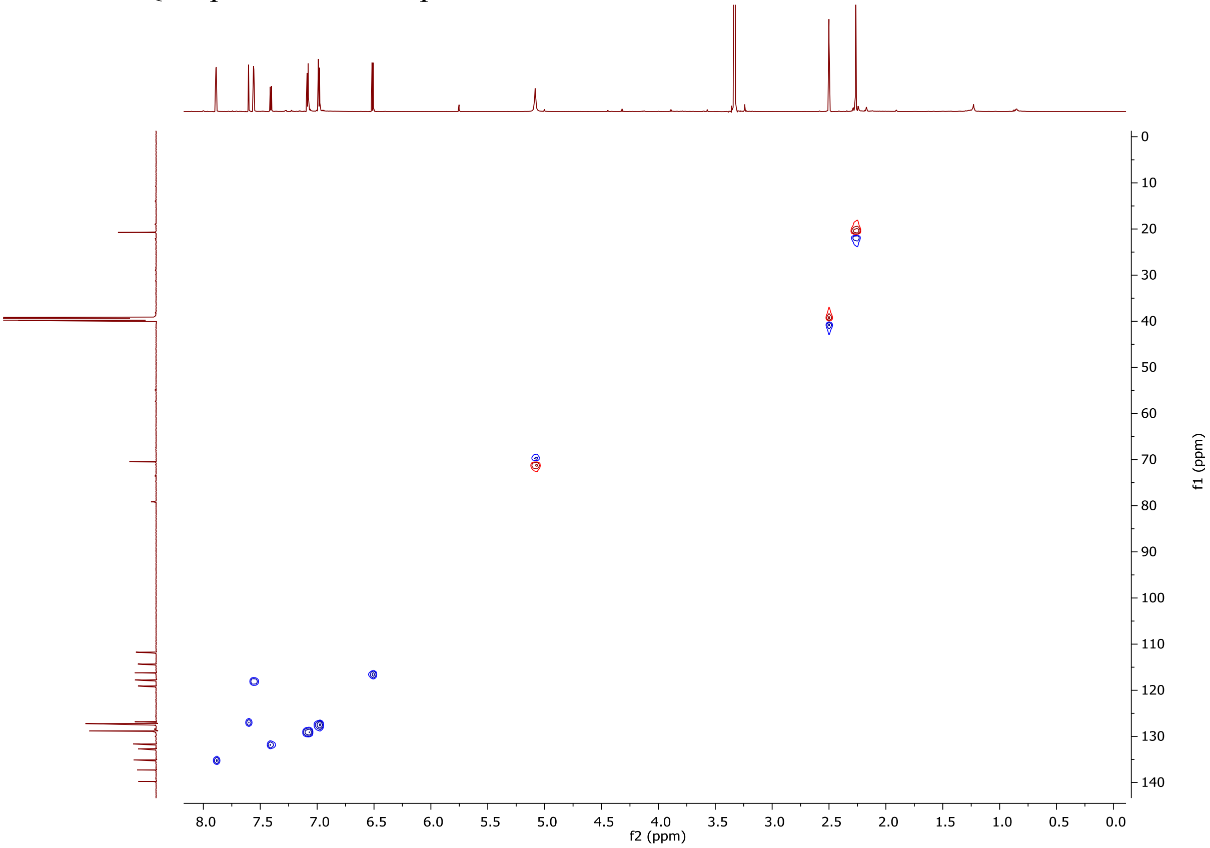

S43: HMBC Spectrum of Compound **7** in DMSO-*d*6

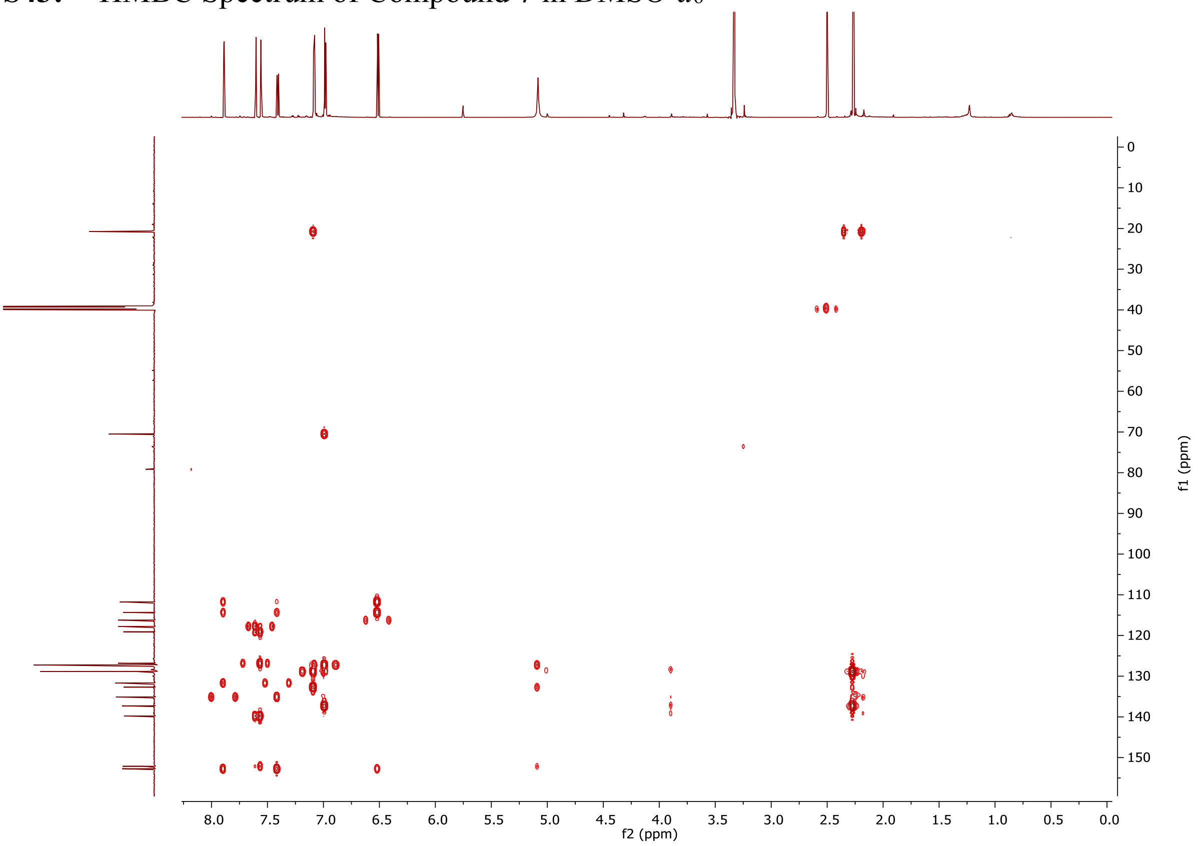

S44: ROESY Spectrum of Compound **7** in DMSO-*d*6

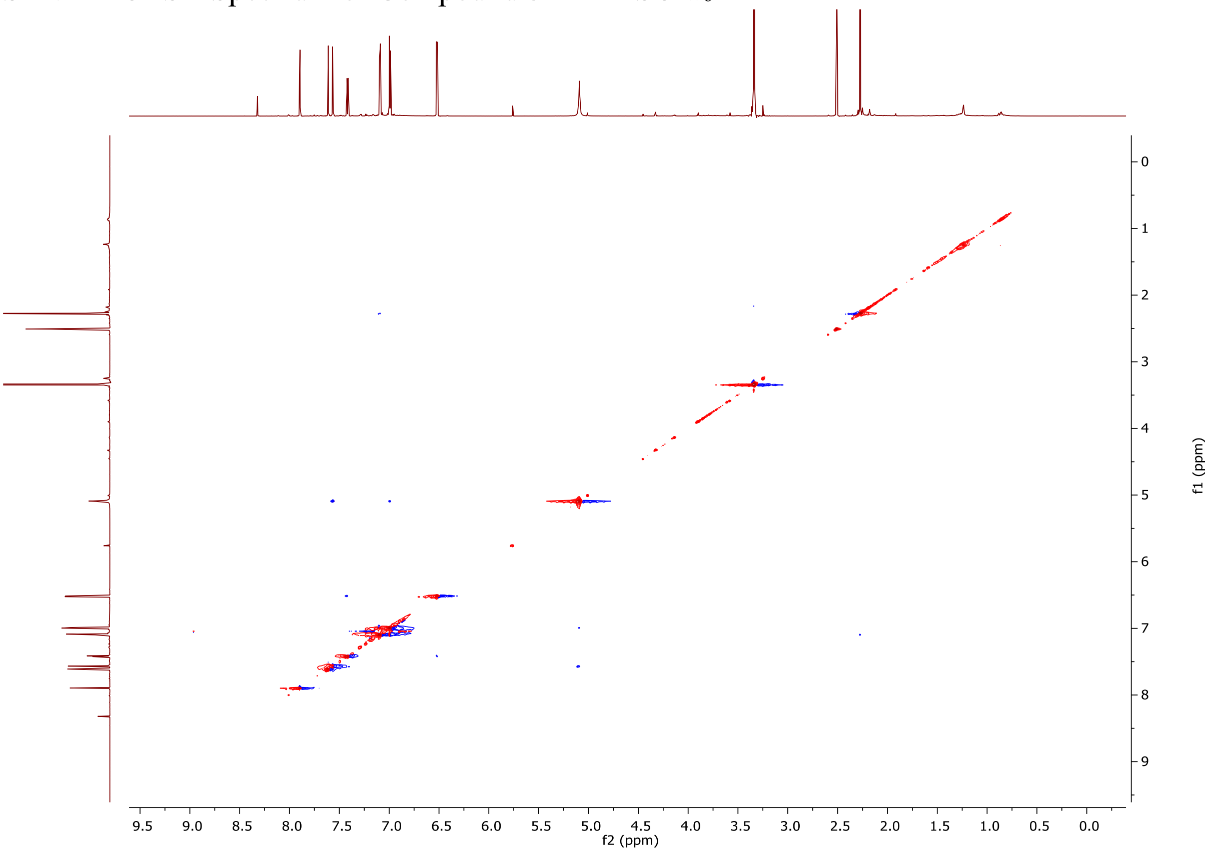

S45: ^{1}H NMR (800 MHz) Spectrum of Compound **8** in DMSO-d_6

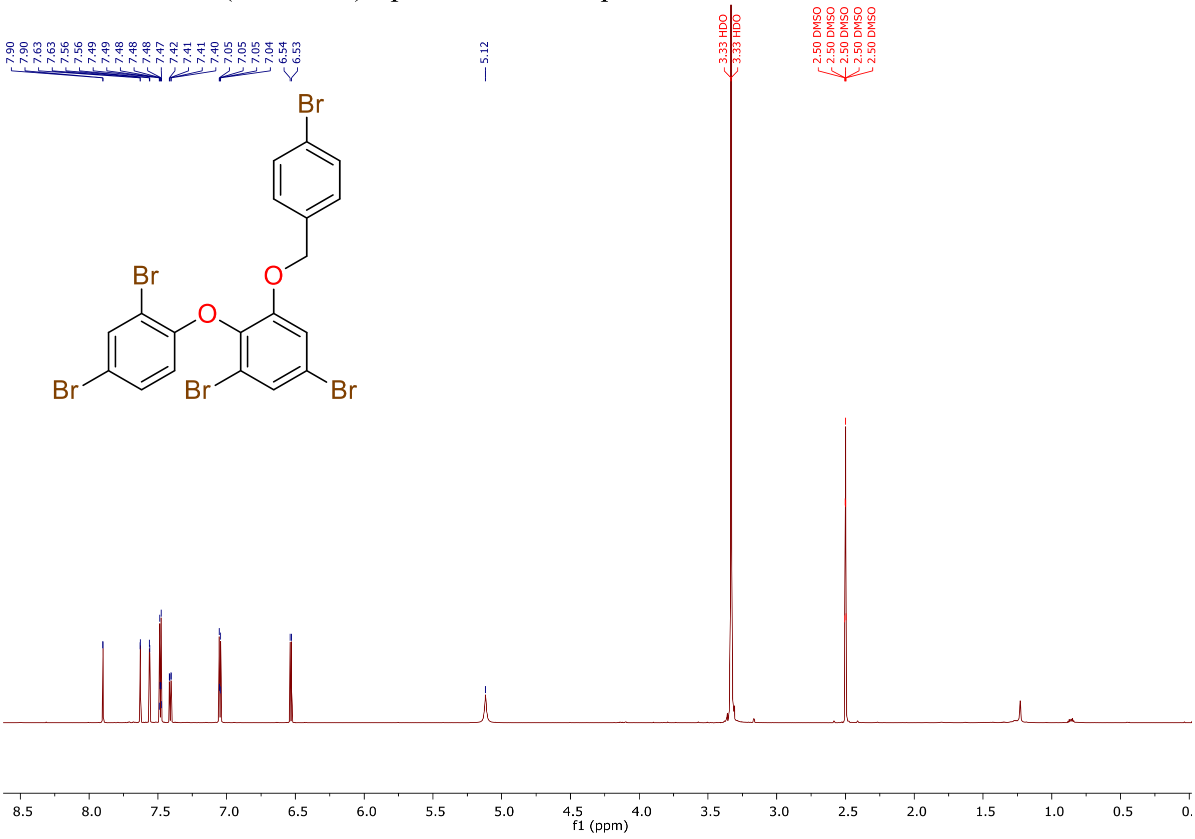

S46: ^{13}C NMR (200 MHz) Spectrum of Compound **8** in DMSO-d_6

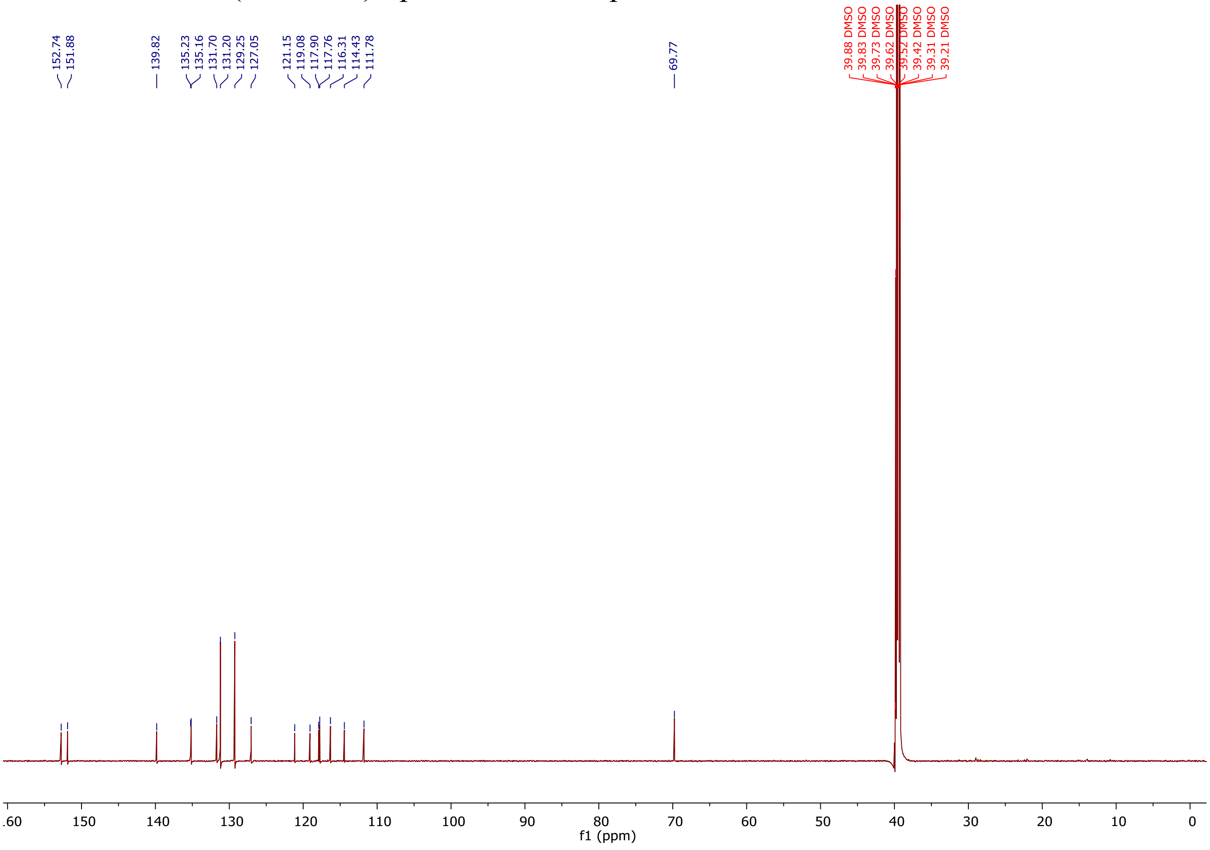

S47: COSY Spectrum of Compound **8** in DMSO-d_6

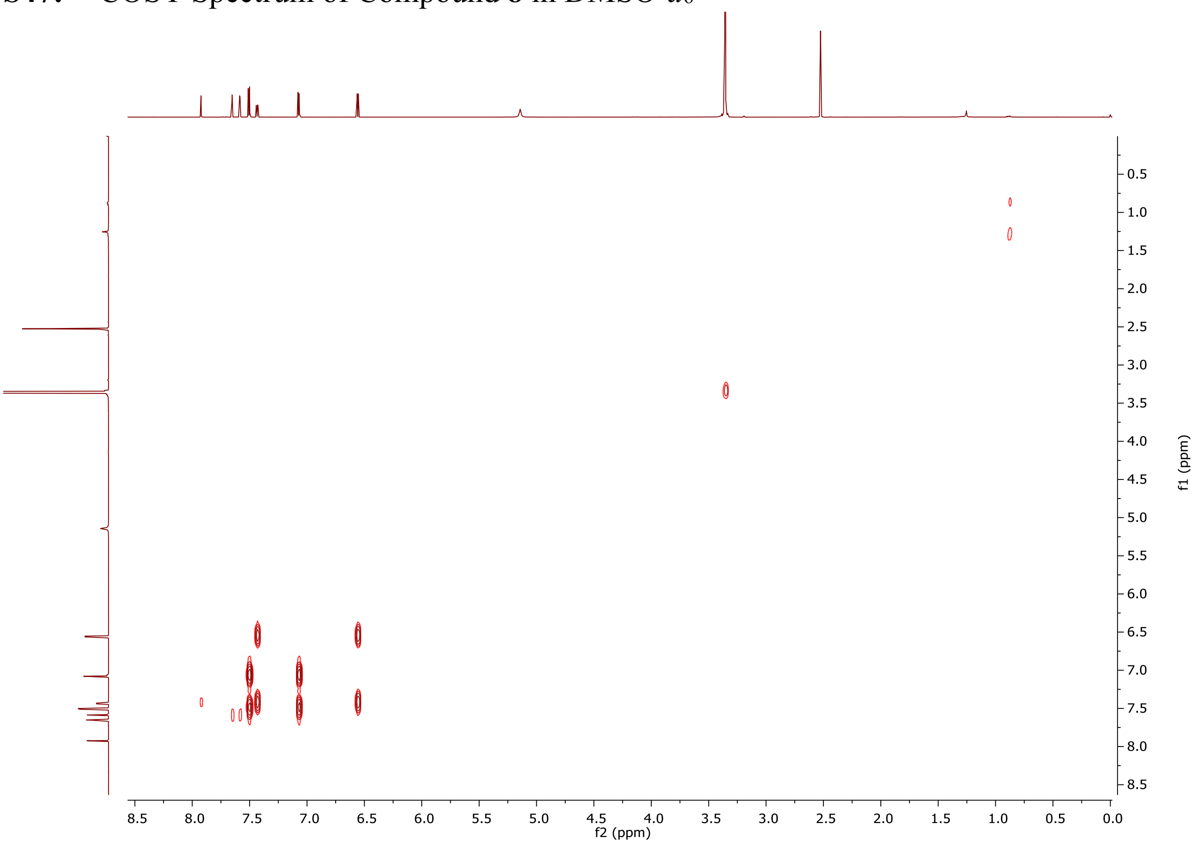

S48: HSQC Spectrum of Compound **8** in DMSO-d_6

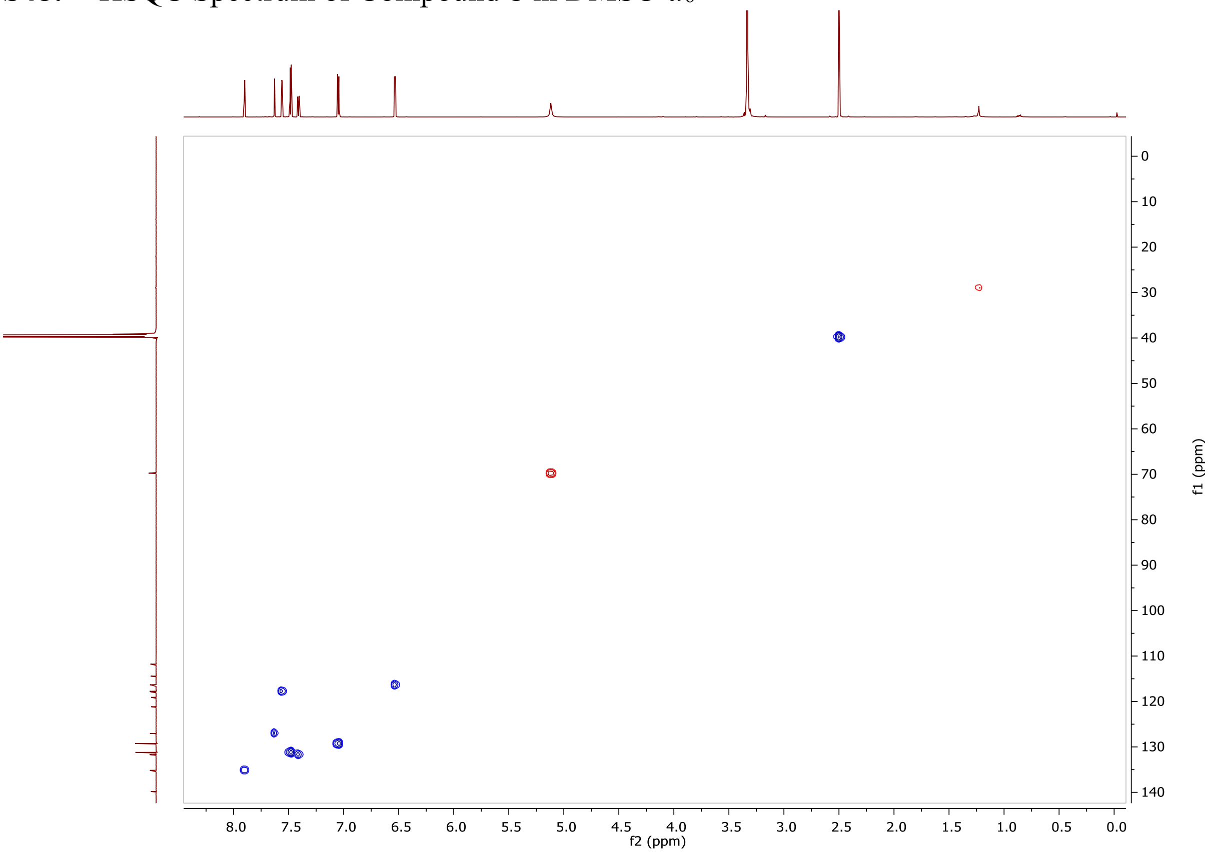

S49: HMBC Spectrum of Compound **8** in DMSO-d_6

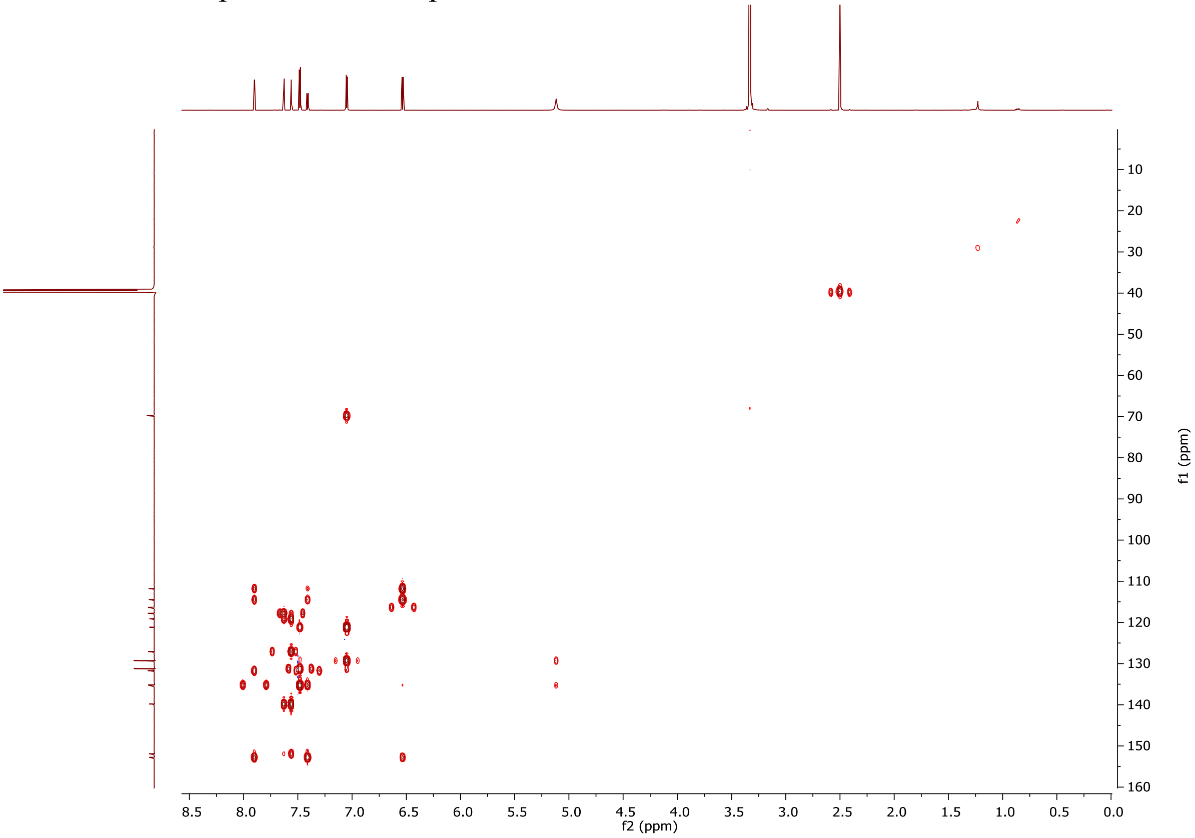

S50: ROESY Spectrum of Compound **8** in DMSO-d_6

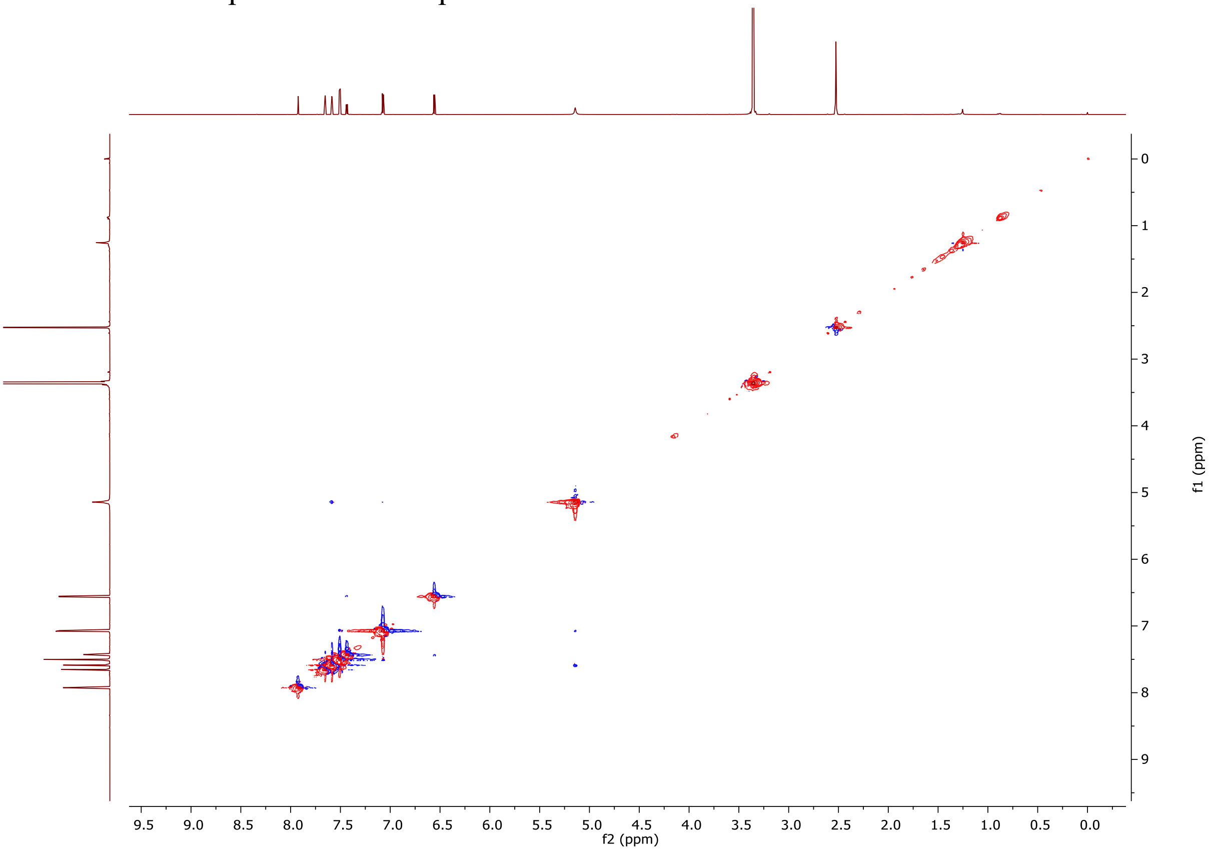

S51: ^{1}H NMR (800 MHz) Spectrum of Compound **9** in DMSO-d_6

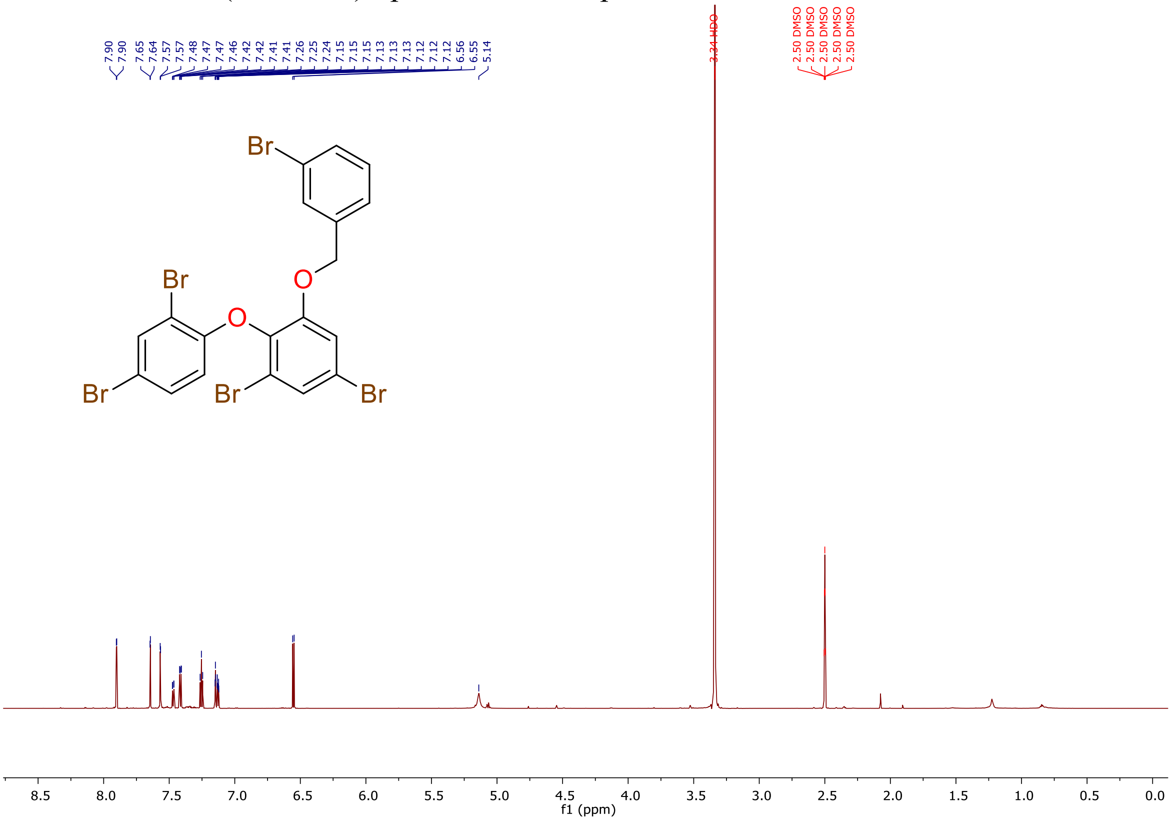

S52: ^{13}C NMR (200 MHz) Spectrum of Compound **9** in DMSO-d_6

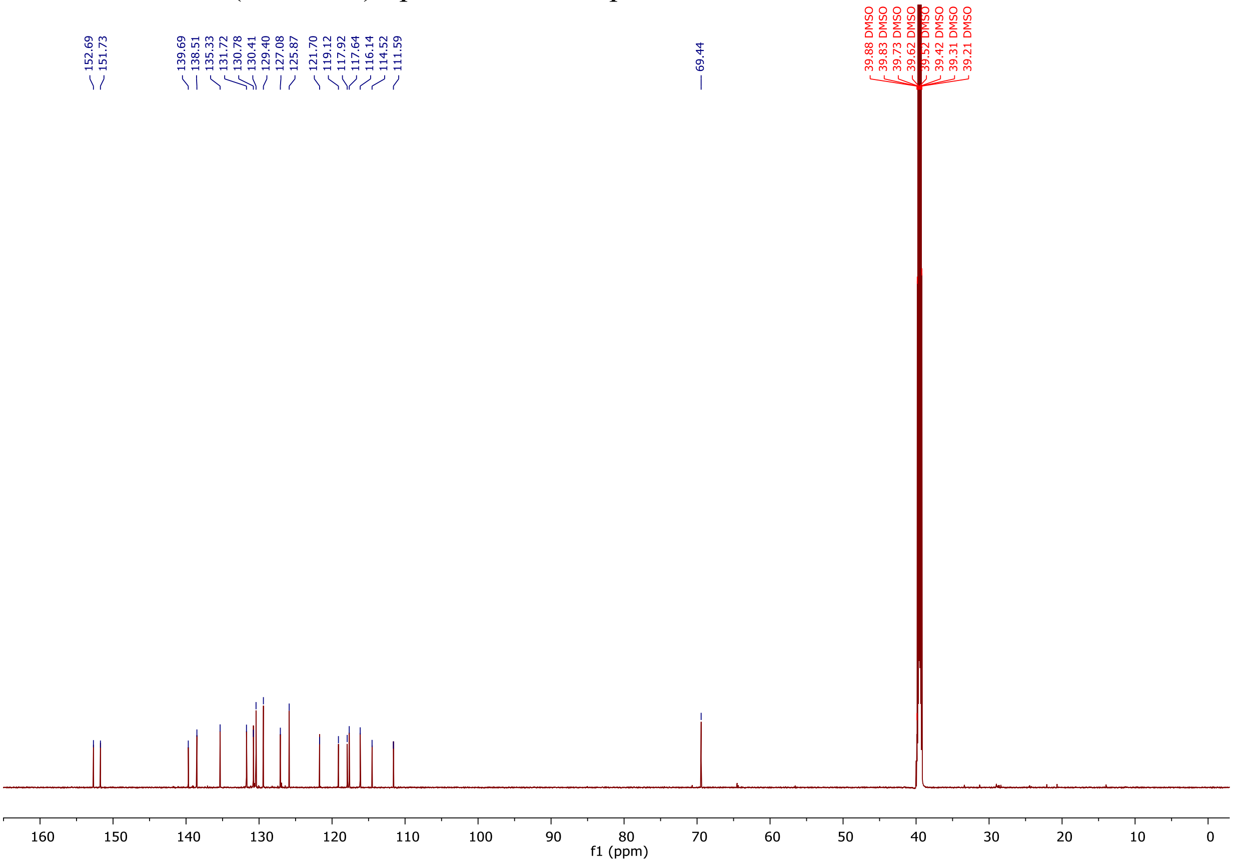

S53: COSY Spectrum of Compound **9** in DMSO-*d*6

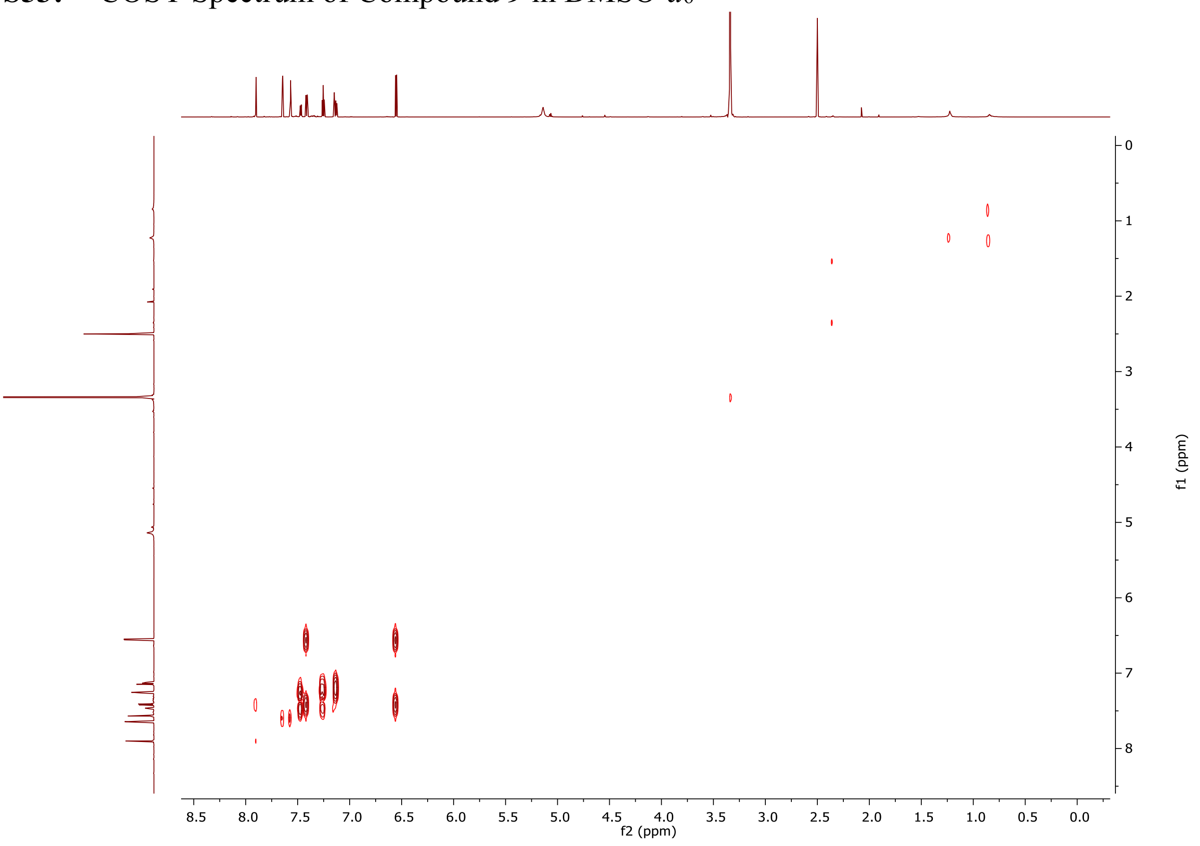

S54: HSQC Spectrum of Compound **9** in DMSO-*d*6

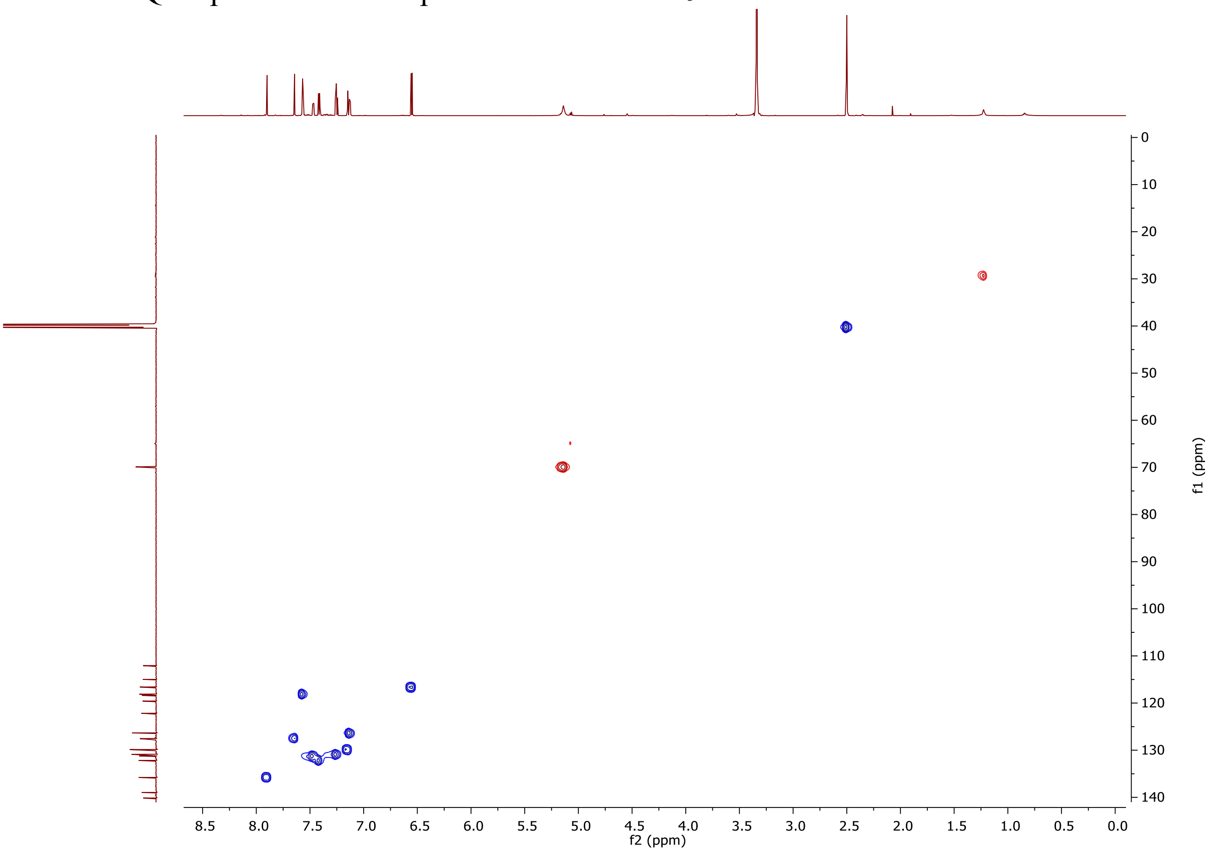

S55: HMBC Spectrum of Compound **9** in DMSO-*d*6

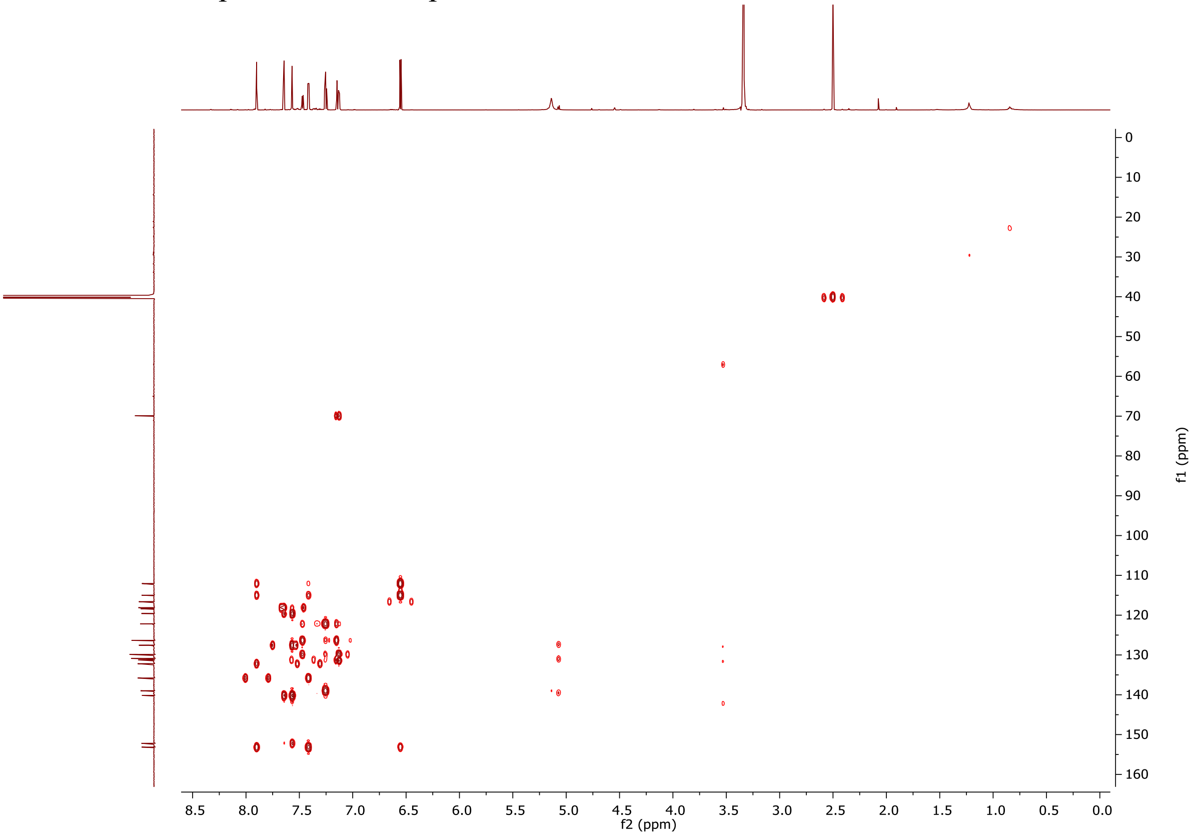

S56: ROESY Spectrum of Compound **9** in DMSO-*d*6

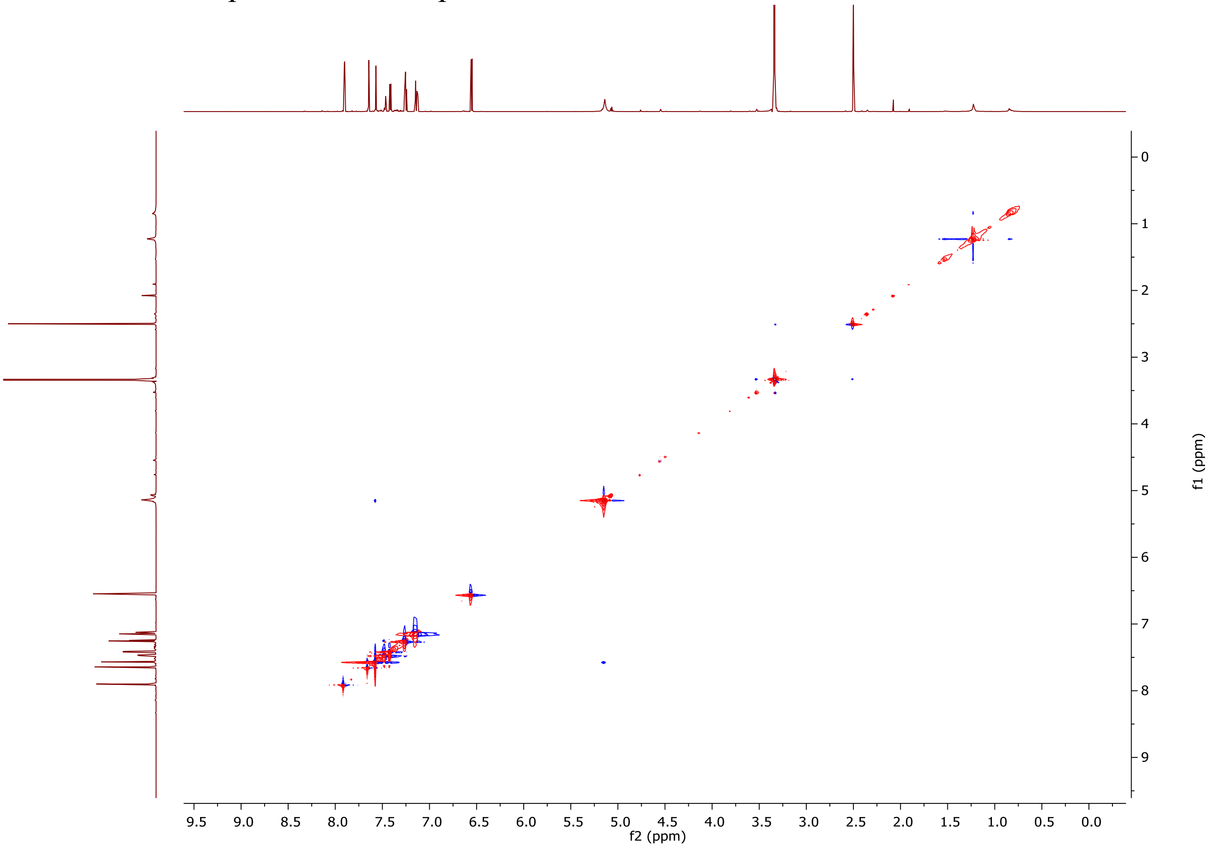

S57: ^{1}H NMR (800 MHz) Spectrum of Compound **10** in DMSO-d_6

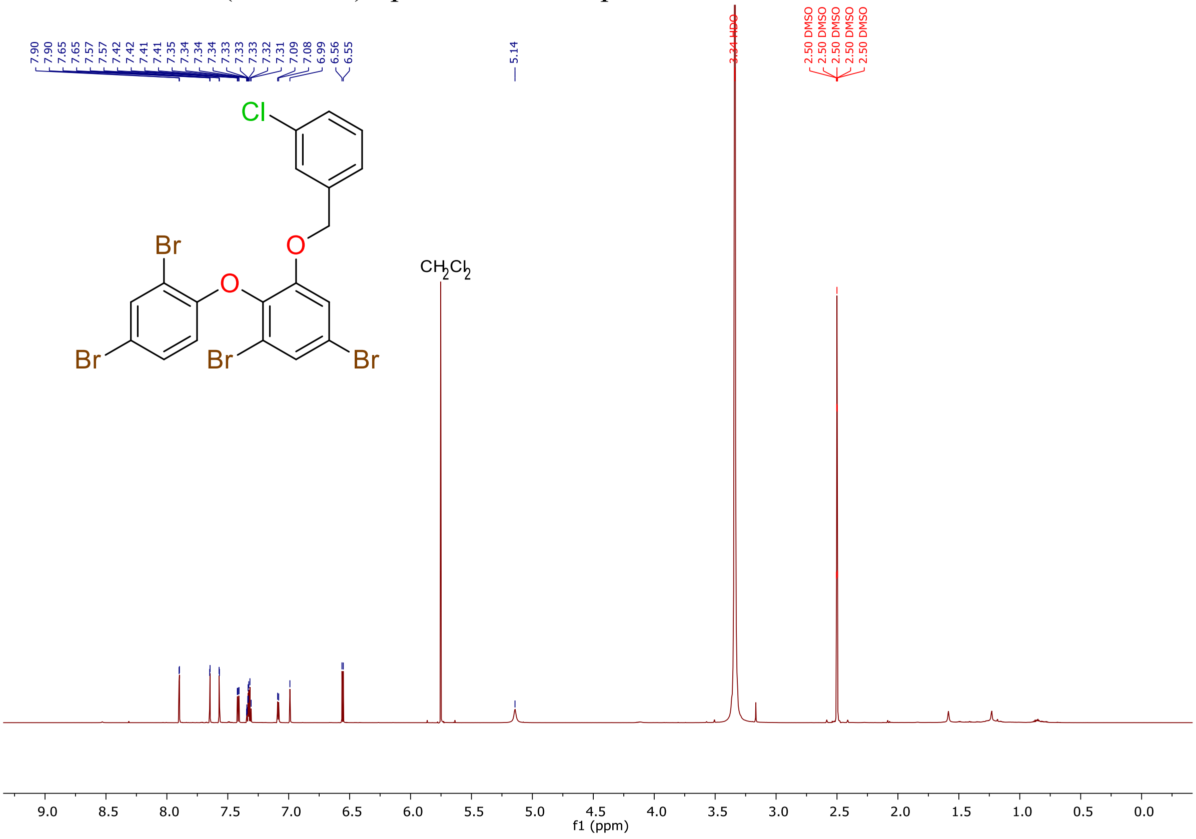

S58: ^{13}C NMR (200 MHz) Spectrum of Compound **10** in DMSO-d_6

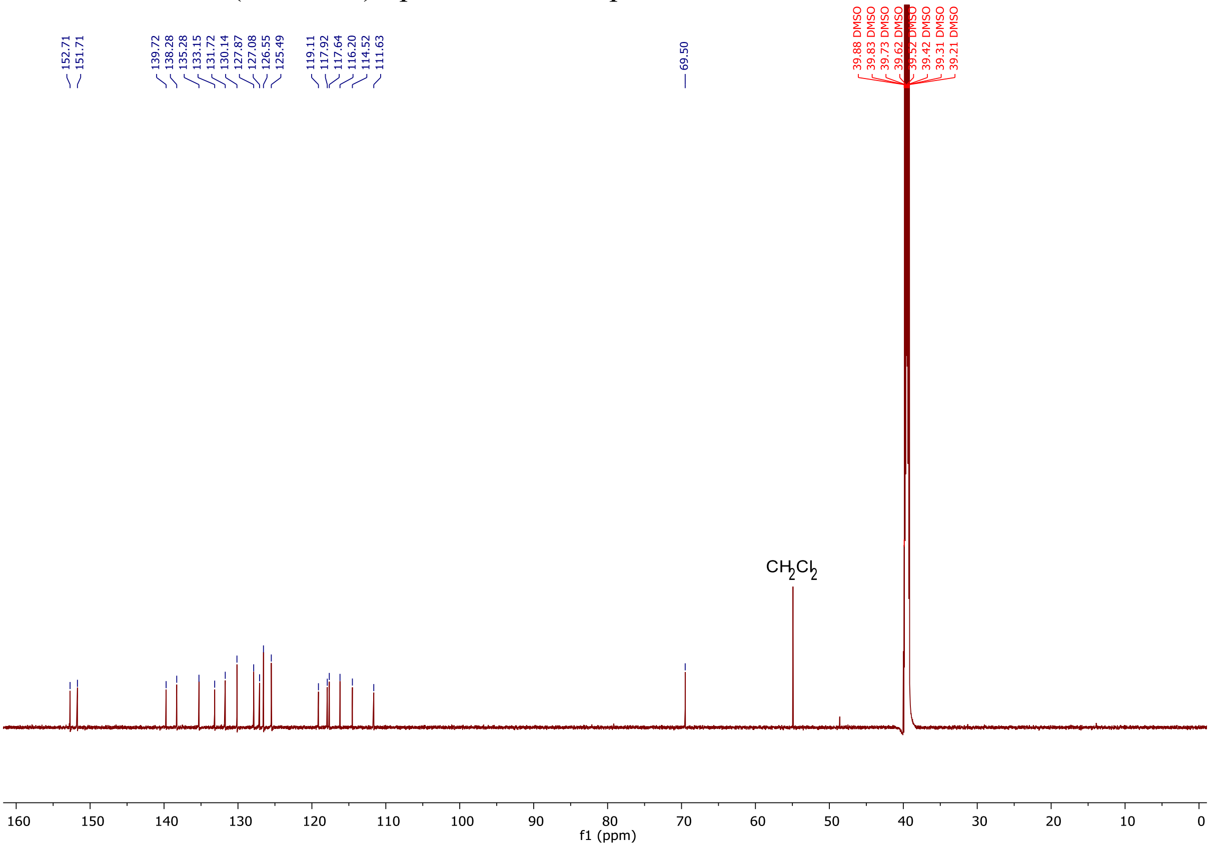

S59: COSY Spectrum of Compound **10** in DMSO-d_6

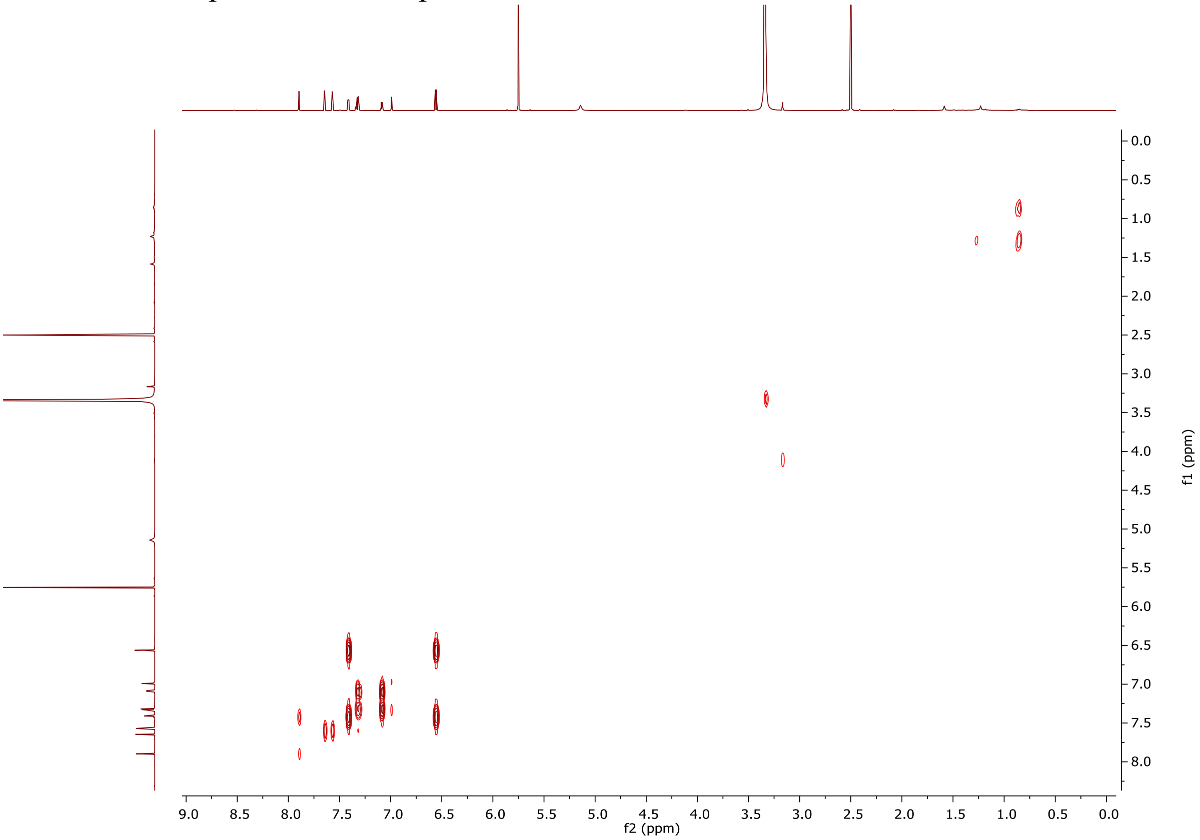

S60: HSQC Spectrum of Compound **10** in DMSO-d_6

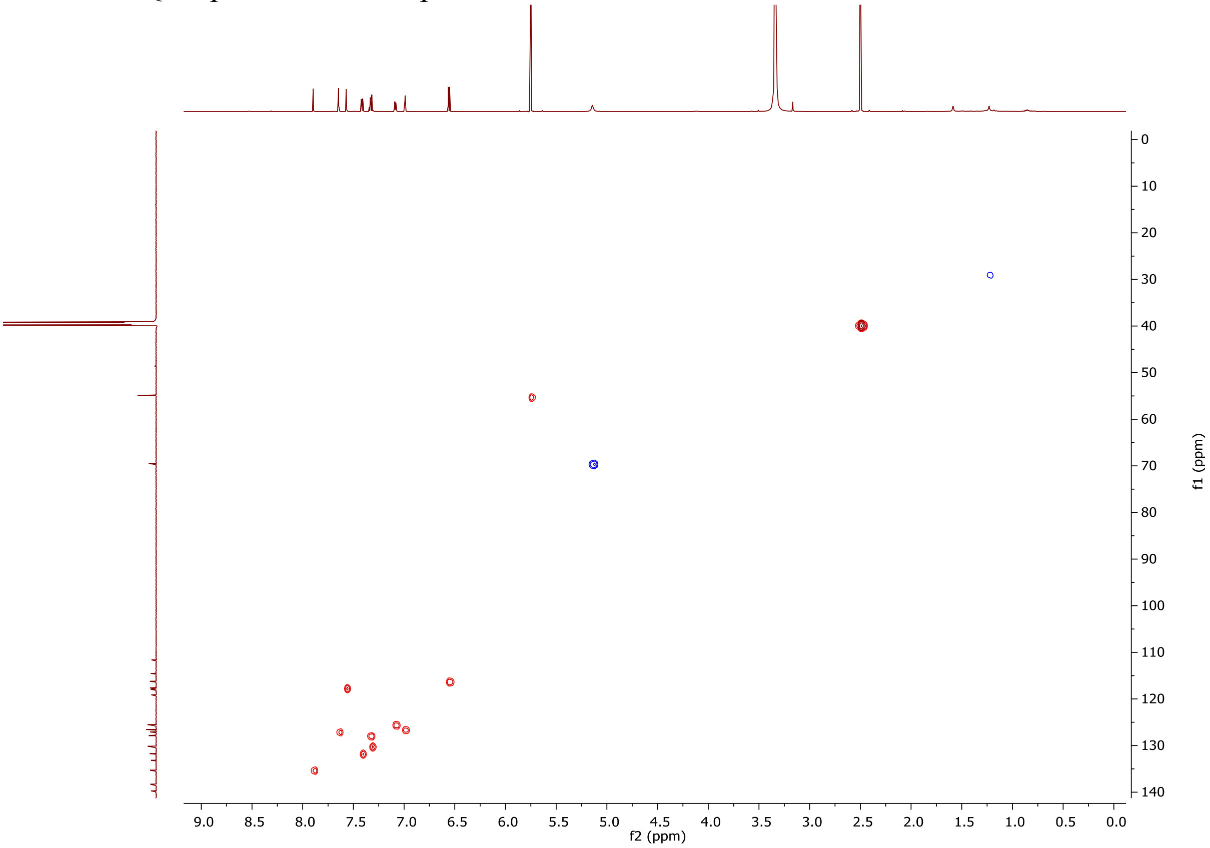

S61: HMBC Spectrum of Compound **10** in DMSO-d_6

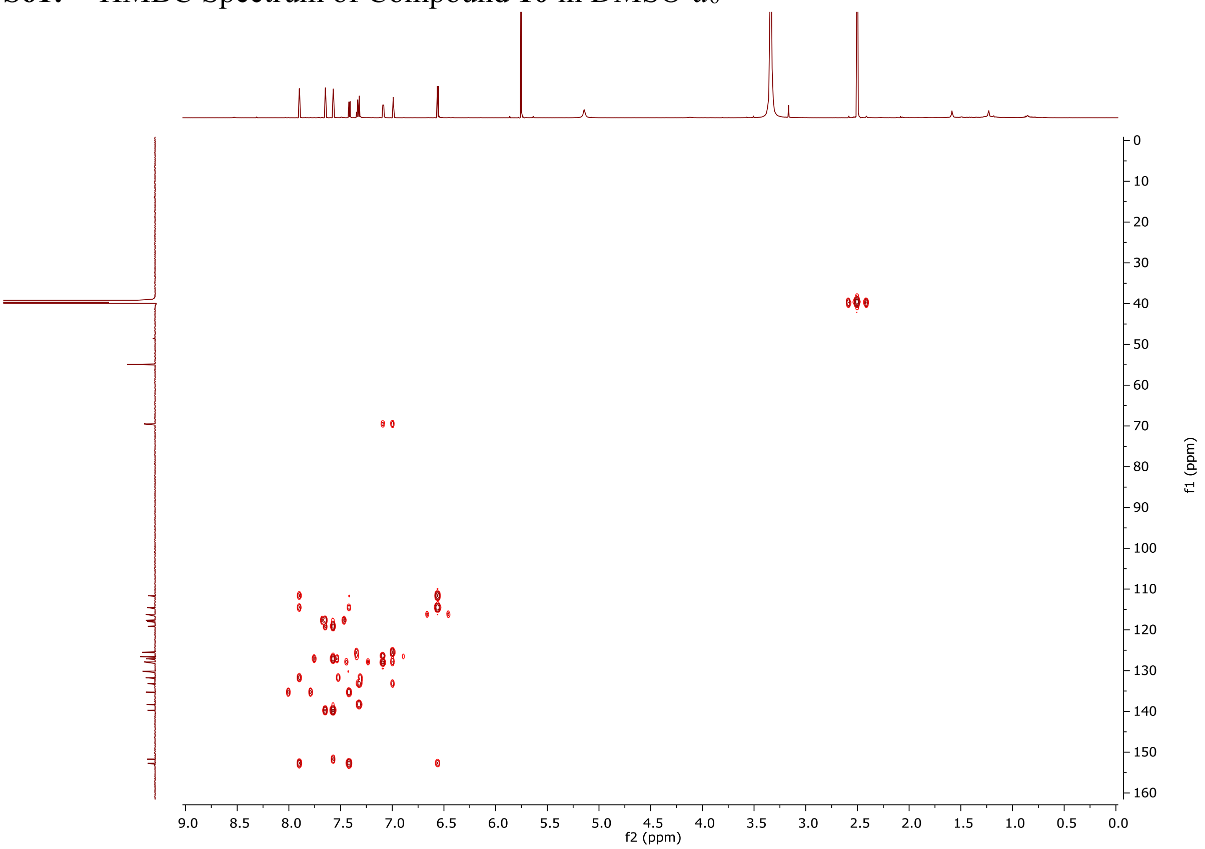

S62: ROESY Spectrum of Compound **10** in DMSO-d_6

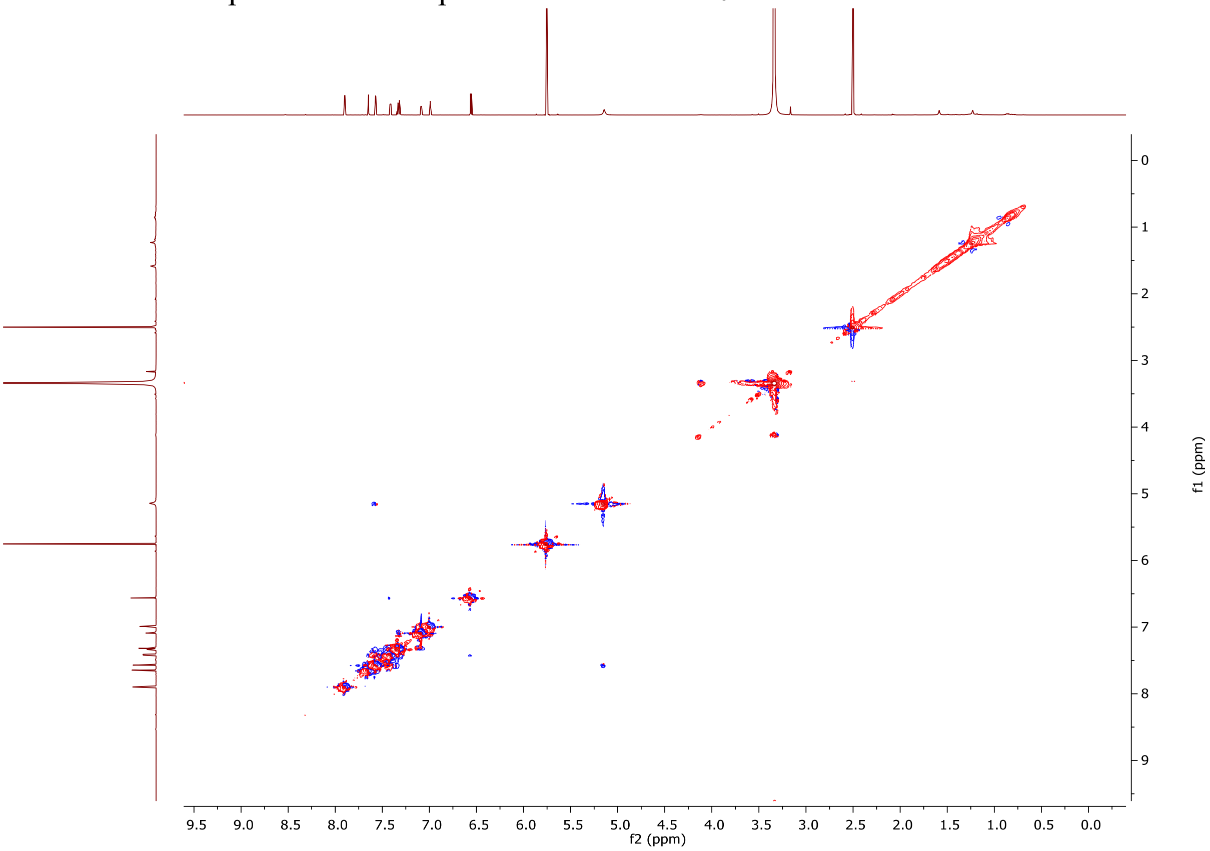

S63: ^{1}H NMR (800 MHz) Spectrum of Compound **11** in DMSO-d_6

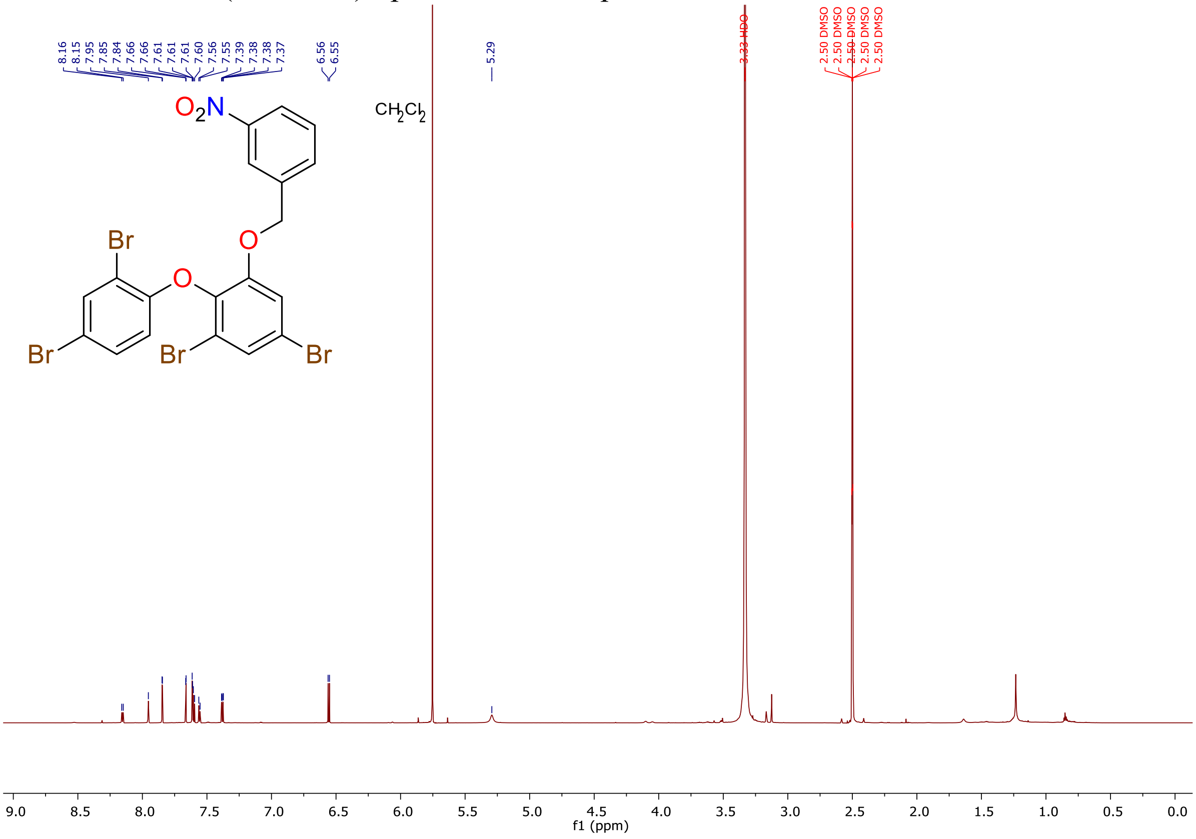

S64: ^{13}C NMR (200 MHz) Spectrum of Compound **11** in DMSO-d_6

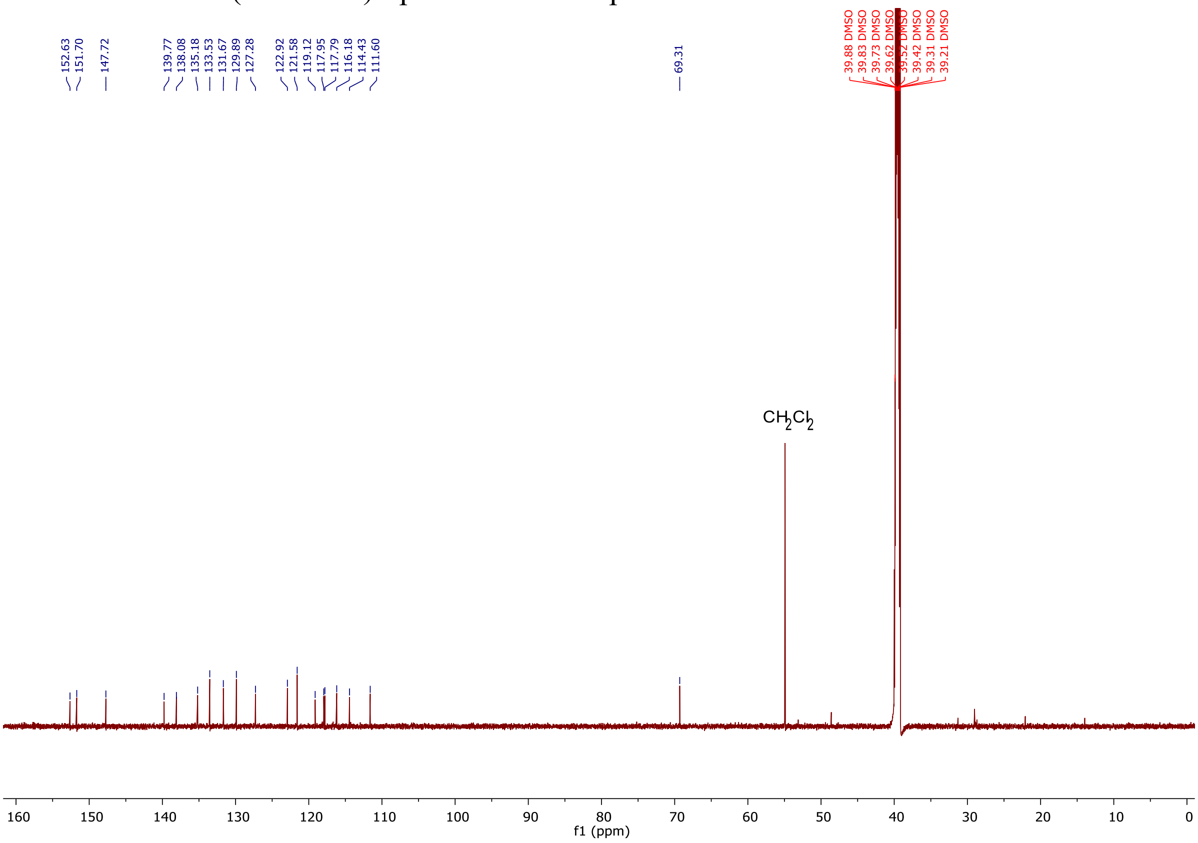

S65: COSY Spectrum of Compound **11** in DMSO-d_6

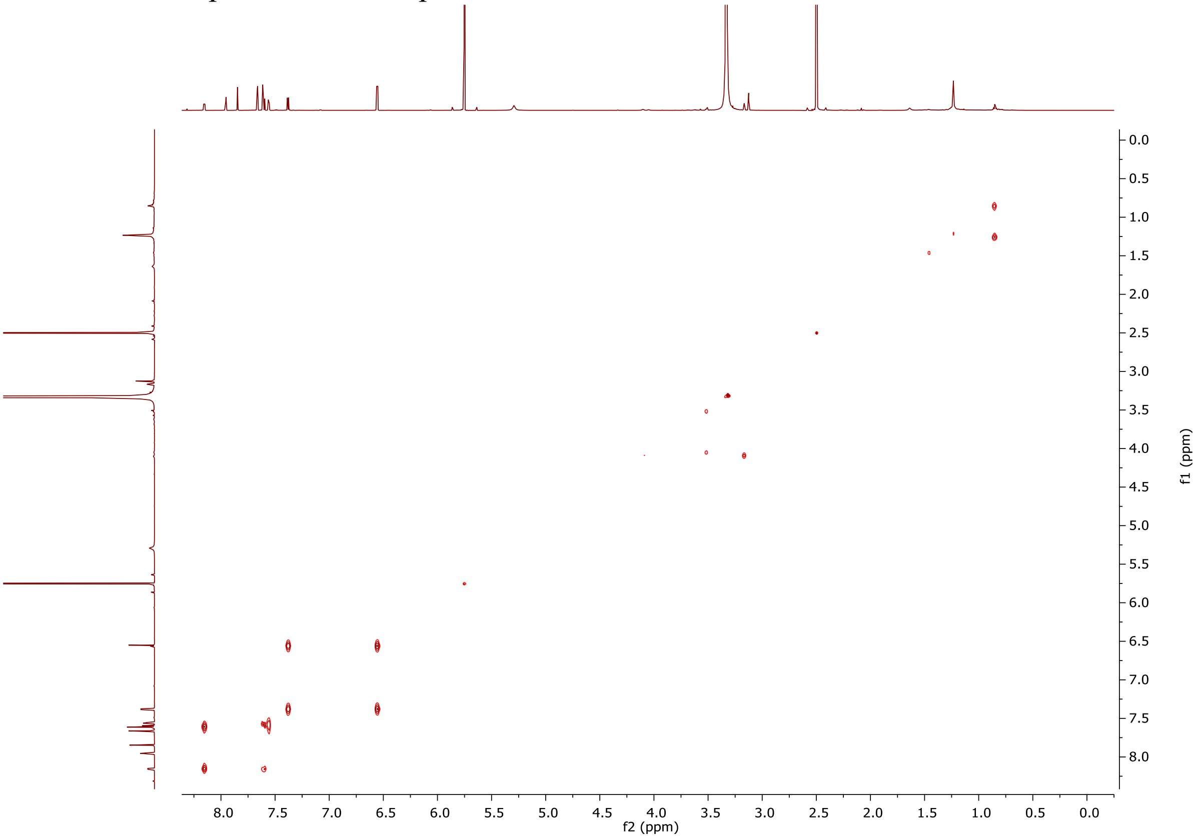

S66: HSQC Spectrum of Compound **11** in DMSO-d_6

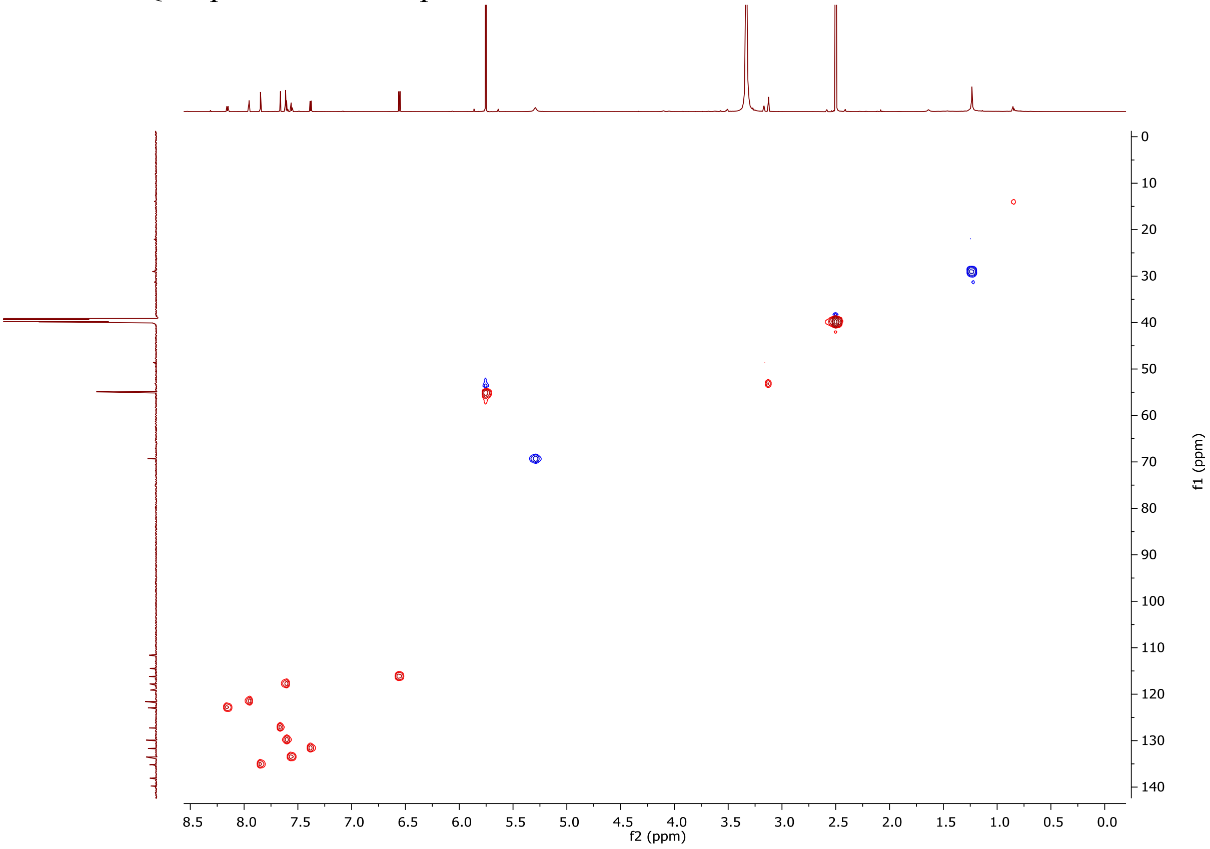

S67: HMBC Spectrum of Compound **11** in DMSO-*d*6

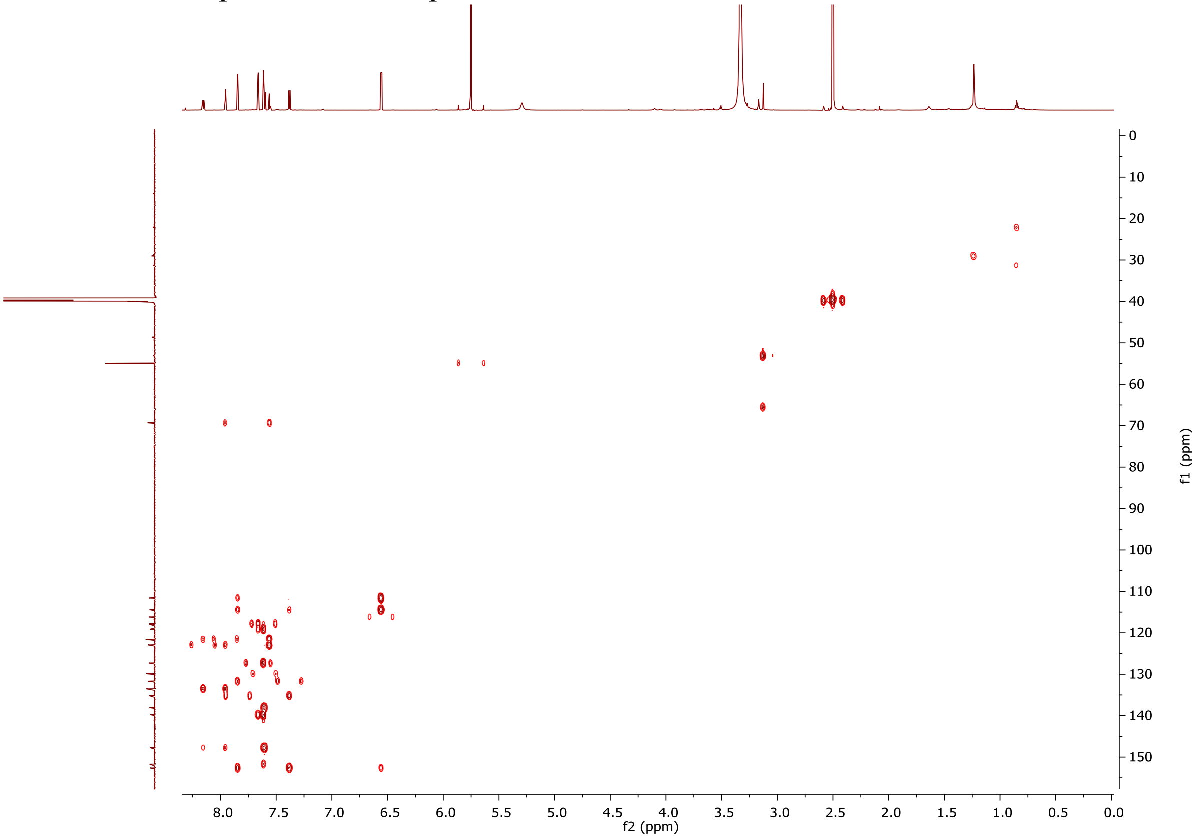

S68: ROESY Spectrum of Compound **11** in DMSO-*d*6

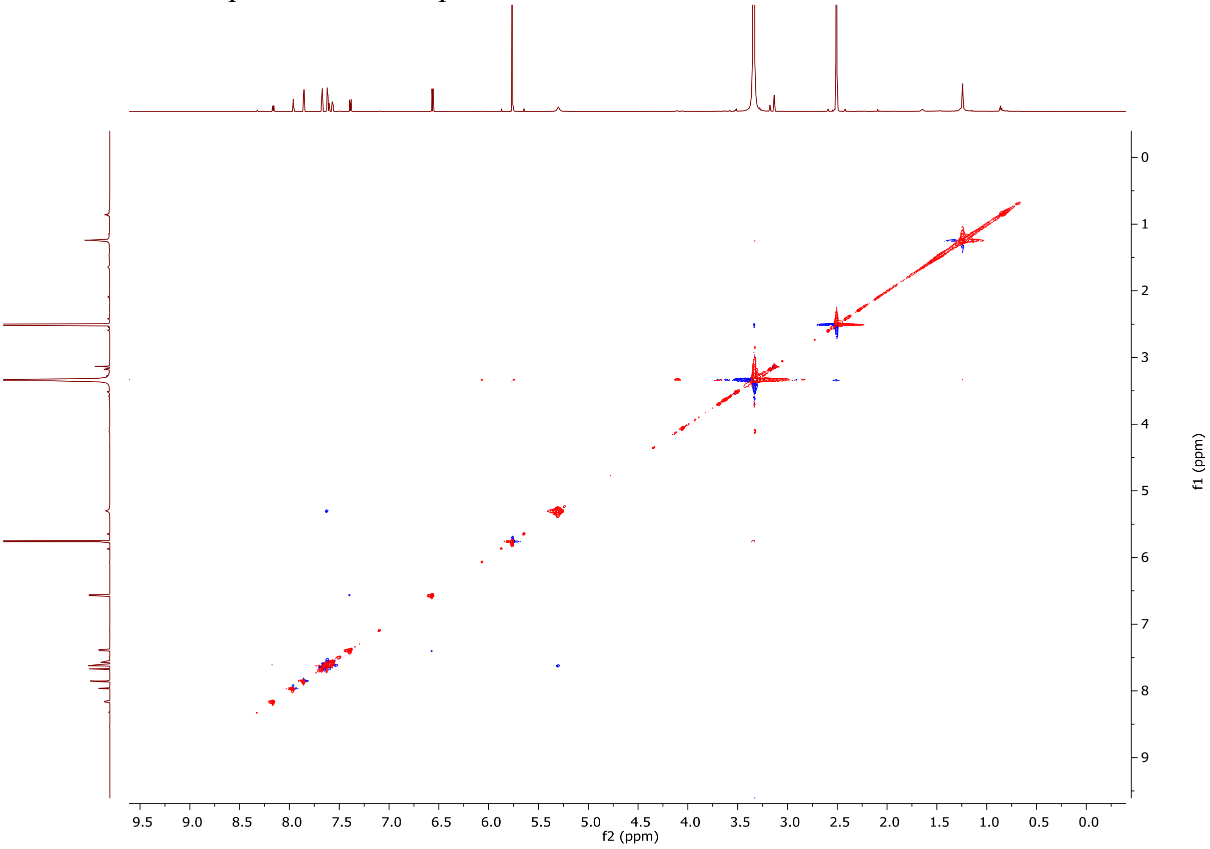

S69: ^{1}H NMR (800 MHz) Spectrum of Compound **12** in DMSO-d_6

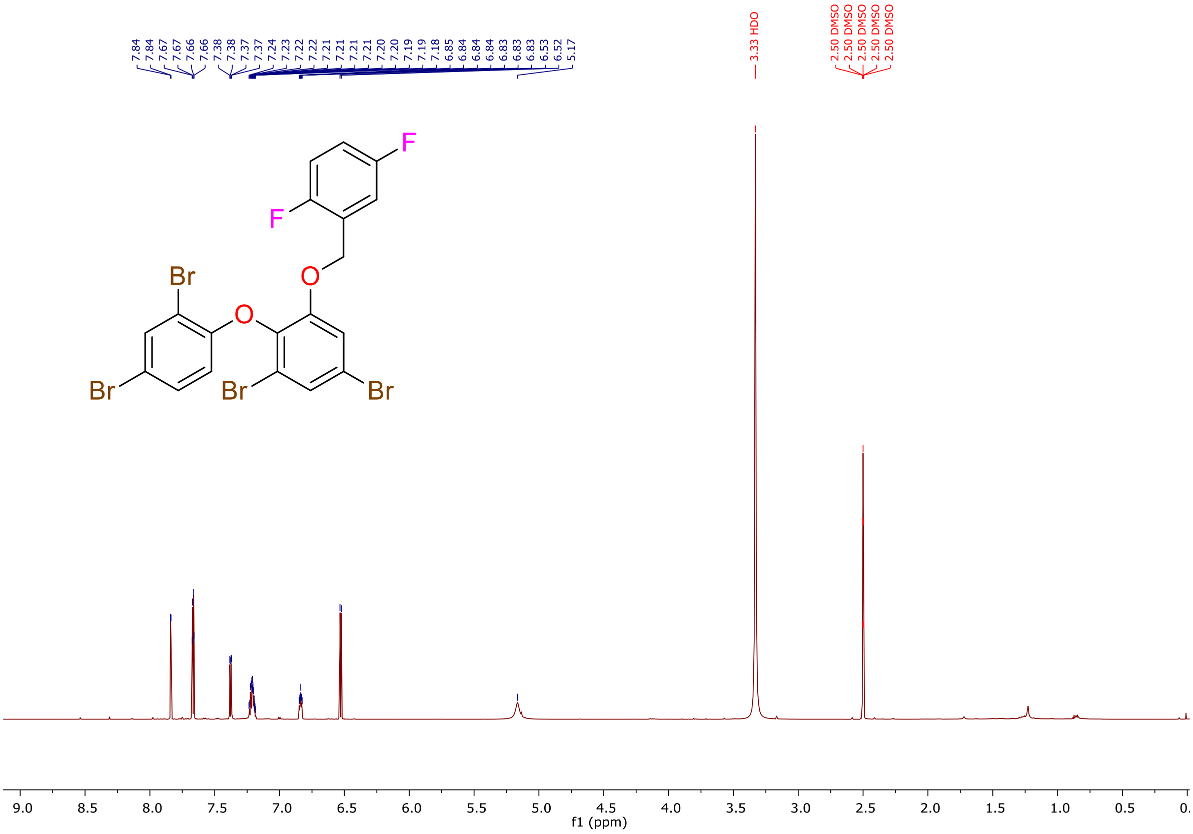

S70: ^{13}C NMR (200 MHz) Spectrum of Compound **12** in DMSO-d_6

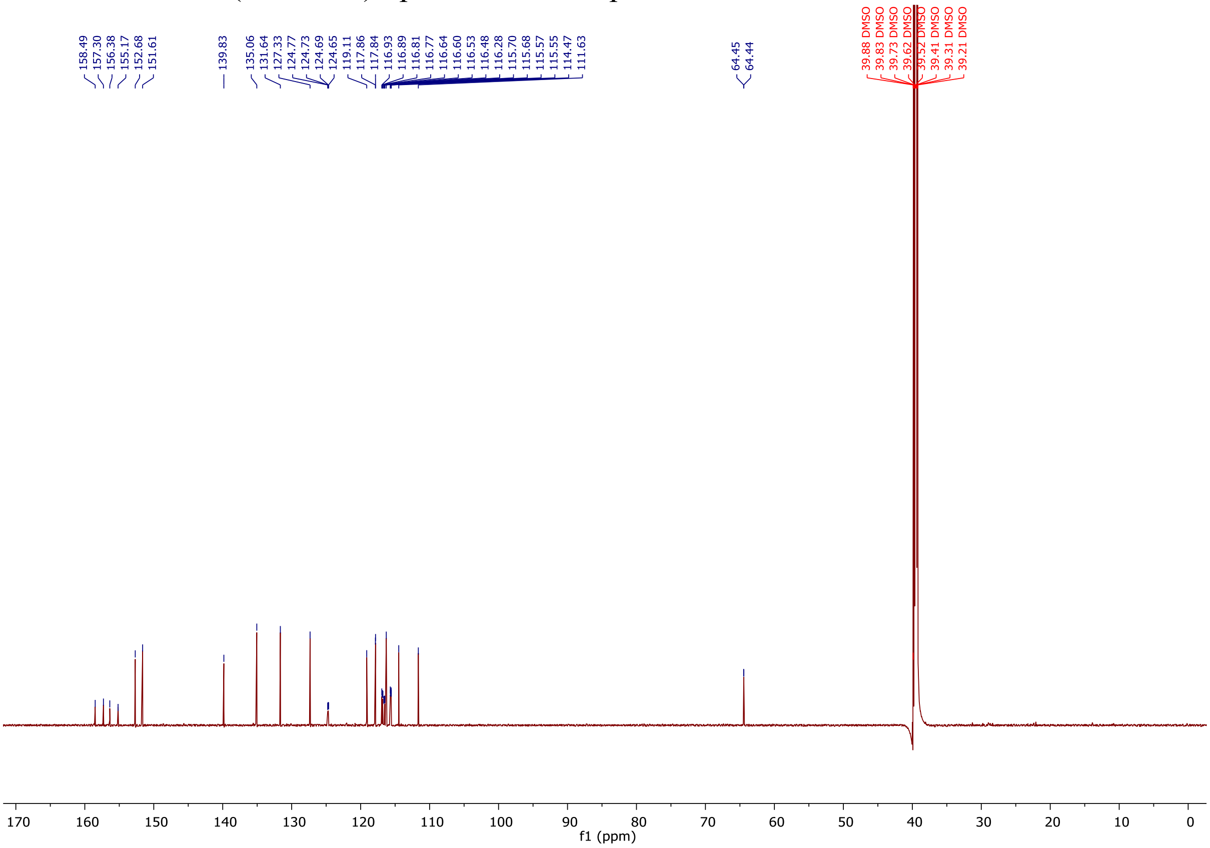

S71: COSY Spectrum of Compound **12** in DMSO-d_6

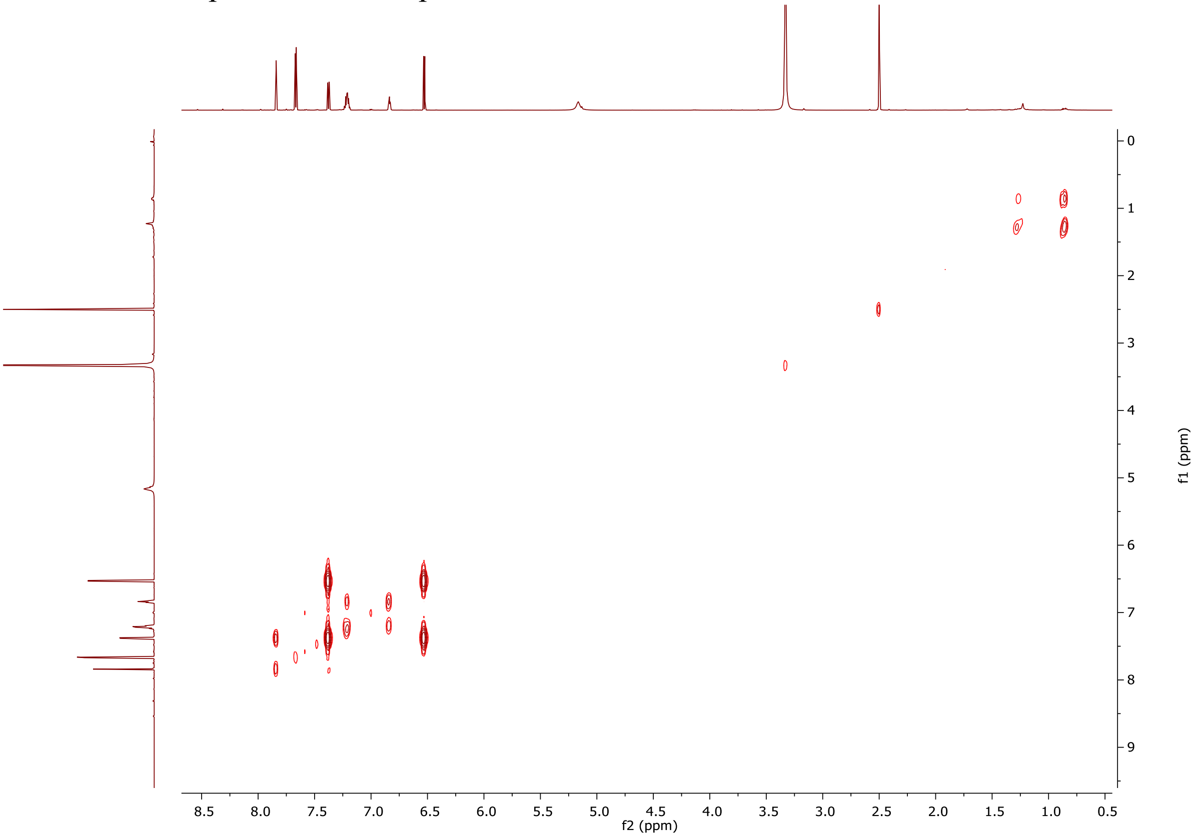

S72: HSQC Spectrum of Compound **12** in DMSO-d_6

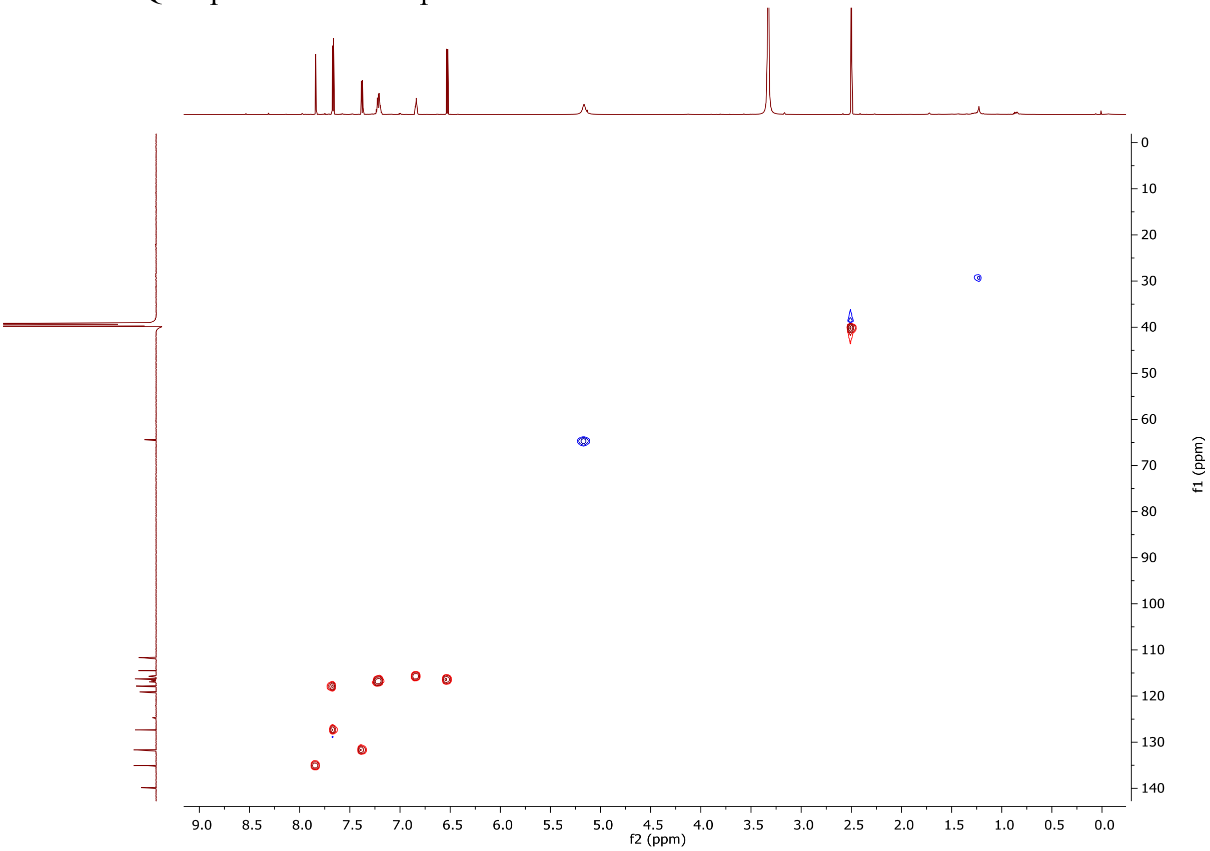

S73: HMBC Spectrum of Compound **12** in DMSO-*d*6

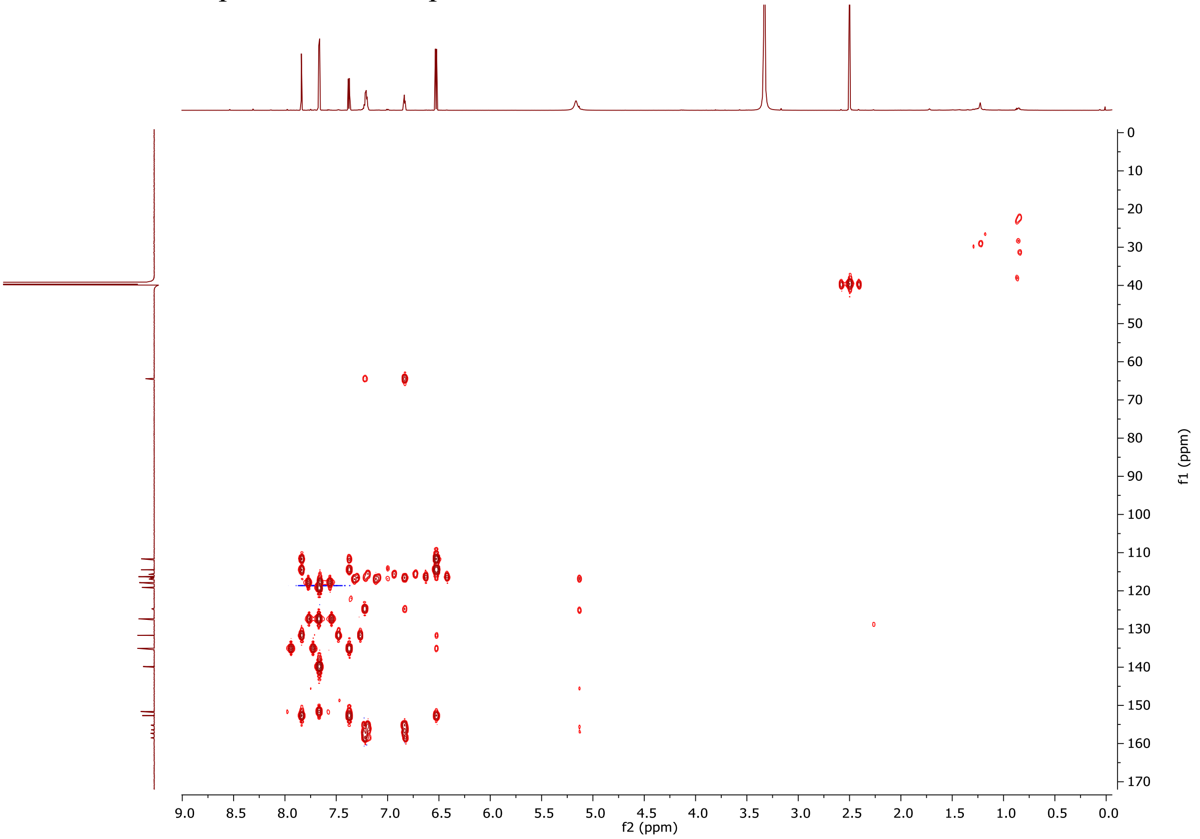

S74: ROESY Spectrum of Compound **12** in DMSO-*d*6

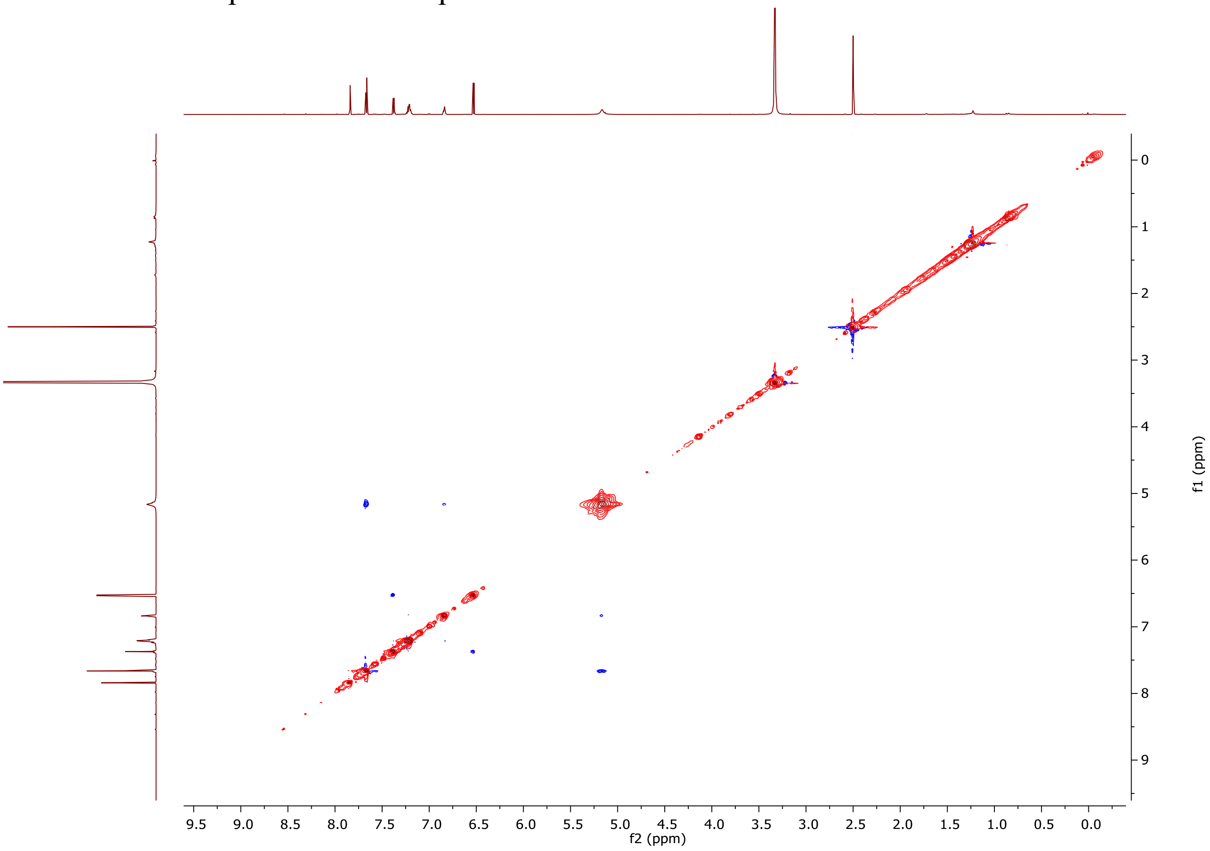

S75: ^{1}H NMR (800 MHz) Spectrum of Compound **13** in DMSO-d_6

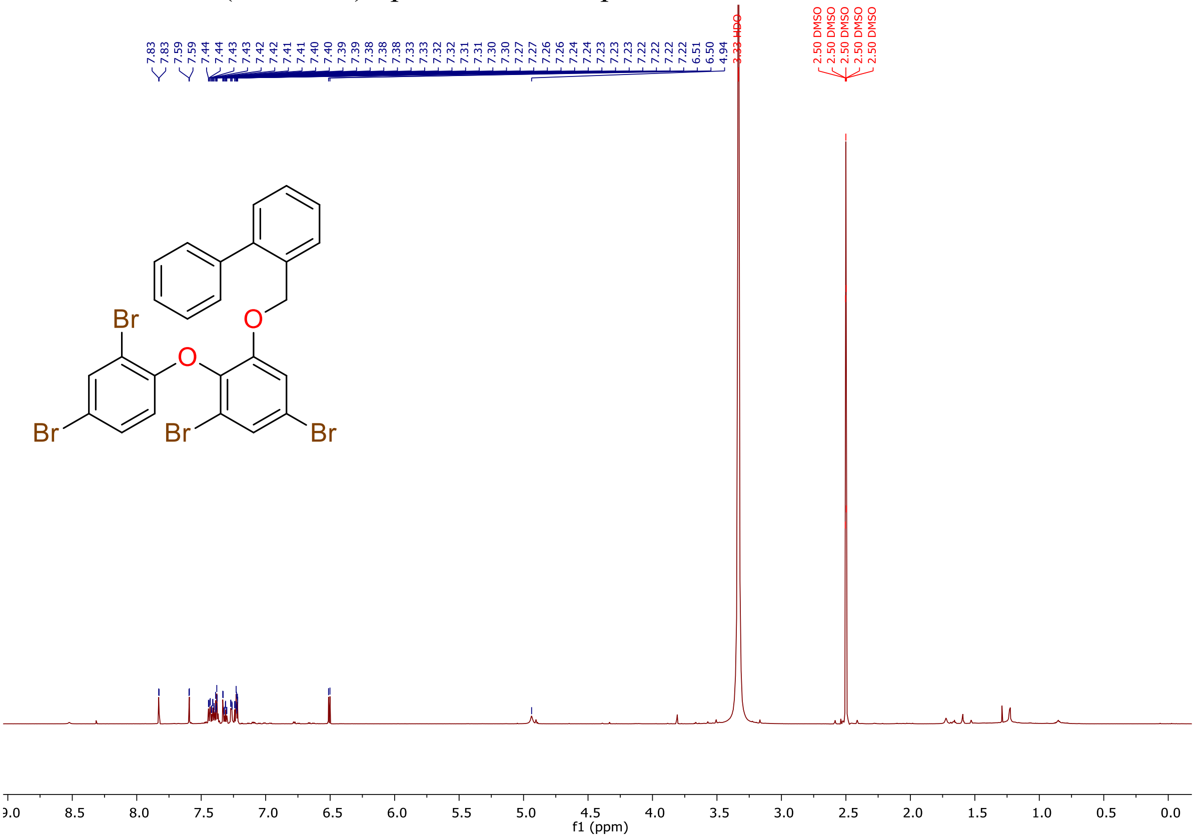

S76: ^{13}C NMR (200 MHz) Spectrum of Compound **13** in DMSO-d_6

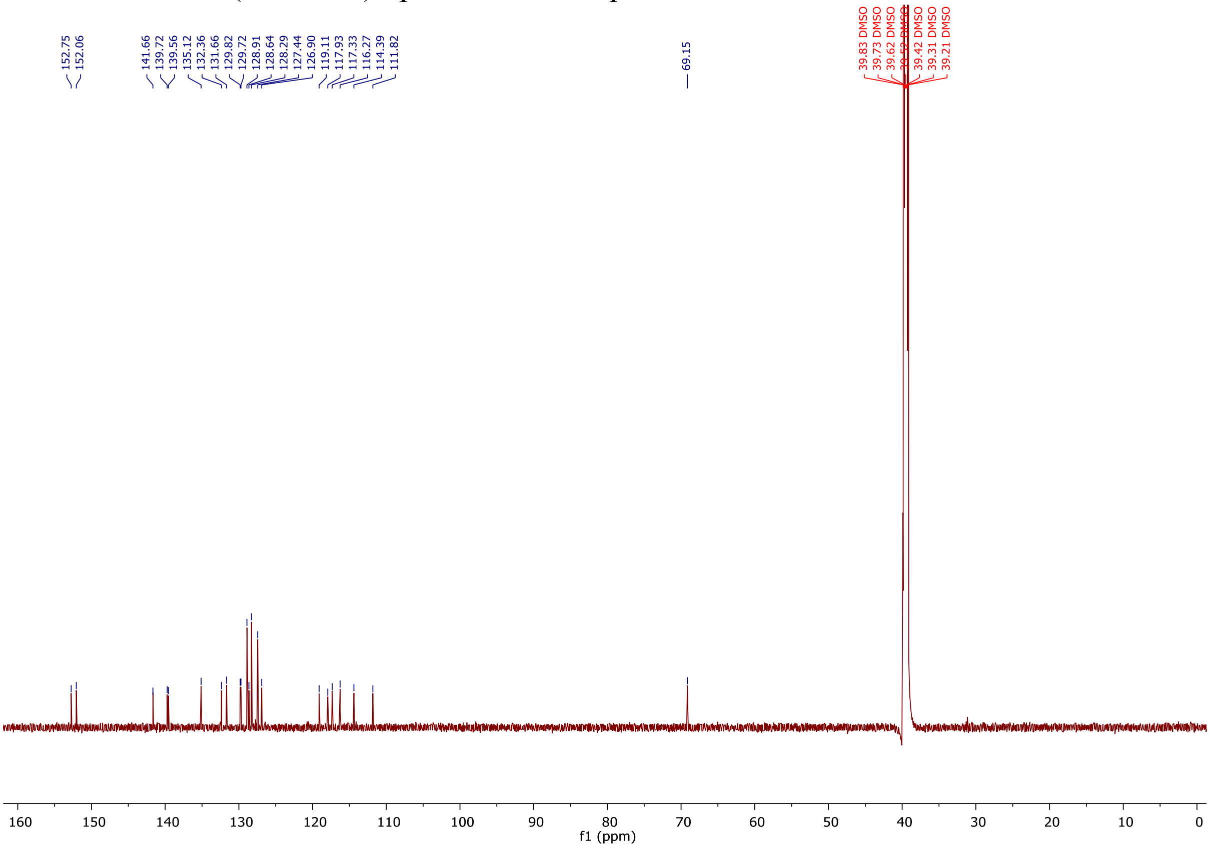

S77: COSY Spectrum of Compound **13** in DMSO-d_6

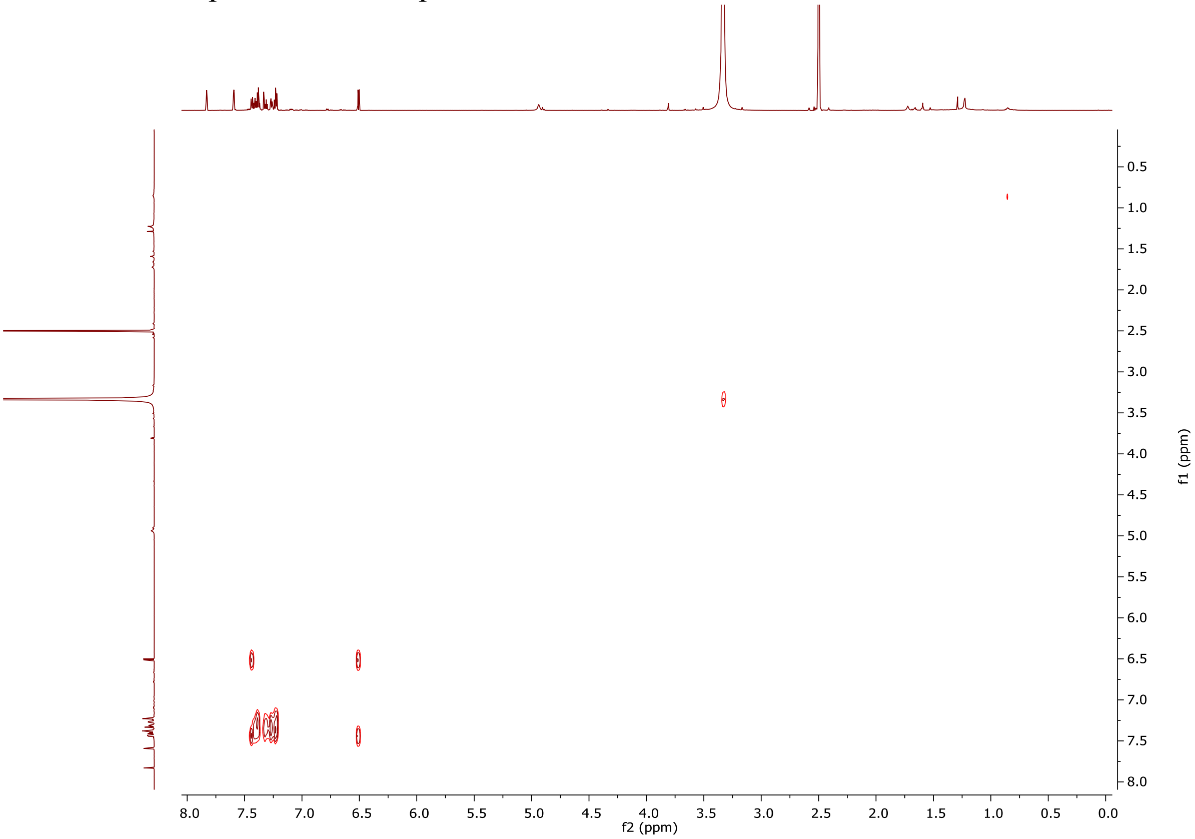

S78: HSQC Spectrum of Compound **13** in DMSO-d_6

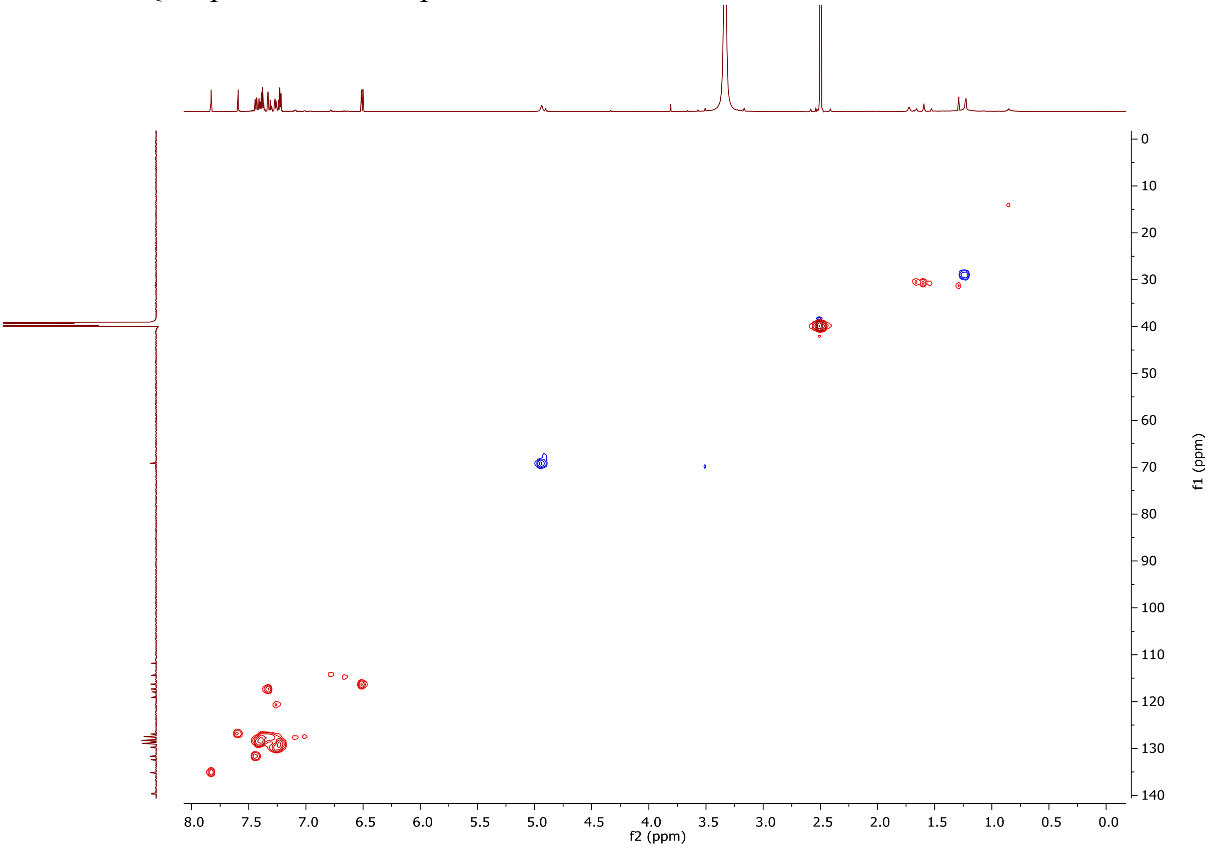

S79: HMBC Spectrum of Compound **13** in DMSO-d_6

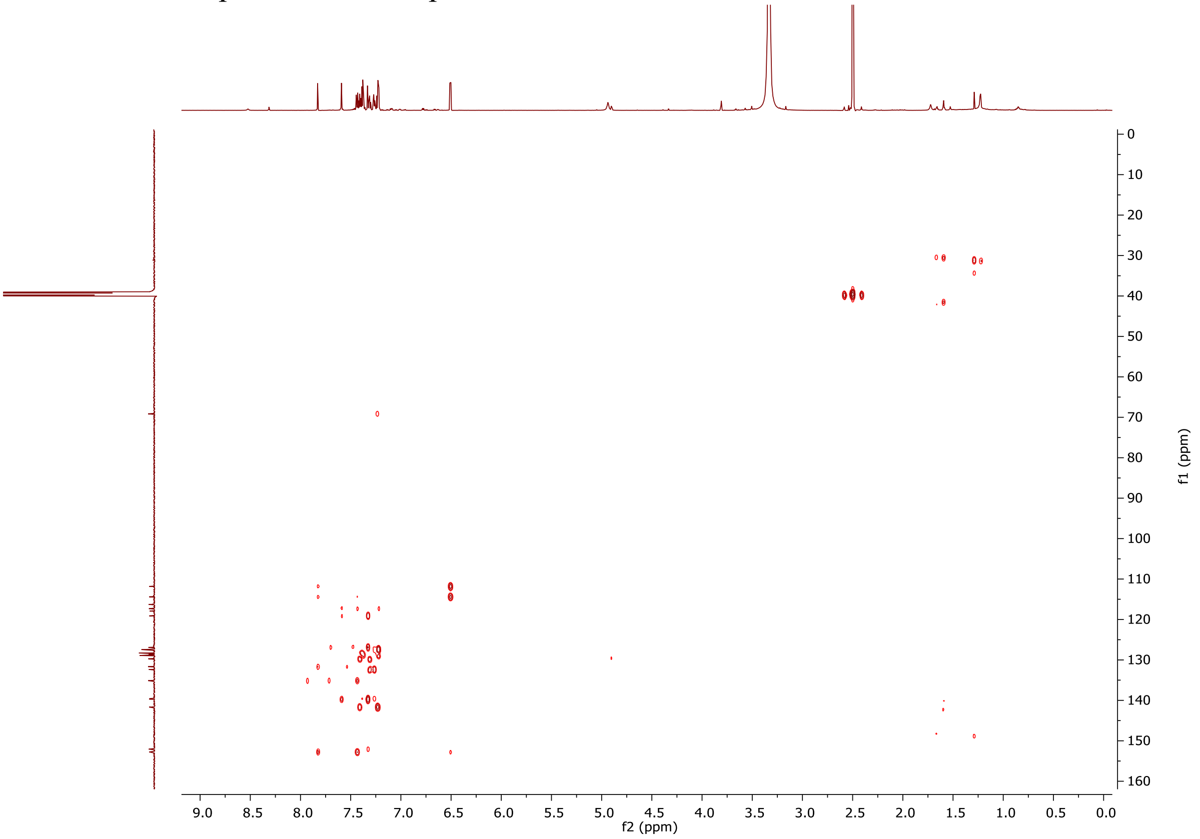

S80: ROESY Spectrum of Compound **13** in DMSO-d_6

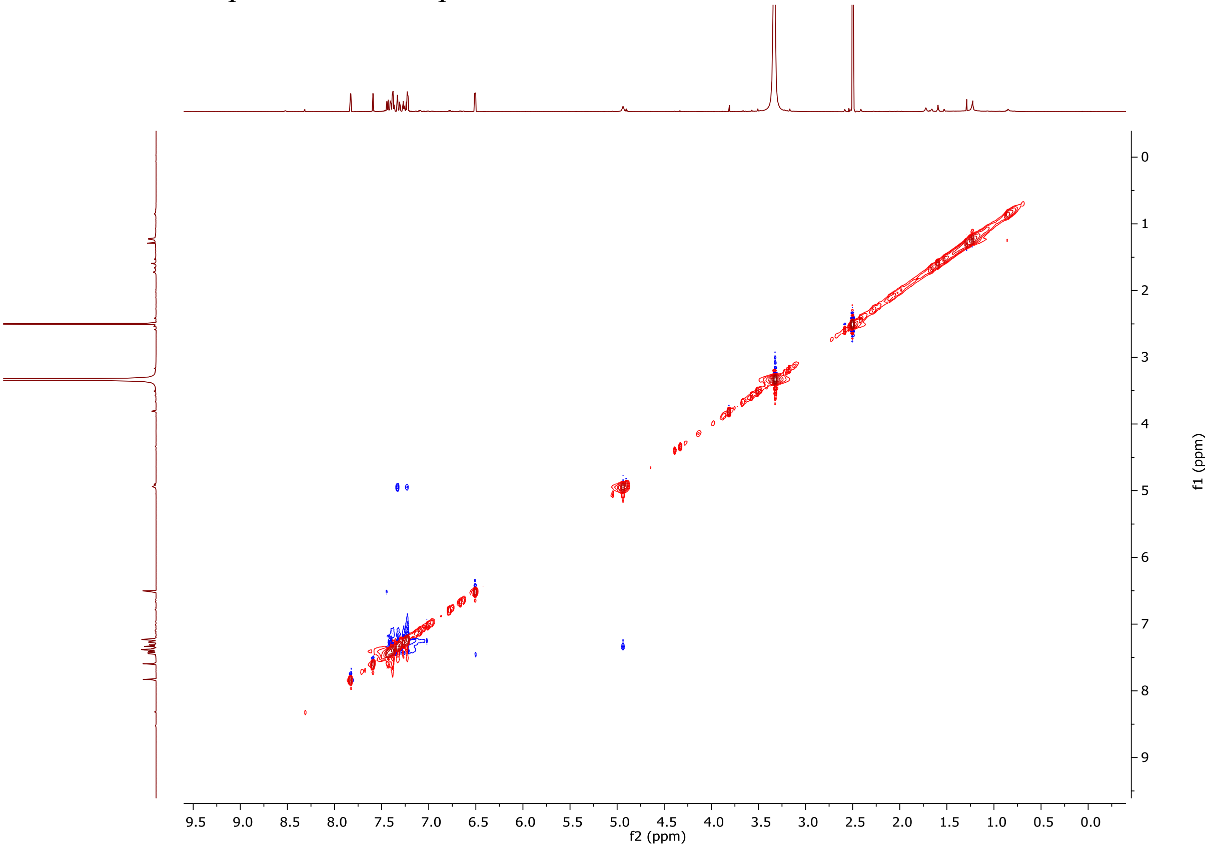

S81: HRESIMS Data of Compound **1**

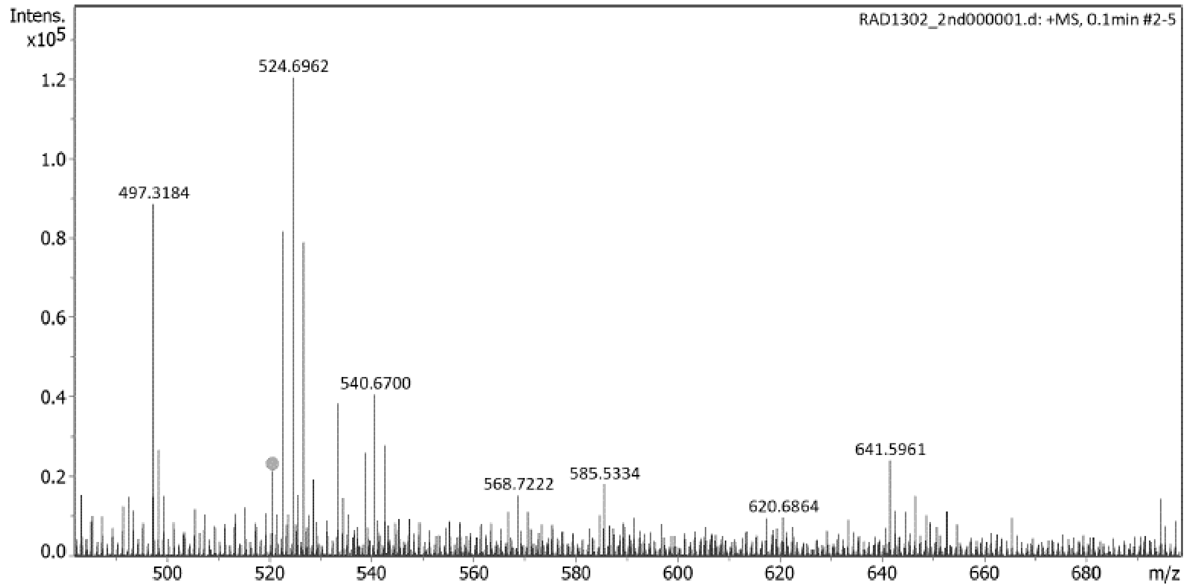

Meas. m/z	#	Ion Formula	m/z	err [ppm]	mSigma	# mSigma	Score	rdb	e⁻ Conf	N-Rule
520.7002	1	C12H6Br4NaO2	520.6994	-1.7	3.1	1	100.00	16.0	even	ok

S82: HRESIMS Data of Compound **2**

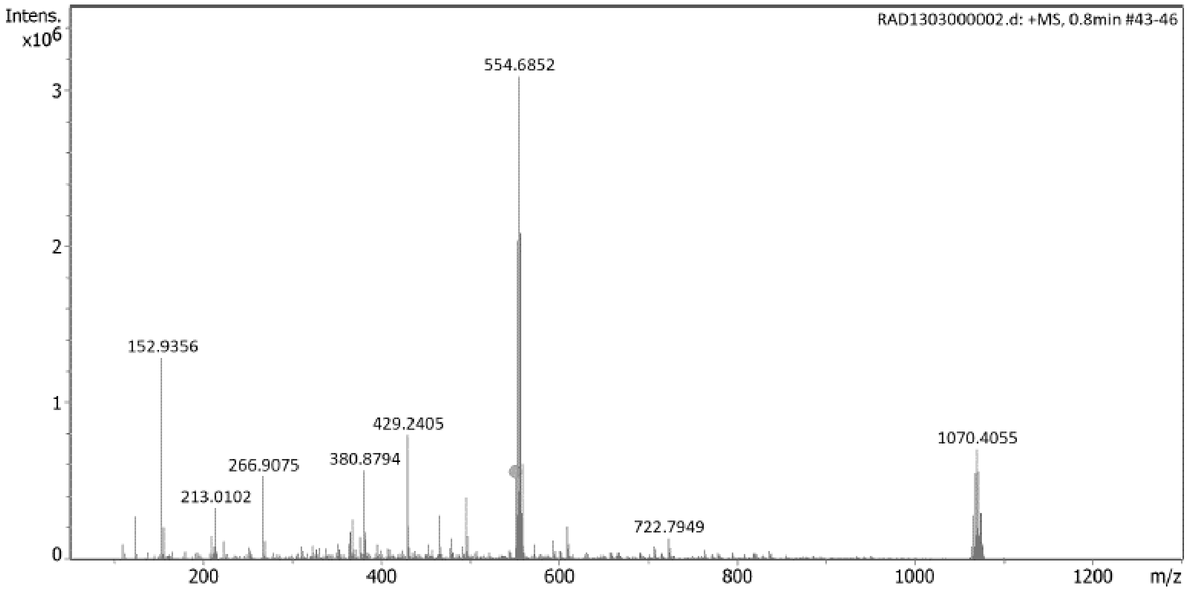

Meas. m/z	#	Ion Formula	m/z	err [ppm]	mSigma	# mSigma	Score	rdb	e⁻ Conf	N-Rule
550.6893	1	C13H8Br4KO2	550.6889	-0.6	6.5	1	100.00	16.0	even	ok

S83: HRESIMS Data of Compound **3**

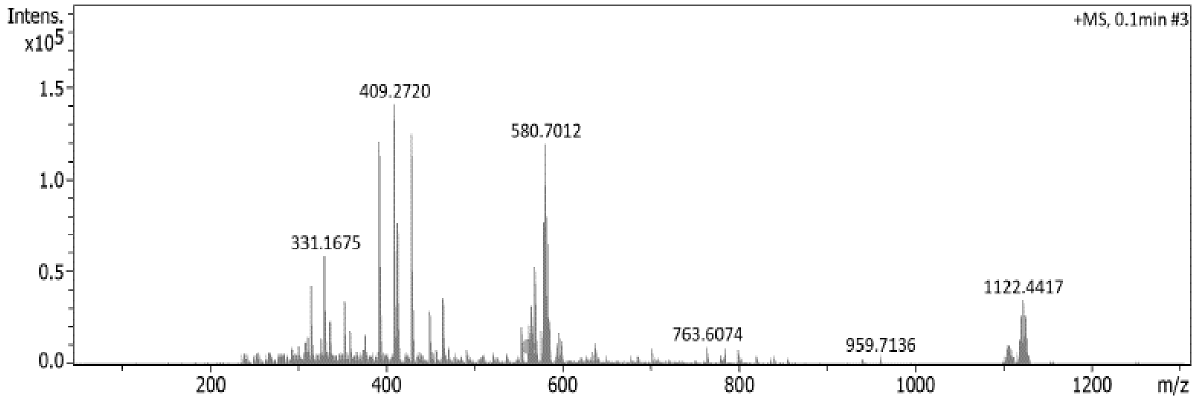

Meas. m/z	#	Ion Formula	m/z	err [ppm]	mSigma	# mSigma	Score	rdb	e⁻ Conf	N-Rule
560.7306	1	C15H10Br4NaO2	560.7307	0.0	5.7	1	100.00	17.0	even	ok

S84: HRESIMS Data of Compound **4**

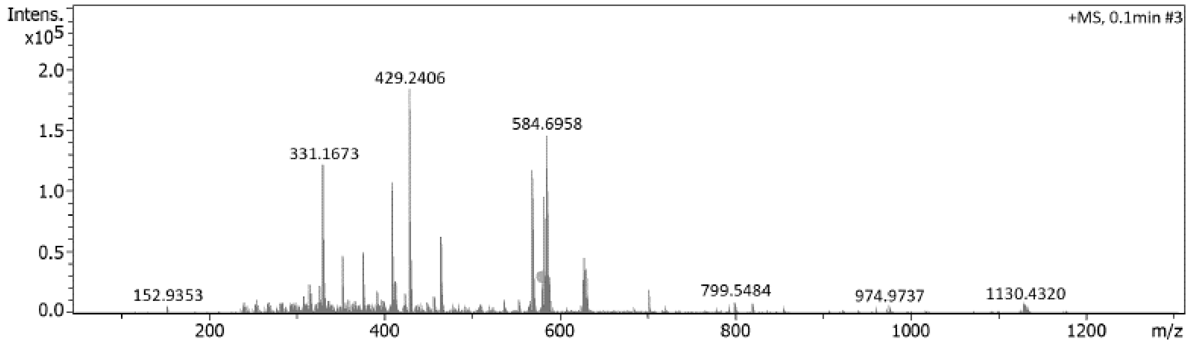

Meas. m/z	#	Ion Formula	m/z	err [ppm]	mSigma	# mSigma	Score	rdb	e⁻ Conf	N-Rule
580.7000	1	C14H10Br4KO3	580.6995	-0.8	3.1	1	100.00	16.0	even	ok

S85: HRESIMS Data of Compound **5**

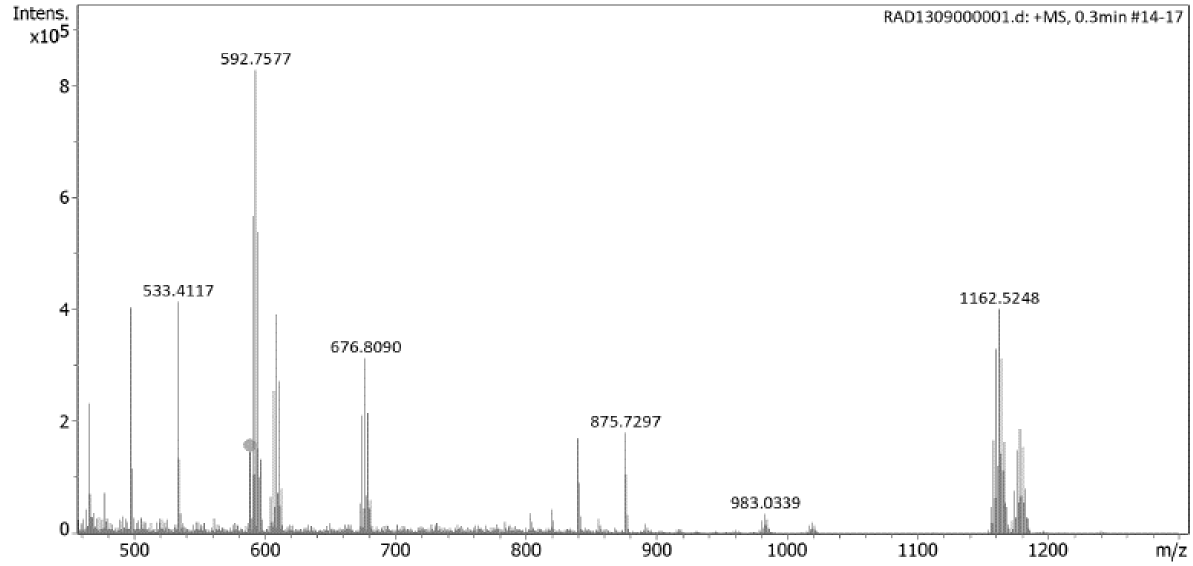

Meas. m/z	#	Ion Formula	m/z	err [ppm]	mSigma	# mSigma	Score	rdb	e⁻ Conf	N-Rule
588.7618	1	C17H14Br4NaO2	588.7620	0.2	4.8	1	100.00	17.0	even	ok
	2	C13H10Br4N6Na	588.7593	-4.3	10.2	2	19.36	18.0	even	ok
	3	C12H14Br4N2NaO4	588.7579	-6.6	20.3	3	4.05	13.0	even	ok

S86: HRESIMS Data of Compound **6**

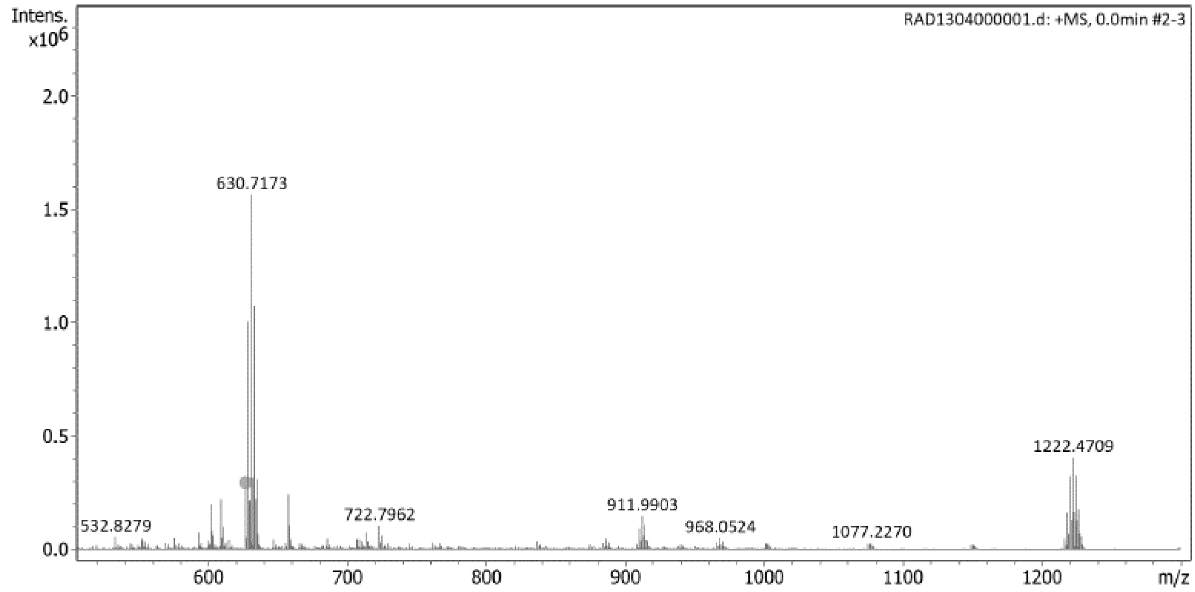

Meas. m/z	#	Ion Formula	m/z	err [ppm]	mSigma	# mSigma	Score	rdb	e⁻ Conf	N-Rule
626.7214	1	C19H12Br4KO2	626.7202	-1.9	7.0	1	100.00	20.0	even	ok

S87: HRESIMS Data of Compound **7**

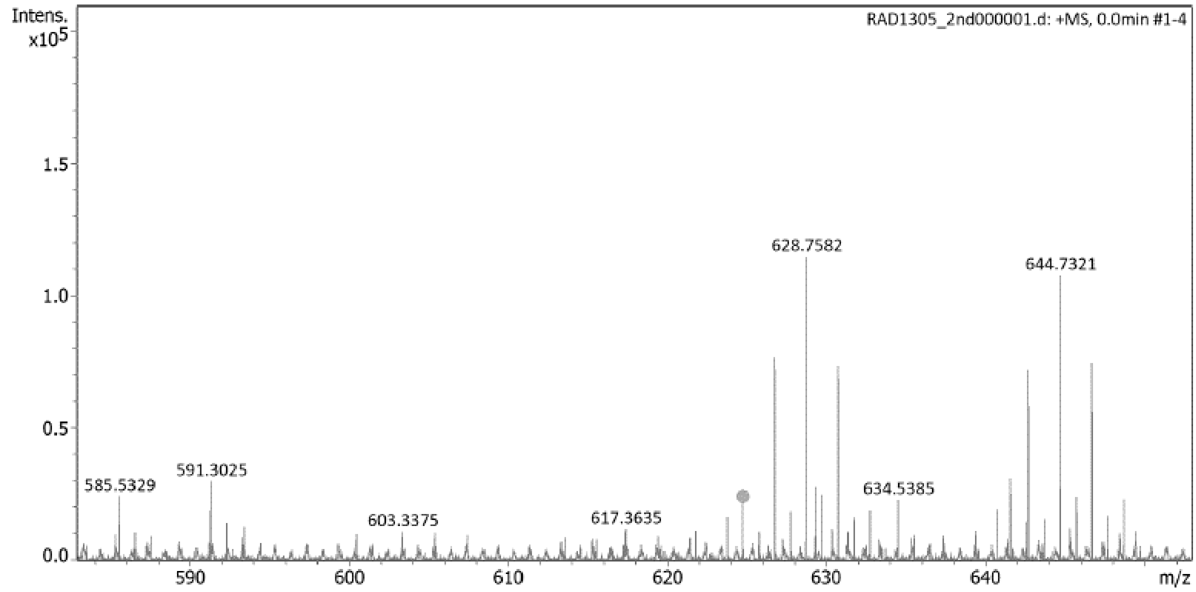

Meas. m/z	#	Ion Formula	m/z	err [ppm]	mSigma	# mSigma	Score	rdb	e⁻ Conf	N-Rule
624.7621	1	C20H14Br4NaO2	624.7620	-0.2	10.9	1	100.00	20.0	even	ok

S88: HRESIMS Data of Compound **8**

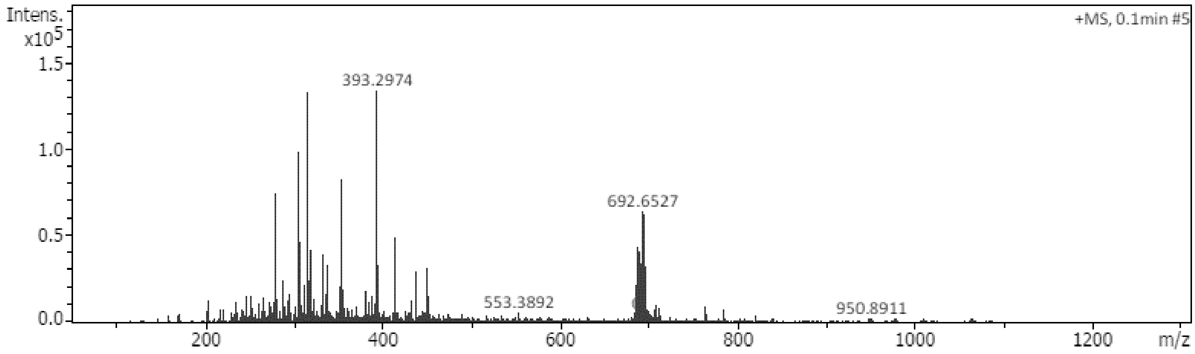

Meas. m/z	#	Ion Formula	m/z	err [ppm]	mSigma	# mSigma	Score	rdb	e⁻ Conf	N-Rule
688.6566	1	C19H11Br5NaO2	688.6568	0.3	7.6	1	100.00	22.0	even	ok

S89: HRESIMS Data of Compound **9**

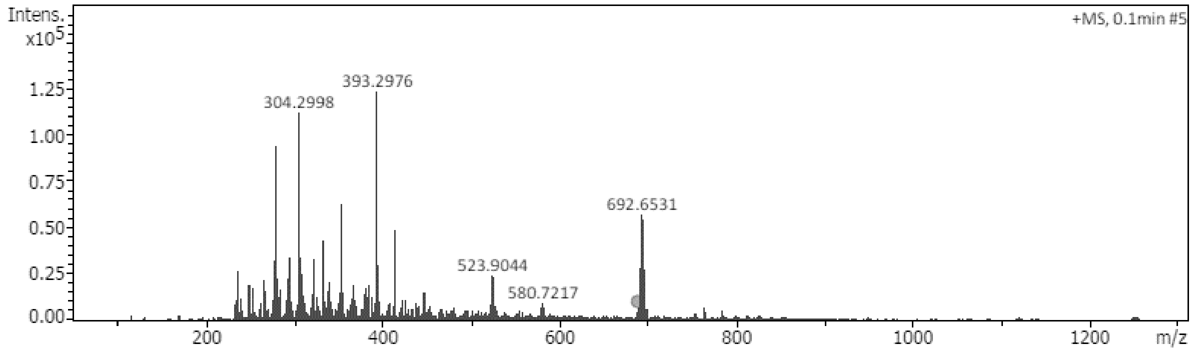

Meas. m/z	#	Ion Formula	m/z	err [ppm]	mSigma	# mSigma	Score	rdb	e⁻ Conf	N-Rule
688.6569	1	C19H11Br5NaO2	688.6568	-0.1	11.5	1	100.00	22.0	even	ok

S90: HRESIMS Data of Compound **10**

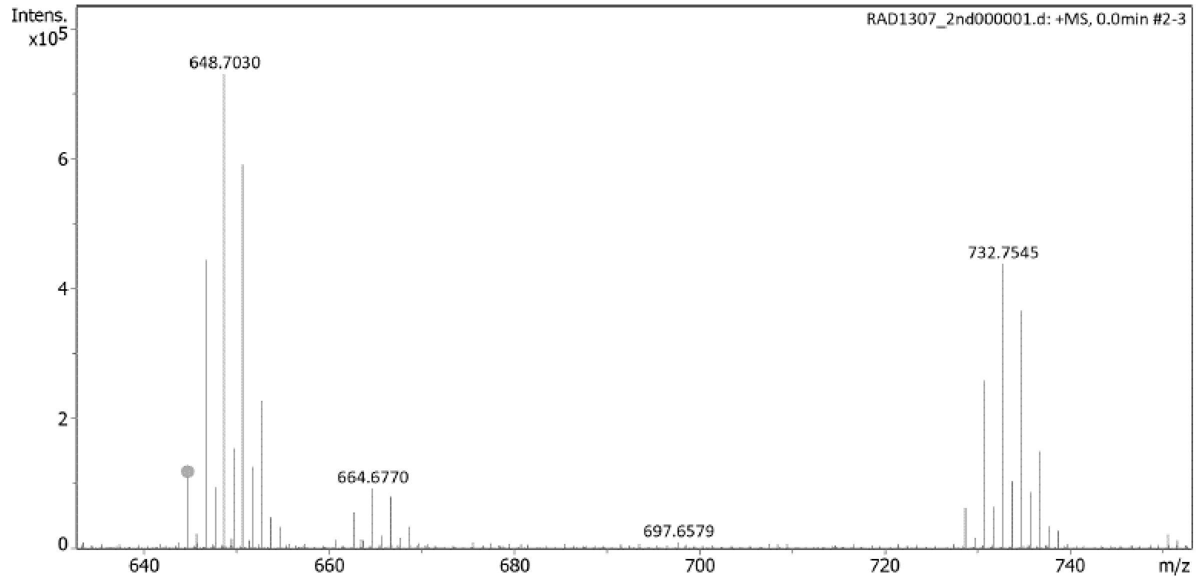

Meas. m/z	#	Ion Formula	m/z	err [ppm]	mSigma	# mSigma	Score	rdb	e⁻ Conf	N-Rule
644.7073	1	C19H11Br4ClNaO2	644.7073	0.0	4.6	1	100.00	23.0	even	ok

S91: HRESIMS Data of Compound **11**

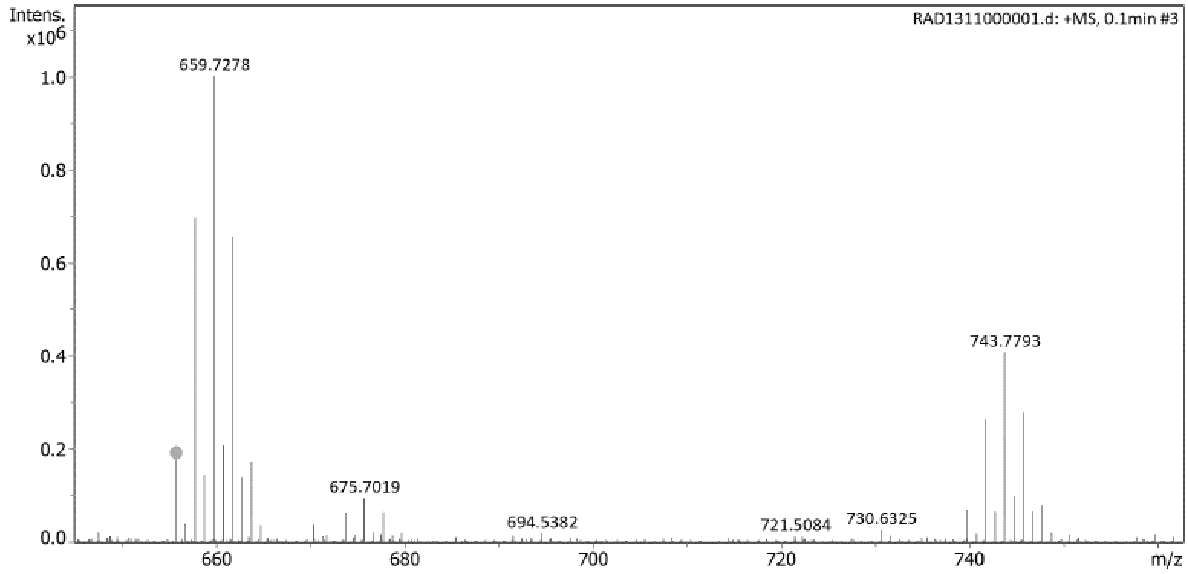

Meas. m/z	#	Ion Formula	m/z	err [ppm]	mSigma	# mSigma	Score	rdb	e⁻ Conf	N-Rule
655.7318	1	C19H11Br4NNaO4	655.7314	-0.7	6.9	1	100.00	21.0	even	ok

S92: HRESIMS Data of Compound **12**

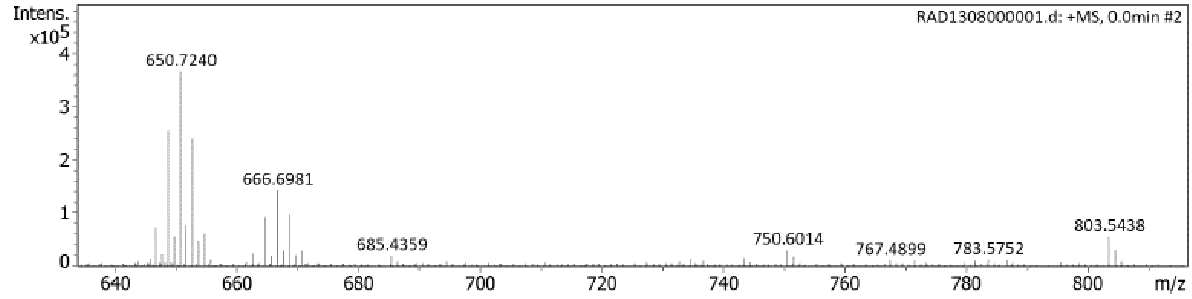

Meas. m/z	#	Ion Formula	m/z	err [ppm]	mSigma	# mSigma	Score	rdb	e⁻ Conf	N-Rule
646.7273	1	C19H10Br4F2NaO2	646.7275	0.3	15.5	1	100.00	20.0	even	ok

S93: HRESIMS Data of Compound **13**

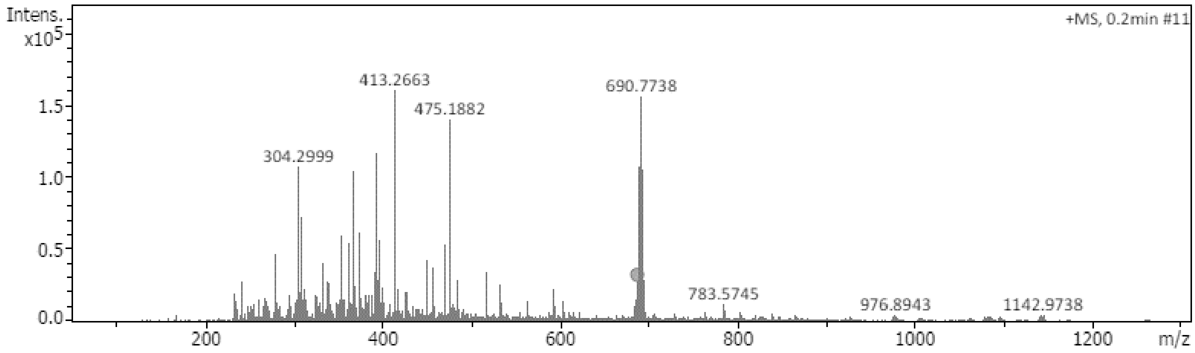

Meas. m/z	#	Ion Formula	m/z	err [ppm]	mSigma	# mSigma	Score	rdb	e⁻ Conf	N-Rule
686.7777	1	C25H16Br4NaO2	686.7776	-0.1	5.3	1	100.00	24.0	even	ok
	2	C18H16Br4N6NaO2	686.7960	26.7	25.1	2	0.00	20.0	even	ok
